# Genome of the small hive beetle (*Aethina tumida*, Coleoptera: Nitidulidae), a worldwide parasite of social bee colonies, provides insights into detoxification and herbivory

**DOI:** 10.1093/gigascience/giy138

**Published:** 2018-12-07

**Authors:** Jay D Evans, Duane McKenna, Erin Scully, Steven C Cook, Benjamin Dainat, Noble Egekwu, Nathaniel Grubbs, Dawn Lopez, Marcé D Lorenzen, Steven M Reyna, Frank D Rinkevich, Peter Neumann, Qiang Huang

**Affiliations:** 1 USDA-ARS, Bee Research Laboratory, BARC-East Building 306, Beltsville, Maryland 20705, USA; 2 Department of Biological Sciences, University of Memphis, 3700 Walker Ave., Memphis, TN 38152, USA; 3 USDA-ARS, Center for Grain and Animal Health, Stored Product Insect and Engineering Research Unit, Manhattan, KS 66502, USA; 4 Agroscope, Swiss Bee Research Center, CH-3003 Bern, Switzerland; 5 Department of Entomology and Plant Pathology, North Carolina State University, 1566 Thomas Hall, Raleigh, NC 27695, USA; 6 USDA, Honey Bee Breeding, Genetics and Physiology Laboratory, 1157 Ben Hur Road, Baton Rouge, LA 70820, USA; 7 Institute of Bee Health, Vetsuisse Faculty, University of Bern, Schwarzenburgstrasse 161, CH-3097, Liebefeld, Switzerland; 8 Honey Bee Research Institute, Jiangxi Agricultural University, Zhimin Avenue 1101, 330045 Nanchang, China

**Keywords:** Coleoptera, pollination, *Apis mellifera*, invasive pest, phytophagy, glycoside hydrolase, honey bee

## Abstract

**Background:**

The small hive beetle (*Aethina tumida;* ATUMI) is an invasive parasite of bee colonies. ATUMI feeds on both fruits and bee nest products, facilitating its spread and increasing its impact on honey bees and other pollinators. We have sequenced and annotated the ATUMI genome, providing the first genomic resources for this species and for the Nitidulidae, a beetle family that is closely related to the extraordinarily species-rich clade of beetles known as the Phytophaga. ATUMI thus provides a contrasting view as a neighbor for one of the most successful known animal groups.

**Results:**

We present a robust genome assembly and a gene set possessing 97.5% of the core proteins known from the holometabolous insects. The ATUMI genome encodes fewer enzymes for plant digestion than the genomes of wood-feeding beetles but nonetheless shows signs of broad metabolic plasticity. Gustatory receptors are few in number compared to other beetles, especially receptors with known sensitivity (in other beetles) to bitter substances. In contrast, several gene families implicated in detoxification of insecticides and adaptation to diverse dietary resources show increased copy numbers. The presence and diversity of homologs involved in detoxification differ substantially from the bee hosts of ATUMI.

**Conclusions:**

Our results provide new insights into the genomic basis for local adaption and invasiveness in ATUMI and a blueprint for control strategies that target this pest without harming their honey bee hosts. A minimal set of gustatory receptors is consistent with the observation that, once a host colony is invaded, food resources are predictable. Unique detoxification pathways and pathway members can help identify which treatments might control this species even in the presence of honey bees, which are notoriously sensitive to pesticides.

## Introduction

**Figure 1:**
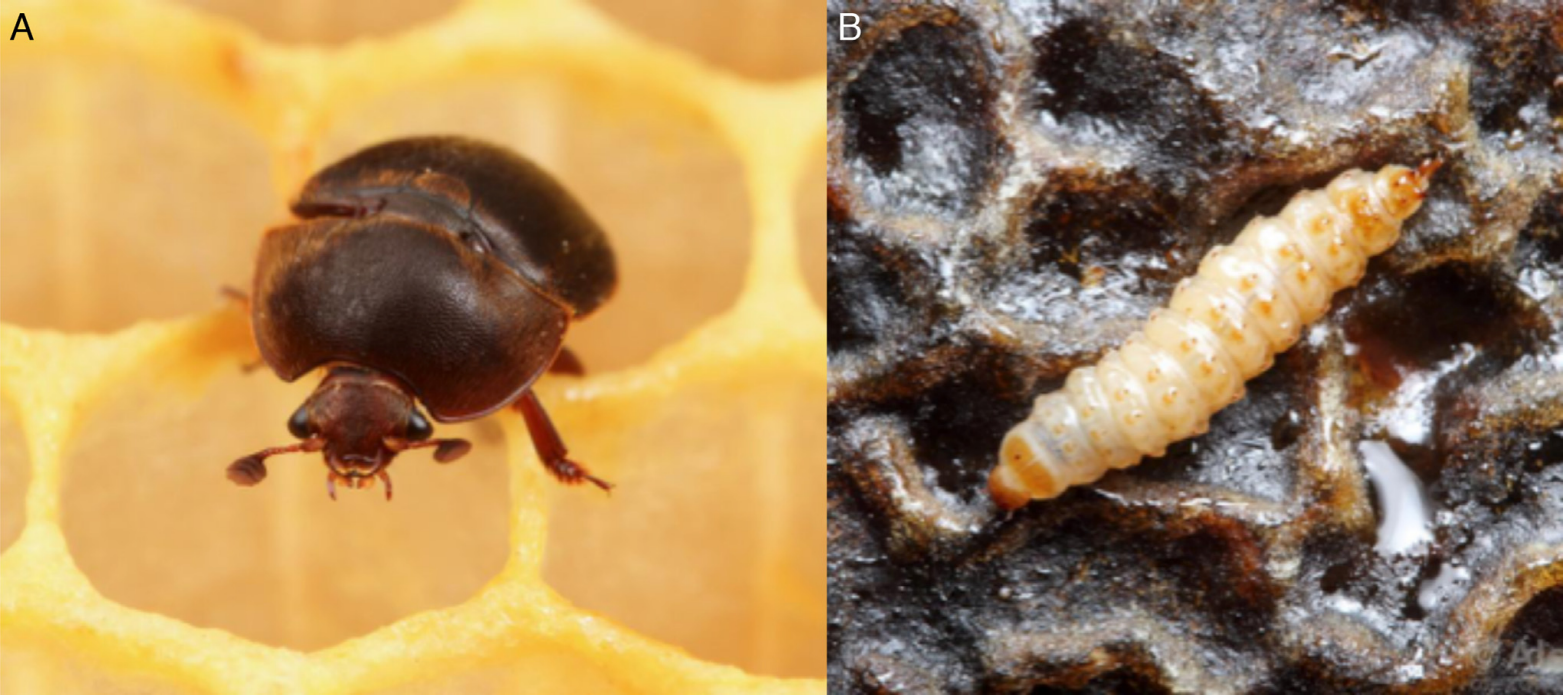
*Aethina tumida* **(A)** adult and **(B)** larva. Photos courtesy of Alex Wild Photography, used with permission.

The small hive beetle (*Aethina tumida* Coleoptera: Nitidulidae, Murray, 1867c, = ATUMI) is a rapidly spreading invasive species originating from sub-Saharan Africa. ATUMI is now found on all continents except Antarctica [1–4]. Outside of its endemic range, it has become an economically important parasite of social bee colonies, including honey bees, bumblebees, and stingless bees [2] (Fig. 1). ATUMI significantly impacts beekeeping and the regulation of honey bees and hive products worldwide. ATUMI eggs are laid within colonies, and developing larvae feed until they leave the colony for pupation [2]. ATUMI pupate in the soil then emerge as adults to infest social bee nests. Once inside the bee nest, adult ATUMI employ a “sit-and-wait” strategy, relying on the resources of the nest for nutrition and shelter until options for successful reproduction arise [2]. ATUMI larvae and adults can feed on a large variety of food sources inside and outside of social bee colonies, including fruits, meat, adult bees, bee brood, and bee food stores (pollen and honey) [1, 5, 6]. Beetles and their bee hosts show an elaborate set of interactions. For example, honey bees attempt to confine adult ATUMI to prisons built from plant resins [6], and beetles can also manipulate guard bees to obtain food by rubbing their antennae against the guarding bees’ mandibles, inducing them to regurgitate food.

ATUMI belongs to the beetle family Nitidulidae (sap beetles; c. 4,500 species), which feed mainly on decaying vegetable matter, overripe fruit, or sap. The Nitidulidae belong to the superfamily Cucujoidea (sap, bark, and fungus beetles), which is either the sister group of the Phytophaga (leaf beetles, weevils, longhorned beetle, and their relatives [7]; the most species-rich radiation of plant-feeding animals on Earth with >125,000 described species) or forms a paraphyletic clade subtending the Phytophaga [8, 9]. In the latter case, the Phytophaga are derived from within Cucujoidea. Interestingly, the trophic habits of Nitidulidae may therefore represent a transitional stage from mycophagy, saprophagy, and detritivory (the typical habit(s) of most Cucujoidea and its containing clade, series Cucujiformia) to phytophagy (feeding on plants), the typical trophic habit of Phytophaga. Comparative studies of the ATUMI genome may therefore provide new insights into the evolution and genomic basis of phytophagy in beetles.

To date, just 10 beetle genome assemblies have been released [10], of which only 7 are published, despite there being >400,000 described beetle species. These are: *Tribolium castaneum* (red flour beetle, TCAST; Tenebrionoidea: Tenebrionidae: Tenebrioninae [11]), *Anoplophora glabripennis* (Asian longhorned beetle, AGLAB; Chrysomeloidea: Cerambycidae: Lamiinae [12]), *Dendroctonus ponderosae* (mountain pine beetle, DPOND; Curculionoidea: Curculionidae: Scolytinae [13]), *Hypothenemus hampei* (coffee berry borer beetle, HHAMP; Curculionoidea: Curculionidae: Scolytinae [14]), *Oryctes borbonicus* (Reunion Island scarab beetle, OBORB; Scarabaeoidea: Scarabaeidae: Dynastinae [15]), *Onthophagus taurus* (bull headed dung beetle, OTAUR; Scarabaeoidea: Scarabaeidae: Scarabaeinae; unpublished), *Nicrophorus vespilloides* (burying beetle, NVESP; Staphylinoidea: Silphidae: Silphinae [16]), *Agrilus planipennis* (emerald ash borer, APLAN; Buprestoidea: Buprestidae: Agrilinae; unpublished), *Leptinotarsa decemlineata* (Colorado potato beetle, LDECE; Chrysomelidae: Chrysomelinae: Doryphorini [17]), and *Pogonus chalceus* (salt marsh beetle, PCHAL; Carabidae: Trechinae: Pogonini; unpublished). The ATUMI genome described here joins this group as the only representative from the superfamily Cucujoidea.

A robust reference genome assembly comprised of 234 million bp was used to identify and annotate 14,076 protein-coding genes, more than 3,000 additional transcribed features and a strong complement of repetitive DNAs, tRNAs, and transposable elements. The described protein-coding genes provide strong candidates for core metabolism and development and suggest that these beetles, like their honey bee hosts, rely on olfactory cues and less so on chemosenses related to taste. An analysis of protein groups involved in insecticide metabolism reveals a large repertoire of detoxification enzymes to mediate xenobiotic interactions. The described resources will be useful for both chemical and non-chemical approaches for controlling this key pest of honey bees.

## Results and Discussion

### Genome traits, genetic diversity, and phylogenetic analysis

We generated a genome assembly of 234 Mbp comprised of 3,063 contigs (contig N_50_ = 298 kb; Table 1). The genome sizes of sequenced and assembled beetle species vary greatly from 160 Mbp to 1.1 Gbp. The size of the ATUMI genome assembly is larger than that of the red flour beetle (165.9 Mbp) but much smaller than the more derived Asian longhorned beetle (707.7 Mbp). A total of 1,293,015 heterozygous single-nucleotide polymorphism (SNP) positions were identified, with an average density of 1 SNP per 181 bp. SNP density was significantly different across contigs (*T* test, *P* < 0.01). This pattern was not related to contig size. Overall, 60.2% of SNPs occurred on contigs with annotated genome features and 22.5% were within gene regions.

**Table 1:** Assembly statistics of the small hive beetle genome

Illumina (genome coverage)	535
Pacific Biosciences (genome coverage)	50
Assembly size (Mbp)	234.3
Number of contigs	3063
Largest contig (Kbp)	2,683.7
Smallest contig (Kbp)	1.26
N50 (Kbp)	298.8
Number of contig > 10 Kbp	2,236
Number of contig > N50	192
Number of protein-coding genes	14,076
Number of mRNAs	17,463
Density of SNPs (bps per SNP position)	181
Density of microsatellites (loci per Kbp)	8.23

The National Center for Biotechnology Information (NCBI) eukaryotic genome annotation pipeline [18] proposed 14,076 protein-coding genes and 17,436 mRNA models. When our previous RNA sequencing (RNA-seq) reads were aligned to the genome assembly alongside the predicted gene models, 99.73% of the predicted mRNA models and 99.65% of the predicted protein-coding genes were supported. It is possible that the 64 protein-coding genes undetected by RNA-seq were not expressed, expressed too briefly, or not captured in our pooled RNA samples. Alternatively, these might reflect partial or inaccurate gene models or pseudogenes that are no longer functional in this beetle.

**Figure 2:**
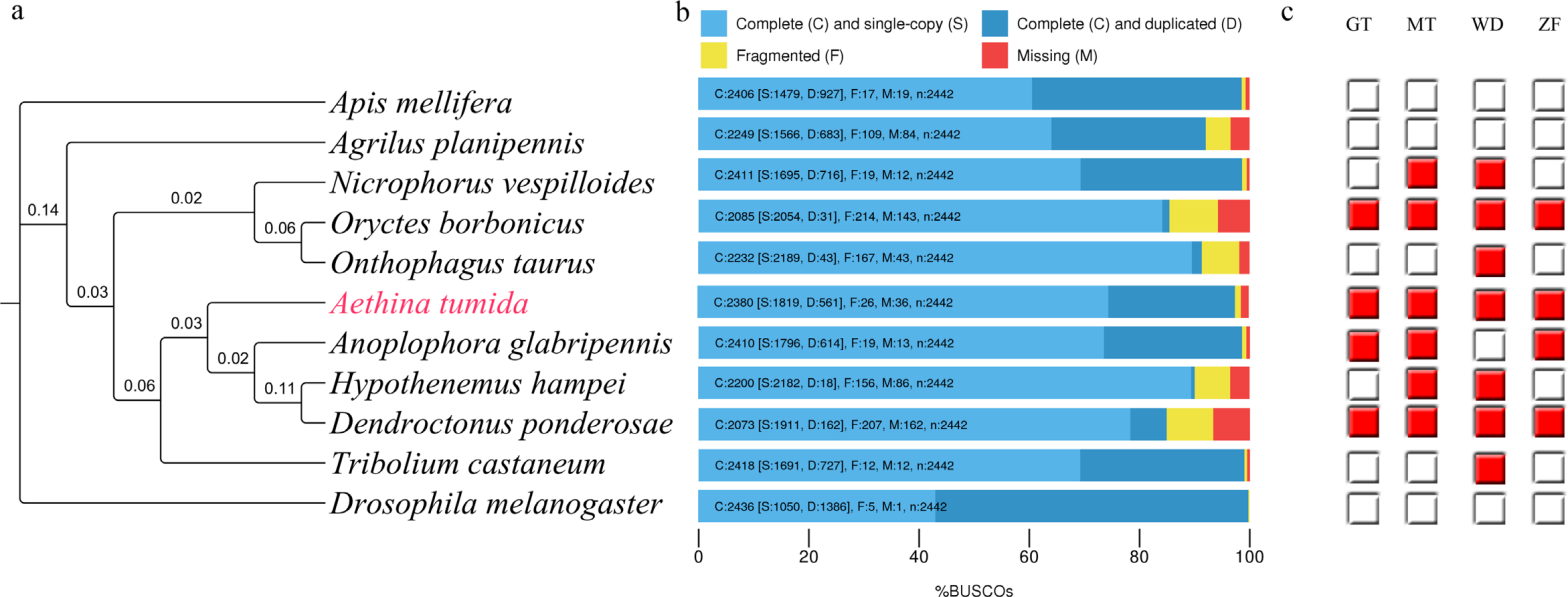
Phylogenetic tree and estimated completeness of the genomes of 11 insect species. **(a)** The phylogenetic tree was constructed on protein sequences of 181 single-copy orthologs shared among all 11 insect species. All nodes have 100% bootstrap support. AMELL and DMELA were used as outgroups. Branch lengths are shown for each node. **(b)** Completeness of official protein sets of each insect species were assessed by aligning to the Endopterygota sets of benchmarking universal single-copy orthologs (BUSCOs). For ATUMI, 97.5% of complete BUSCOs were found. **(c)** The pervasiveness of gene loss during endopterygote evolution. From the domain counts of lost BUSCOs, methyltransferase (MT), glycosyltransferase (GT), and leucine-rich repeats (LRR) are among the top 5% of total domains and are commonly lost from multiple species. Beta-transducin repeats (WD) and zinc finger (ZF) red boxes indicate that the gene is lost, while white boxes indicate that the gene is maintained in each species.

By aligning the ATUMI official protein set against 2,444 core Endopterygota Benchmarking Universal Single-Copy Orthologs (BUSCOs), 97.5% of complete BUSCOs were found (Fig. 2b). We further aligned the ATUMI genome assembly against Endopterygota set of BUSCOs, and 92.8% of complete BUSCOs were found (Supplementary File 1). The results suggest a high level of completeness in the genome assembly, as well as the official set of gene models. By comparing single-copy orthologs among the sequenced beetles (ATUMI, TCAST, DPOND, AGLAB, ATAUR, APLAN, HHAMP, NVESP), honey bees (AMELL), and *Drosophila melanogaster* (DMELA), 181 shared ortholog groups were found. A phylogenetic tree was built by concatenating these shared 181 orthologous groups (Fig. 2a). These results suggest that ATUMI is sister to TCAST and the Asian longhorned beetle (AGLAB). OrthoDB [19] orthology delineation revealed that ATUMI has 7,066 conserved orthologous groups with beetles and 4,554 orthologous groups shared with 10 additional insect species.

### Loss and duplication of BUSCO genes from the small hive beetle genome

**Figure 3:**
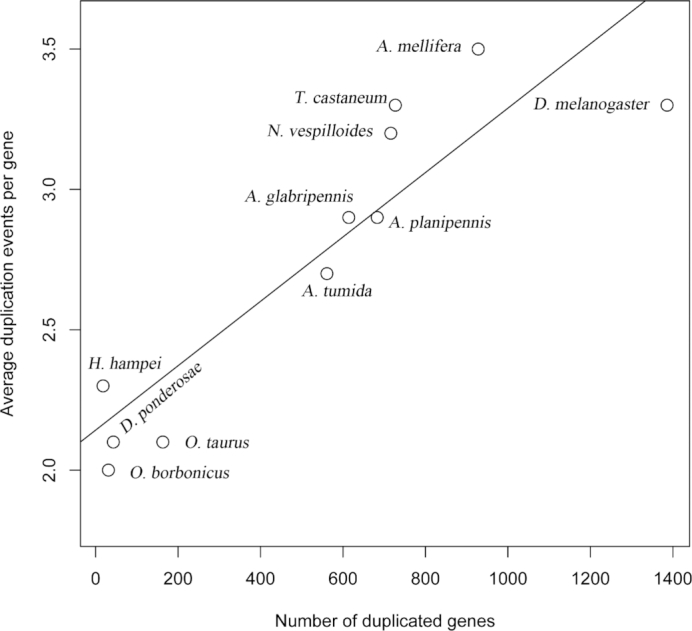
Gene duplication events plotted against the average gene duplication event per gene. The protein sets of the 11 studied beetle species, as well as honey bee and fruit fly, were searched against the Endopterygota BUSCO set using Basic Local Alignment Search Tool (BLAST). Redundant proteins (including recent paralogs and those with known alternative splicing) were used to quantify the average number of duplication events per gene in each species.

The duplication and absence of core genes, including those represented by BUSCO, could represent important evolutionary changes in species or in lineages (Fig. 3). A complete protein set of 11 insect species was used for alignment against the ATUMI BUSCO candidates. We found 337 core Endopterygota BUSCOs that were either fragmented or completely lost from at least two beetle genomes. We mapped the common ancestor sequences of these 337 missing orthologs and the full set of 2,442 Endopterygota BUSCOs to the Pfam database. Among the “lost” orthologs, 1,094 protein domains were found; among 2,442 Endopterygota orthologs, 4,632 protein domains were found. By comparing the count distribution of each domain between lost orthologs and overall orthologs, no significant difference was found (Pearson Chi-square test, *P* > 0.05). Among the lost orthologs, a methyltransferase, a glycosyltransferase, and two proteins with beta-transducin repeats and zinc finger domains, respectively, showed the highest counts and were also absent from at least four beetle species (Fig. 2c).

### Glycoside hydrolases

Glycoside hydrolases (GHs) are important enzymes that aid in the digestion of plant cell walls and carbohydrates in insects [20]. However, GHs can also contribute to remodeling of the peritrophic matrix (PM) [21], lysosomal enzyme activity, glycoprotein oligosaccharide catabolism, immune response, and growth and development [22, 23]. A limited diversity of GH families was identified in the ATUMI genome when compared to other beetles. While phytophagous insects such as AGLAB [7], DPOND [13], and HHAMP [14] harbored anywhere from 19–24 different GH families represented by 101–199 genes, only 14 GH families represented by 91 genes were identified in the ATUMI genome. Only OBORB, whose diet is unknown [15], had a lower GH family diversity and GH copy number, with 13 different families represented by 47 different genes. No GH families unique to ATUMI were identified (Supplementary Files 2 and 3).

**Figure 4:**
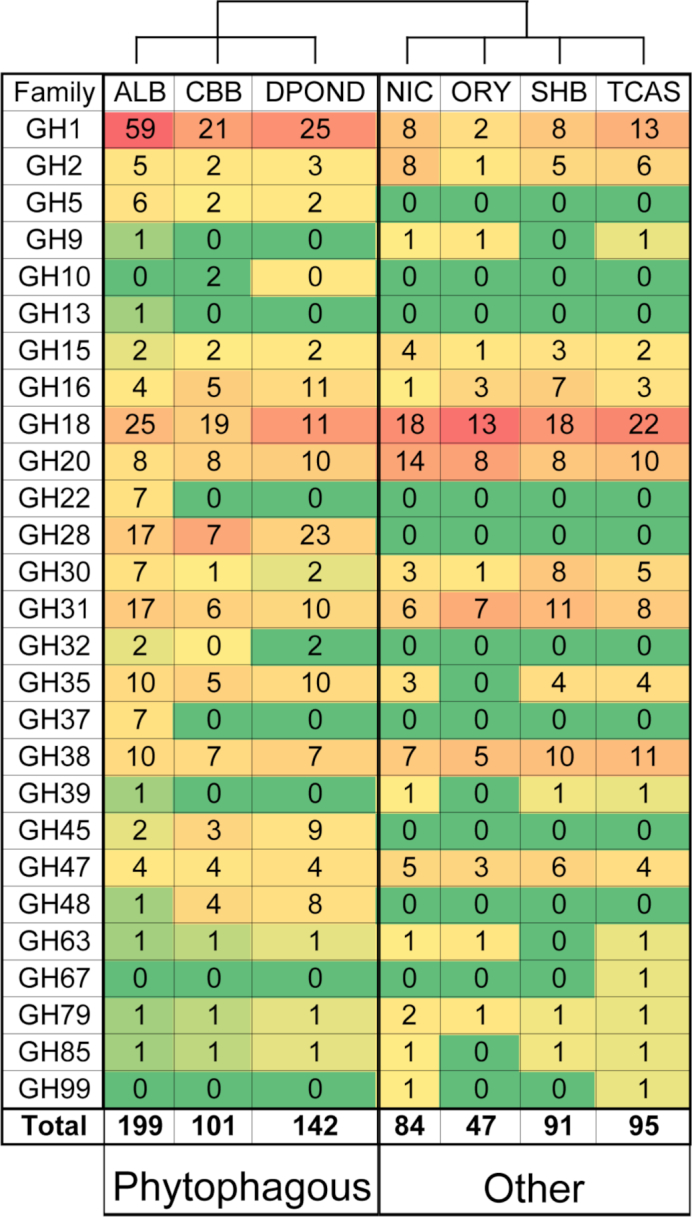
Glycoside hydrolase (GH) family copy numbers identified from beetle genomes. Genes coding for GHs were identified using Pfam domain assignments [24], and genome assemblies and coding gene predictions were obtained from NCBI (GenBank accession numbers: GCA_000390285.1 ALB, GCA_000355655.1 DPOND, GCA_001412225.1 NIC, GCA_001443705.1 ORY, GCA_000002335.3 TCAS) with the exception of CBB, which was downloaded from [25]. Families are color coded from green to red based on their relative abundance (total count/total number of GH genes), with red representing GH families that are highly abundant (≥25% of the total GH genes) and green representing GH families of lesser abundance (≤0.01%). Notably, the GH profiles of ATUMI and TCAST (neither of which feed on living plant material) differ strongly from the GH profiles of the phytophagous beetles, even though they all belong to the same infraorder, suggesting that diet, in part, might be driving the differences in GH family members and copy numbers. ALB = Asian longhorned beetle (*A. glabripennis*); CBB = Coffee berry borer (*H. hampei*); DPOND = Mountain pine beetle (*D. ponderosae*); NIC = burying beetle (*N. vespilloides*); ORY = scarab beetle (*O. borbonicus*); SHB = small hive beetle; and TCAS = red flour beetle (TCAST).

Using orthology searches, five orthogroups containing GHs were more prominent in the ATUMI genome compared to other beetles, and two GHs lacked orthologs in other beetle genomes. The more prominent orthogroups contained genes with the highest scoring Basic Local Alignment Search Tool (BLAST) P matches to GH 30 glucosylceramidase (eight copies; sphingolipid metabolism), uncharacterized GH 31 α-glucosidases (five copies), GH 16 β-1,3-glucan binding protein (five copies; exoskeleton and/or PM remodeling), GH 38 lysosomal α-mannosidase (five copies), and GH 18 chitinase (three copies). Interestingly, unigenes coding for GH 18 (20 copies), GH 31 (11 copies), and GH 38 enzymes were also among the most prominent GH families in the ATUMI genome (Fig. 4). Generally, GH 38 copy numbers were high in the ATUMI genome relative to other beetles and were exceeded only by TCAST. In contrast, copy numbers of GH 18 and 31 genes were similar to those found across other beetles. Additionally, two GH genes encoded by the ATUMI genome lacked orthology to other beetle GHs, including a GH 2 family gene coding for β-mannosidase and a GH 35 family gene coding for β-galactosidase. Other beetles code for GH 2 β-mannosidases and GH 35 β-galactosidases, so it is unclear why these two genes were not assigned to orthogroups. However, the evolutionary history of genes coding for GH enzymes is complex and it may be difficult to assign orthologs in some cases.

Overall, ATUMI lacked a diverse and expansive repertoire of GHs relative to phytophagous beetles, which may reflect the ATUMI diet. Pollen generally contains high concentrations of the monosaccharides glucose and fructose [26], which are used directly for ATP production by the glycolysis pathway (glucose) or after phosphorylation by fructokinase (fructose). Therefore, although pollen can also contain starch, sucrose, and small amounts of pectin [26], digestion of more complex carbohydrates may not be necessary, requiring a less expansive repertoire of GH enzymes relative to phytophagous beetles. Supporting this hypothesis, genes coding for enzymes capable of digesting starch were identified (α-amylase), but genes coding for invertases and polygalacturonases for sucrose and pectin digestion could not be identified. Alternatively, microbial symbionts harbored by ATUMI may facilitate the breakdown of these polysaccharides as has been observed previously in their honey bee hosts, which share a similar diet [27].

### Gustatory receptors

G-protein-coupled receptors (GPCRs) comprise a large family of integral membrane proteins found in cells of all eukaryotes [28]. GPCRs function to detect extracellular stimuli and activate cellular signal transduction pathways that ultimately lead to physiological and behavioral responses. Gustatory receptors (GRs) belong to novel arthropod GPCR gene superfamilies, which are phylogenetically unrelated to mammalian taste receptor genes and distinct from related insect odorant/pheromone receptor genes [29]. GRs are important components of an organism's sensory machinery; an animal's ability to distinguish between nutritious, noxious, and possibly toxic compounds is a matter of life or death. Sensory machinery has been honed over evolutionary time and has given rise to receptors binding either sweet (attractive) or bitter (aversive) tastants, [30, 31]. An amino acid substitution in a ligand-binding region may affect the range at which different ligand's receptors may bind, particularly for GRs perceiving sugars [32].

**Figure 5:**
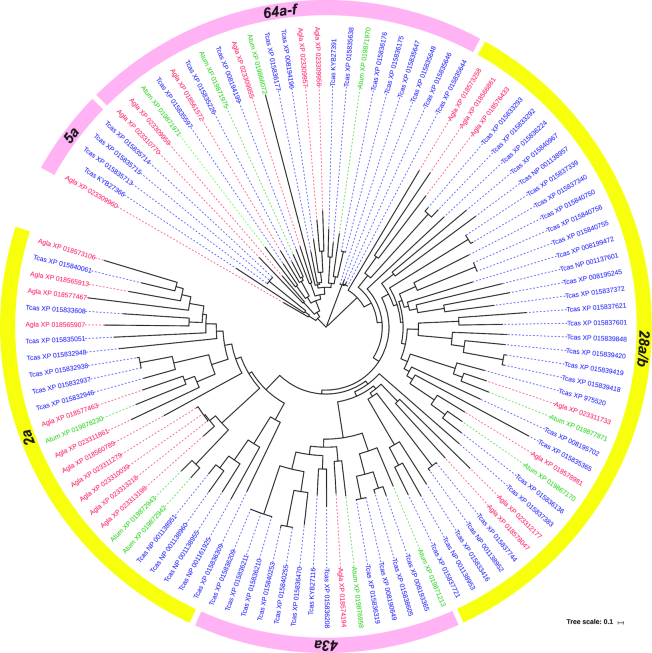
Maximum likelihood cladogram for gustatory receptor genes from three coleopteran species. The small hive beetle, *Aethina tumida* (*Atum*; green labels/lines), the Asian longhorned beetle, *Anoplophora glabripennis* (*Agla*; red labels/lines), and the red flour beetle, *Tribolium castaneum* (*Tcas*; blue labels/lines). Individual genes are labeled with species identifier and GenBank accession number. Scale bar for branch lengths represents 0.1 amino acid substitutions per site. Ring around cladogram indicates gene families coded for perceiving bitter (yellow) and sweet (pink) tastants.

GR genes fall into four main clades that correspond with perception of different tastants (sweet or bitter; Fig. 5). Designations of the type of substance perceived by these receptors can be inferred from other taxa (e.g., *Drosophila* sp.) and the positions of uncharacterized proteins within the cladogram. A group of apparently highly conserved genes encoding proteins for perceiving sweet substances (clades *5a* and *64a-f*) is separate from other groups that show higher sequence variability; a pattern seen in other studies (e.g., [33]). Proteins of *GR5a* and *GR64a-f* can form heterodimeric complexes at receptor sites and may or may not be necessary together for perception of different sugars [34, 35]. ATUMI appears to lack a *GR5a* gene (Table 2; Fig. 5), suggesting this gene may not be necessary for perceiving sweet tastants. In this group of ATUMI *GR*s, it is interesting to note that one candidate with a very long branch length (XP_019866072) encodes a 379 amino acid protein derived from three exons and has a very long intron. It is unclear why this gene is so distinct compared to the relatively highly conserved sequences for other related *GR* genes.

**Table 2:** Number of gustatory receptor (GR) genes from major groups for three coleopteran species, the small hive beetle (ATUMI), AGLAB, and TCAST, and their putative coding for detecting either bitter or sweet tastants

Species	Gustatory receptor group						Tastant type	
	2a	5a	28a/b	43a	64a-f	Total	Bitter	Sweet
AGLAB	11	1	7	1	6	26	19	7
ATUMI	3	0	2	2	4	11	5	6
TCAST	12	3	30	12	14	71	42	29

A major finding is that ATUMI has a substantially depauperate repertoire of *GR* genes compared to both AGLAB and TCAST (Fig. 5). This low number of *GRs* in ATUMI is more likely the result of a lack of gene expansion in particular lineages or subfamilies of *GRs* rather than gene loss. A similarly small number of *GRs* is evident in the honey bee genome [36]. In that species, the relatively reduced *GR* gene repertoire may be a consequence of restricted dietary breadth (specialist on pollen and nectar) and also possibly arises from the processing of collected foods by adult workers and microbes, which may reduce the load of plant secondary compounds. AMELL larvae are fed processed foods by attending nurse bees, so they may not need an expansive repertoire of *GR*s to discriminate among different tastants [33]. Because of the close affinity of ATUMI with honey bees, including sharing a similar diet, the evolutionary pressures limiting expansion of *GRs* in ATUMI may be similar. As an example, TCAST, a dietary generalist, shows a significant expansion in the *GR28a/b* gene complex (Table 2); genes in this complex may be important for perceiving plant secondary compounds [37].

Stemming from their importance to insect biology, *GRs* have been characterized from genomic and transcriptomic studies for a number of economically important insects or those having an ecological and/or epidemiological significance, including TCAST [11], AGLAB [12], and now ATUMI (this study). Understanding the chemosensory abilities of insects, particularly pest insects, is important for designing possible means of control that target the insect's ability to find and/or distinguish among nutrients or to detect poisons and/or developing baits containing insecticides formulated with highly attractive substances.

### Voltage-gated sodium channel

The voltage-gated sodium channel (Na_v1_) is responsible for generating action potentials in neurons. Sodium channel modulator insecticides such as pyrethroids and DDT act on the Na_v1_ channel by maintaining the open state of the channel via interactions with two proposed binding sites [38, 39]. A diverse collection of mutations in Na_v1_ has been identified in many populations of pyrethroid-resistant pests, and neurophysiological studies of heterologously expressed channels have confirmed the role of these mutations in pyrethroid resistance [40].

A single transcript and protein were predicted for Na_v1_ from the ATUMI assembly. However, Na_v1_ is known to possess optional and alternative exons in most insects [41–43]. Alternative exon use diversifies the physiological repertoire of the sodium channel and may affect insecticide sensitivity [44]. Further cloning experiments to determine the actual optional and alternative exon use in ATUMI Na_v1_ should be informative.

A large number of mutations in Na_v1_ have been associated with target site resistance to pyrethroids and DDT [40]. We did not identify such mutations in the predicted ATUMI Na_v1_ nor is this species known to be resistant to these insecticides. Therefore, this sequence serves as a reference for a susceptible target site for pyrethroids and DDT and a tool for developing molecular diagnostic assays to monitor changes in resistance allele frequency.

### Acetylcholinesterase

Acetylcholinesterase (Ace) cleaves acetylcholine (ACh) to regulate the effect of the neurotransmitter in the synaptic cleft. Ace is the target of organophosphate (OP) and carbamate insecticides, and mutations in Ace result in target-site insensitivity to these two insecticide classes [45, 46].

ATUMI is predicted to possess active forms of both Ace1 (XP_019871456.1) and Ace2 (XP_019866656.1) (Supplementary File 2). Ace mutations involved in OP resistance [46, 47] are found to be in the susceptible state in the predicted Ace proteins of ATUMI (Table 3). In the cases where an alternative amino acid was found in ATUMI (i.e., ATUMI_Ace2 position 198), that same amino acid was seen in other insects that were presumably sensitive to OPs, so it does not likely confer reduced OP sensitivity. Ace2 performs primary acetylcholine esterase activity in honey bees, while Ace1 is the primary enzyme in beetles and most other insects [48]. Therefore, identifying compounds that only inhibit ATUMI_Ace1 may provide a level of ATUMI-specific control.

**Table 3:** Evaluation of resistance mutations in acetylcholine esterase and their status in ATUMI

*Torpedo* Ace position	ATUMI_Ace1 position	Resistance mutations	ATUMI_Ace1 state
119	189	G247S, G119D	G
128	198	D237E	D
201	270	A302S	A
227	296	G265A, G262A	G
290	358	F290V	F
331	399	S431F, F445W, F439C	F
*Torpedo* Ace position	ATUMI_Ace2 position	Resistance mutations	ATUMI_Ace2 state
78	114	F139L, F115S	F
82	118	E81K	E
129	177	I161V/T	I
151	198	V180L	I
227	280	G265A, G262A/V	G
238	290	S291G	T
290	358	F330Y, F237Y	F
328	383	G365A, G368A	G
396	452	G488S	G

*Torpedo* Ace position number and the resistance mutations are described in [46]

### ATP-binding cassette proteins

ATP-binding cassette (ABC) proteins are a large, diverse family of proteins found in most organisms, from bacteria to plants and vertebrates. Most ABC proteins engage in active transport of molecules across cell membranes. This family of transporters is perhaps most notable for moving toxins into or out of cells, which has resulted in the identification of several of these proteins playing a role in the resistance of cancer cells to multiple drug treatments (multidrug resistant). So it is not surprising that some of these proteins have been identified as having roles in insect susceptibility or resistance to certain insecticides (reviewed by [49]). In spite of their importance for shaping pest control methods, these genes are under-studied in insects, with few having been fully characterized in any species. The status of ATUMI as a pest of beehives makes it important to understand what role ABC genes may play in how beekeepers control this species.

**Figure 6:**
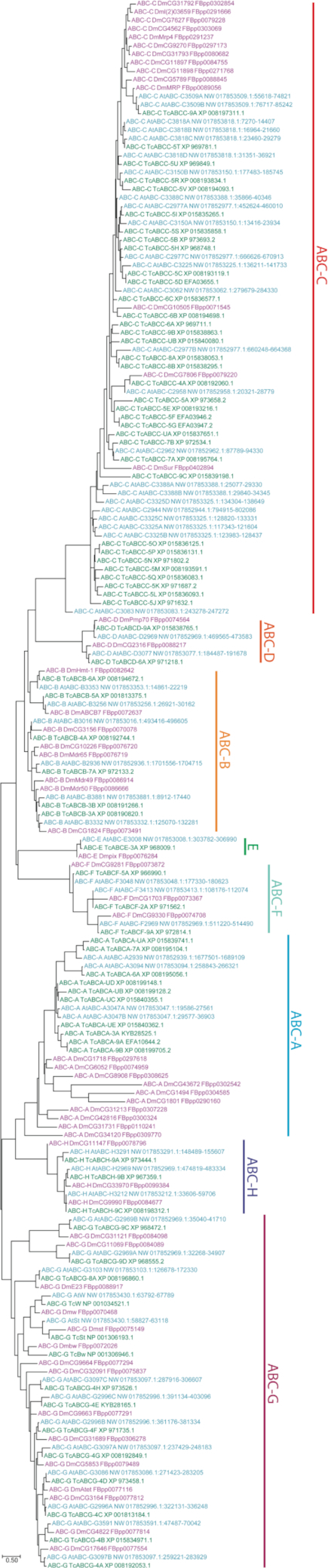
Maximum likelihood phylogenetic tree of ABC proteins from ATUMI (At), TCAST (Tc), and DMELA (Dm). ATUMI genes are marked in blue, TCAST in green, and DMELA in purple. ABC subfamilies are indicated with colored lines to the right of the tree. Names for DMELA proteins were taken from Flybase [52] and include the Flybase number for reference. TCAST names were taken from the two articles in which the genes were identified [50, 51], with the NCBI Refseq accession number provided for reference. ATUMI names were generated for this article by combining the subfamily of the identified sequence with the scaffold on which the encoding gene may be found. If multiple ABC genes of a particular subfamily were found on the same scaffold, the sequences were given an additional letter designation based on their relative location, reading left to right on the scaffold as shown in WebApollo. For reference, the scaffold number and base coordinates for the gene have also been included.

The beetle genetic model organism, TCAST, has had its full suite of ABC-family genes identified through a combination of RNA-seq and genomic analysis. In this species, 74 genes have been identified (Table 4) [50, 51]. The translation products of these genes were used to query the ATUMI genome, in which 56 ABC genes were identified (Table 4). In most respects, the makeup of ABC genes in ATUMI resemble those found in TCAST—both species have identical numbers of ABC-B, D, E, F, and H subfamily members. Indeed, the numbers of members in the D-F and H subfamilies are highly conserved, with DMELA having the same number, and clear one-to-one relationships can be seen in these subfamilies among the members from each species (Fig. 6). It should be noted that members of subfamilies E and F do not function as transporters and are highly conserved in number and sequence between insects and humans. Moreover, RNAi targeting ABC-E and one of the ABC-F genes in TCAST resulted in complete mortality, suggesting that the critical cellular roles of these genes may also be conserved. The ABC-B subfamily also appears well conserved and may be worth additional scrutiny in ATUMI since this subfamily has been associated with resistance to several classes of pesticides in multiple species [49].

**Table 4:** Numbers of ABC genes in each species, by subfamily

Species	Subfamily								Total
	**A**	**B**	**C**	**D**	**E**	**F**	**G**	**H**	
ATUMI	4*	6	24	2	1	3	13*	3	56
TCAST	10	6	35	2	1	3	14	3	74
DMELA	10	8	14	2	1	3	15	3	56

* Lower counts discussed in text.

ATUMI differed from TCAST in member counts for three ABC subfamilies (Table 4). The first was subfamily A, for which only 4 members could be identified in ATUMI, relative to the 10 found in TCAST and DMELA, a number roughly consistent across the insects. However, it is important to note that ABC-A genes are fairly large full transporters and, as such, are often complex and difficult to identify in full. So, it is likely that some of the ABC-A genes are either not present in the current genome assembly or are too fractured to recognize. It is also interesting to note that the beetle ABC-A genes appear to segregate from those of DMELA (Fig. 6), suggesting possible pesticide targets against ATUMI, which may not harm other species, including pollinators.

TCAST appears to have one more ABC-G gene than does ATUMI. Specifically, ATUMI appears to lack an ortholog of the well-studied DMELA eye-pigment transporter known as Brown (Bw). However, it has been well documented that Bw orthologs have substantially diverged in TCAST [51]. It is possible that similar divergence has also prevented clear identification of a Bw ortholog in ATUMI. Otherwise, most other ABC-G genes have clear one-to-one orthologs in all three species (Fig. 6).

The largest subfamily, the ABC-C genes, is known to play roles in multidrug resistance in human disease, and some have been associated with Bt resistance in lepidopterans [49]. ATUMI has fewer ABC-Cs than TCAST but more than DMELA. At first, this might suggest a beetle-specific expansion as well as a TCAST-specific expansion. Indeed, there is a suite of expansions that may be beetle specific (Fig. 6), although comparisons to more species would be required to confirm this. However, each species also appears to have its own expansions; TCAST and ATUMI expansions are often tandem, as can be seen by the number of genes found on the same linkage groups/scaffolds (Fig. 6). Indeed, there are surprisingly few clear one-to-one orthologous relationships, suggesting rapid evolution of ABC-C genes to fill species-specific needs. To understand ATUMI responses to pesticides, these ATUMI-specific expansions may be worth additional study.

### Gluthatione-S-transferase

Gluthatione-S-transferases (GSTs) are conjugases that bind glutathione to a wide variety of substrates such as plant allelochemicals, insecticides, reactive oxygen species, and metabolic products that can provide detoxification, antioxidant, excretion, and transport functions [53–55]. Insect GSTs are widely studied due to their role in insecticide resistance [56]. Genomic analyses show that insects possess between 10 and 41 genes that encode GSTs distributed across eight classes (i.e., Delta, Epsilon, Omega, Sigma, Theta, Zeta, Microsomal, and Unclassified) [57].

**Figure 7:**
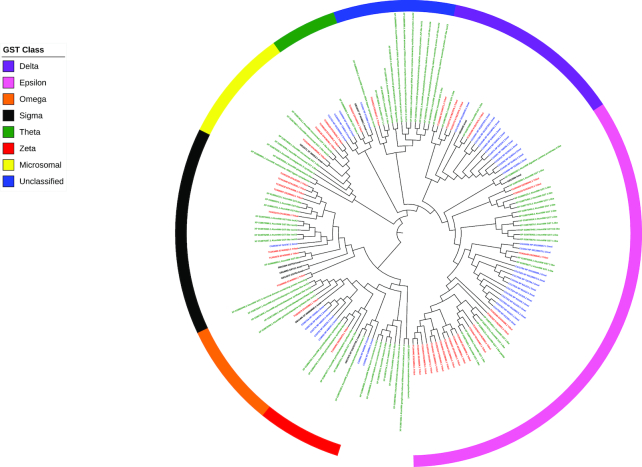
Maximum likelihood phylogenetic tree of glutathione-S-transferase (GST) proteins. The bootstrap consensus tree inferred from 1,000 replicates is taken to represent the evolutionary history of the taxa *A. tumida* (ATUMI) in green, *A. mellifera* (AMELL) in black, *D. melanogaster* (DMELA) in blue, and *T. castaneum* (TCAST) in red, identified manually using the Uniprot and Pfam databases. Branches corresponding to partitions reproduced in less than 50% bootstrap replicates are collapsed. Initial tree(s) for the heuristic search were obtained automatically by applying Neighbor-Join and BioNJ algorithms to a matrix of pairwise distances estimated using a JTT model and then selecting the topology with superior log likelihood value. All positions with less than 95% site coverage were eliminated. The tree was annotated and visualized with the iToL web tool (itol.embl.de/) [58].

In the ATUMI genome, 49 GSTs were identified, 9 of which displayed isoforms (Fig. 7; Table 5). The number of genes in the ATUMI genome is very similar to what has been identified in TCAST, especially in the Delta, Epsilon, Sigma, and Theta classes. Relative to other insects, ATUMI and TCAST have expansions in the Epsilon, Sigma, Zeta, and Microsomal GST classes, which supports the hypothesis that these may be Coleoptera-specific class expansions [57]. The small number of genes in the Delta class for both ATUMI and TCAST suggests a class contraction or lack of expansion within the beetles.

**Table 5:** Comparison of the number of GSTs for ATUMI, AMELL, DMELA, and TCAST [57, 59]

GST class	ATUMI	AMELL	DMELA	TCAST
Delta	3	1	11	3
Epsilon	19	0	14	19
Omega	1	1	5	3
Sigma	7	4	1	7
Theta	1	1	4	1
Zeta	5	1	2	1
Microsomal	6	2	1	5
Unclassified	7	0	0	2
Total	49	10	38	41

Increases in the expression and activity Delta and Epsilon classes confer resistance to diverse classes of insecticides such organophosphates, organochlorines (DDT), and pyrethroids [54, 56]. These two GST classes tend to be the most numerous and dynamic in terms of expansions and contractions [57]. Therefore, it would appear that ATUMI possesses a wide diversity of GSTs, especially in the Epsilon class, to detoxify insecticides utilized for their control.

### Cytochrome P450

The cytochrome P450 monooxygenases (CYP450s) are classified as phase I metabolic enzymes that are involved in the biosynthesis, bioactivation, and regulation of endogenous compounds such as hormones, fatty acids, and sterols, as well as detoxification of xenobiotic compounds such as plant alleleochemicals and insecticides. Overexpression of CYP450s often underlies high levels of detoxification-mediated insecticide resistance in many insects [60–62]. In the 69 insect genomes that have been published, more than 7,500 P450 genes have been identified in 208 families across four clans (CYP2, CYP3, CYP4, and mitochondrial) [63].

**Figure 8:**
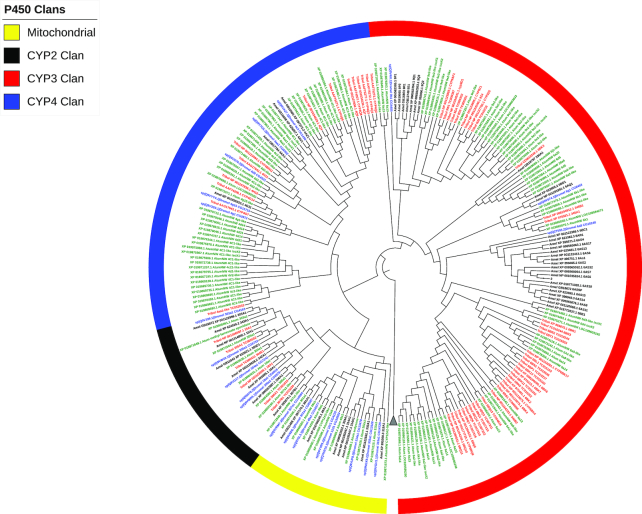
Maximum likelihood phylogenetic tree of the cytochrome P450 detoxification system. The bootstrap consensus tree inferred from 1,000 replicates is taken to represent the evolutionary history of the taxa *A. tumida* (ATUMI) in green, *A. mellifera* (AMELL) in black, *D. melanogaster* (DMELA) in blue, and *T. castaneum* (TCAST) in red, identified manually using the Uniprot and Pfam databases. Branches corresponding to partitions reproduced in less than 50% bootstrap replicates are collapsed. Initial tree(s) for the heuristic search was obtained automatically by applying Neighbor-Join and BioNJ algorithms to a matrix of pairwise distances estimated using a JTT model and then selecting the topology with superior log likelihood value. All positions with less than 95% site coverage were eliminated. P450s are clustered to CYP2, CYP3, CYP4, and mitochondrial clans. The tree was annotated and visualized with the iToL web tool (itol.embl.de/) [58].

In ATUMI, we found 116 genes across the four CYP clans (Fig. 8, Table 6). The CYP2 and mitochondrial clans contained 8 and 10 genes, respectively, and orthologs were identified in other species. The conservation in sequence and number is expected as many of the genes in these clans are involved in ecdysteroid biosynthesis [64]. In contrast to the conserved CYP2 and mitochondrial clans, there are clear expansions in CYP3 and CYP4 compared to other species. These expansions are typified by large expansions of a single family that lacks orthologs in other species [65]. Within the CYP3 clan, the 55 genes are clustered in smaller blooms, with the largest consisting of 13 genes. The 43 genes belonging to the CYP4 clan of ATUMI is among the largest seen in insects [7] with a noticeably large bloom of 20 genes. Additionally, CYPs in the CYP3 and CYP4 clans have been implicated in insecticide resistance [66–68]. Therefore, a rapid onset of insecticide resistance may be facilitated by the large number of CYPs in the CYP3 and CYP4 clans in the ATUMI genome.

**Table 6:** Comparison of CYP450 genes in ATUMI, AMELL, DMELA, and TCAST

P450 clan	ATUMI	AMELL	DMELA	TCAST
CYP2	8	8	6	8
CYP3	55	28	36	82
CYP4	43	4	32	49
Mitochondrial	10	6	11	10
Total	116	46	85	149

### Carboxyl/choline esterases

Carboxyl/choline esterases (COEs) are capable of metabolizing a wide variety of substrates, and their activity is involved in a number of physiological processes such as bioactivation of juvenile hormone and regulating acetylcholine interactions at the synapse [69, 70]. Increases in the amount of esterase expression and mutations in the catalytic site of esterases confer insecticide resistance [71, 72]. Insects possess a wide variety of COEs that are broadly classified as intracellular or dietary (clades A-C), secreted pheromone/hormone processing (clades D-G), and neurodevelopmental (clades H-M) [69].

**Figure 9:**
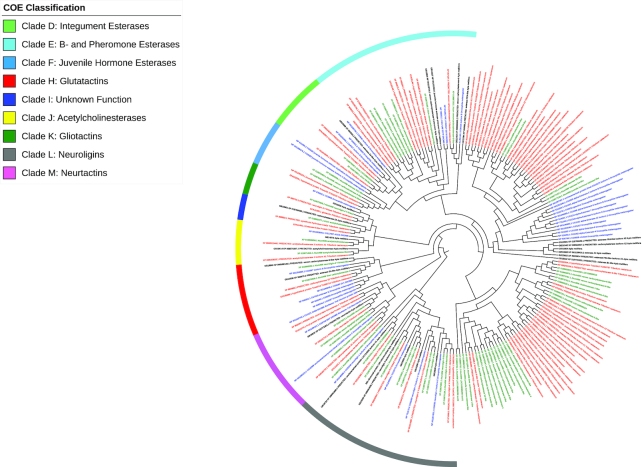
Maximum likelihood phylogenetic tree of carboxylesterase (COE) genes. The maximum likelihood bootstrap consensus tree (1,000 replicates) showing the relationships among COE genes from the genomes of *A. tumida* (ATUMI) in green, *A. mellifera* (AMELL) in black, *D. melanogaster* (DMELA) in blue, and *T. castaneum* (TCAST) in red, identified manually using the Uniprot and Pfam databases. Branches corresponding to partitions recovered in less than 50% of bootstrap replicates are collapsed. Starting tree(s) for the heuristic search was obtained automatically using neighbor-joining and BioNJ algorithms applied to a matrix of pairwise distances estimated using a JTT model and then selecting the topology with the superior log likelihood value. All positions with less than 95% site coverage were eliminated. The phylogenetically distinct clusters were named according to established nomenclature for COE genes [12]. The tree was annotated and visualized with the iToL web tool [24].

The ATUMI genome contained 60 genes encoding putative COEs, with only one displaying multiple isoforms (Fig. 9). The number of genes in the secreted and neurodevelopmental groups was mostly consistent with other insects (Table 7). The expansion of clade E (secreted β-esterase) is consistent with a similar expansion in TCAST. This expansion is not entirely characteristic of Coleoptera as DPOND and AGLAB only have four and one member of clade E, respectively [7]. The 10 genes for neuroligins is nearly twice the number seen in other insects [12, 59, 73]. Nevertheless, the general conservation in sequence and number suggests critical roles for these COEs across insects. In contrast to COEs in the secreted and neurodevelopmental groups, a vast majority of ATUMI COEs in the intracellular or dietary class lacked clear orthologs in TCAST, AMELL, or DMELA. This expansion of intracellular or dietary esterases is consistent with expansions observed in other insect genomes. These species-specific expansions of intracellular or dietary esterases may be due to dietary differences among these insects. Dietary esterases may also contribute to insecticide resistance [69]. Therefore, this expansive array of dietary esterases may allow ATUMI to detoxify insecticides that may be used for control.

**Table 7:** Comparison of COE from *Aethina tumida* to *Drosophila melanogaster, Tribolium castaneum*, and *Apis mellifera*.

COE subfamily	*ATUMI*	*AMELL*	*DMELA*	*TCAST*
Clades A-C (dietary)	27	8	13	55
Clade D (integument esterases)	2	1	3	5
Clade E (secreted β-esterase)	8	3	3	10
Clade F (JH esterases)	3	1	2	1
Clade H (glutactins)	2	0	4	2
Clade I (unknown function)	1	2	2	2
Clade J (acetylcholinesterases)	2	2	1	2
Clade K (gliotactin)	1	1	1	2
Clade L (neuroligins)	10	5	4	5
Clade M (neurotactins)	4	1	2	1
Total	60	24	35	85

Nomenclature and gene counts follow McKenna [12] and Claudianos [59]

ATUMI is an expanding invasive pest of honey bees, disrupting managed bee colonies and arguably having a strong impact on managed and naturally occurring colonies. We anticipate the resources described here will lead to novel methods to track and control this pest. The ATUMI genome also reveals numerous evolutionary distinctions relative to other sequenced arthropods. These distinctions help clarify the sensory cues used by ATUMI and the dietary habits of this beetle, and of beetles (order Coleoptera) more broadly.

## Methods

### DNA extraction

ATUMI adults were collected from a population maintained by the US Department of Agriculture-Agricultural Research Service (USDA-ARS) Honey Bee Breeding, Genetics and Physiology Laboratory (Baton Rouge, LA) in November 2011. ATUMI larvae were collected on 8 March 2014, from a continuous culture of small hive beetles maintained at the USDA-ARS Bee Research Laboratory. For adult beetles, extractions were carried out on three whole male beetles using the Qiagen DNAEasy kit. Larval DNA was extracted from 150 second-instar larvae in 30 groups of five larvae each. Larvae were crushed using a plastic pestle in 1 mL of freshly prepared cetyl trimethyl ammonium bromide (CTAB) buffer consisting of 100 mM TrisHCl (pH 8.0), 20 mM EDTA (pH. 8.0), 1.4 M NaCl, 2% CTAB, and 0.2% b-mercaptoethanol. The suspension was incubated at 65°C for 60 minutes, with gentle mixing at 0, 20, and 40 minutes. Samples were centrifuged for 2 minutes at 14k rpm (20,817 g) in an Eppendorf microcentrifuge. Next, 500 μL of the supernatant was moved using a wide-bore pipette into a sterile tube containing 500 μL chloroform:isoamylalcohol (24:1). After gentle mixing by hand, tubes were centrifuged at 14k rpm for 15 minutes. Approximately 400 μL of the aqueous layer was transferred into new tubes containing 250 μL cold isopropanol, followed by gentle mixing and incubation at 4°C for 30 minutes. Samples were centrifuged at 14k rpm for 30 minutes a 4°C, and then the supernatant was poured off. Pellets were washed with 1 mL cold 75% EtOH and centrifuged again for 2 minutes at 14k rpm. After the supernatant was poured off, the resulting pellets were washed in 1 mL cold 100% EtOH, centrifuged for 2 minutes, after which the EtOH was poured off, the pellets were spun for an additional 30 seconds, and the last of the wash was removed by pipette. Pellets were air-dried for 30 minutes, and the resulting DNA pellet was resuspended in 50 μL ddH_2_0. Samples were incubated for 30 minutes with 2.5 μL of an RNAse cocktail at 37°C, followed by gentle addition of 5 μL of 7M NaOAc and 100 μL EtOH. After 30 minutes of incubation on wet ice, the DNA samples were spun at 12k rpm for 30 minutes, washed once with 70% EtOH, and dried and suspended in 20 μL ddH_2_0. Extracts were pooled and assayed by gel electrophoresis to ensure DNA integrity and by Nanodrop (Thermofisher, Inc.) for quantification (180 ng/μL in 25 μL, 45 μg total DNA).

### DNA sequencing

In total, 1,173,425,522 Illumina DNA reads (101 bp per read with a 300 bp insert size, Hi-Seq 2500) were generated from 12 paired-end libraries generated from DNA from the three adult male beetles. An additional 1,235,055 Pacific Biosciences (PacBio) reads (average read length = 6,795 bp) were generated from 40 single-molecule real-time sequencing (SMRT)cells (Chemistry C2, PacBio, Menlo Park, CA), using DNA derived from the pooled larval beetles. A two-step method was used to assemble the genome. First, the Sparse assembler was used to build short but accurate contigs from the Illumina reads using the settings: (LD 0 K 41 g 15 NodeCovTh 1 EdgeCovTh 0 GS 600000000) [74]. The assembled contigs were used as a backbone for further assembly. Second, the PacBio reads were error corrected by the proovread package (default settings) [75], and the error-corrected PacBio reads were used to construct long contigs by filling the gaps of the backbones using the Sparc package deployed with default settings [76]. Genes were annotated using version 7.2 of the NCBI eukaryotic annotation pipeline [77]. Illumina mRNA paired-end sequencing reads (101 bp per read, >1000x transcriptome coverage) reflecting an equimolar pool of all ATUMI life stages (described in [78] and downloaded 11/2016 from USDA AgDataCommons [79]) were used to assist gene annotation. Full annotation details for this gene set are described in [80]. Transcriptome sequencing reads were aligned to the constructed ATUMI genome assembly to evaluate the completeness of the gene set using the TopHat2 package [48]. Reads were also mapped using HISAT2 [81], showing a marginal increase in aligned reads. We further assessed the completeness of the genome assembly using BUSCO [82]).

### Phylogenetic and genetic diversity of beetles

The official protein sets of ATUMI, the red flour beetle (*Tribolium castaneum*) [11], mountain pine beetle (*Dendroctonus ponderosae*) [13], Asian longhorned beetle (*Anoplophora glabripennis*) [12], dung beetle (*Onthophagus taurus*) [83], emerald ash borer (*Agrilus planipennis*) [84], coffee borer beetle (*Hypothenemus hampei*) [14], burying beetle (*Nicrophorus vespilloides*) [16], scarab beetle (*Oryctes borbonicus*) [15], honey bee (*Apis mellifera*) [85], and fruit fly (*Drosophila melanogaster*) [86] were used to query the BUSCO Endopterygota ortholog set. Single-copy orthologs shared by all 11 insect species were further used for phylogenetic analysis. Protein sequences of these orthologous groups were aligned using MUSCLE using default protein settings [87]. Alignments were quality trimmed with trimAI (-w 3 –gt 0.95 –st 0.01) [88], and the orthologous groups were concatenated for use in phylogenetic analysis. A maximum likelihood tree search was implemented using the program RAxML version 8.2.9 [89] with 1,000 bootstrap replicates (−N 1000 –m PROTGAMMAAUTO –f a). The final tree was viewed and edited with TreeGraph2 [90]. Microsatellite markers were identified in the ATUMI genome assembly using the Microsatellite Search and Building Database package and default settings [91]. The raw Illumina gDNA reads, used to assemble the ATUMI genome, were re-aligned to the assembly using BWA with default settings [92]. The aligned reads were used to identify SNP positions using GATK under default settings (version 3.6; [93]) and the further annotated with SNPEFF [94].

### Gustatory receptors

The repertoire of GRs has been preliminarily characterized for TCAST [95] (62 GRs) and *A. glabripennis* [96]. Additionally, online databases have listed gustatory receptors for *T. castaneum*, including UniProtKB [97, 98] and BeetleBase [99, 100]. Amino acid sequences for putative and identified *GR* genes were compiled from these resources and truncated to remove redundancies. The compiled TCAST gene set contained 71 *GR* genes. To identify and enumerate gustatory receptors for AGLAB and ATUMI, amino acid sequences of TCAST gustatory receptor genes were submitted to the ATUMI RefSeq gene set and genome assembly using BLASTP and TBLASTN, respectively. Putative *GR* genes for both species were selected from hits based on an E-score ≤ E^−100^. Using the dataset of *GR* genes compiled for *T. castaneum*, 38 and 11 putative GR proteins were identified for AGLAB and ATUMI, respectively. Sequences were aligned using MUSCLE [87]. The PhyML program (v3.1/3.0 aLRT) was used to build a phylogenetic tree using maximum likelihood method [28, 101]. The tree was further edited and visualized with the TreeDyn (v198.3) program [102]. All analyses from the sequence alignment to tree reconstruction were performed on the phylogeny.fr platform [103]. Sequences obtained in Newick format from this platform were used as input in the iTOL program to construct and visualize using an unrooted, circular phylogenetic tree [104].

### ABC transporters

Potential ATUMI ABC genes homologous to TCAST ABCs were identified using protein BLAST to search with each TCAST ABC sequence using WebApollo at [105]. Protein sequences from ATUMI, TCAST, and DMELA were then compiled and trimmed to exclude all but 51 residues around the Walker B motif of the nucleotide binding domain. This 51-amino acid sequence was then used to build the phylogenetic tree (see Table 3 for the sequences used from ATUMI). The maximum likelihood phylogenetic tree was constructed using the program MEGA, version 7 [106], using default parameters in all categories except the LG model of amino acid substitution with Gamma distributed substitution rates (based on Best Model determination within the MEGA program) and Partial Deletion treatment of gaps/missing data [107].

### Insecticide targets and detoxification genes

The predicted proteins from the official gene set of ATUMI (taxid 116153) were queried with TCAST orthologs for gene families and pathway members related to insecticide resistance via BLASTP. Putative orthologs in ATUMI were designated by >95% query coverage and E-value <1E^−100^.

## Availability of supporting data

Data supporting the results of this article are deposited at NCBI-Bioproject PRJNA256171. Further supporting data can also be found in the *GigaScience* respository, GigaDB [108].

## Additional files

**File S1 MS-Word:** Detailed material and methods.

**File S2 MS-Excel:** Orthology assignments for glycoside hydrolases (GHs) coded by ATUMI.

**File S3 MS-Excel:** Protein identifiers for orthogroup assignments.

## Abbreviations

ABC: ATP-binding cassette; Ace: acetylcholinesterase; Ach: acetylcholine; ATUMI: *Aethina tumida*; BLAST: Basic Local Alignment Search Tool; BUSCO: Benchmarking Universal Single-Copy Orthologs; COE: carboxyl/choline esterase; CTAB: cetyl trimethyl ammonium bromide; CYP450: cytochrome P450 monooxygenases; GCPR: G-protein-coupled receptor; GH: glycoside hydrolase; GR: gustatory receptor; GST: gluthatione-S-transferase; NCBI: National Center for Biotechnology Information; OP: organophosphate; SNP: single-nucleotide polymorphism; USDA-ARS: US Department of Agriculture-Agricultural Research Service

## Competing interests

The authors declare that they have no competing interests.

## Funding

Q.H. was supported by a competitive award (2017–06481) from the USDA National Institute of Food and Agriculture (J.D.E.)

## Author contributions

J.D.E. and Q.H. designed the study. Q.H. assembled the genome and led the bioinformatics analyses. All authors contributed to annotation and/or context, as well as writing the manuscript.

## Supplementary Material

GIGA-D-18-00144_Original_Submission.pdf

GIGA-D-18-00144_Revision_1_(2).pdf

GIGA-D-18-00144_Revision_2.pdf

Response_to_Reviewer_Comments_Original_Submission.pdf

Response_to_Reviewer_Comments_Revision_1.pdf

Reviewer_1_Report_(Original_Submission) -- Christopher Cunningham 5/30/2018 Reviewed

Reviewer_1_Report_Revision_1 -- Christopher Cunningham 9/16/2018 Reviewed

Reviewer_2_Report_(Original_Submission) -- Fei Li 6/5/2018 Reviewed

Reviewer_2_Report_Revision_1 -- Fei Li 9/16/2018 Reviewed

Supplemental Files
