# Supplementary material for: Genome of the small hive beetle (Aethina tumida, Coleoptera: Nitidulidae), a worldwide parasite of social bee colonies, provides insights into detoxification and herbivory
Source: Gigascience. 2018 Dec 7;7(12):giy138. doi: 10.1093/gigascience/giy138 (PMC6302959; doi:10.1093/gigascience/giy138)
Supplement: GIGA-D-18-00144_Original_Submission.pdf [file giy138_giga-d-18-00144_original_submission.pdf]

GigaScience

Genome of the small hive beetle (Aethina tumida, Coleoptera: Nitidulidae), a worldwide parasite of social bee colonies, provides insights into detoxification and herbivory

--Manuscript Draft--

**Manuscript Number:**	GIGA-D-18-00144
**Full Title:**	Genome of the small hive beetle (Aethina tumida, Coleoptera: Nitidulidae), a worldwide parasite of social bee colonies, provides insights into detoxification and herbivory
**Article Type:**	Research
**Funding Information:**	
**Abstract:**	Abstract<br>Background: The small hive beetle (Aethina tumida, ATUMI) is an invasive parasite of bee colonies. ATUMI feeds on both fruits and bee nest products, facilitating its spread and increasing its impact on honey bees and other pollinators. We have sequenced and annotated the ATUMI genome, providing the first genomic resources for this species and for the Nitidulidae, a beetle family that is closely related to the extraordinarily species-rich clade of beetles known as the Phytophaga. ATUMI thus provides a basal view of one of the most successful known animal groups.<br>Results: The ATUMI genome encodes fewer enzymes for plant digestion than the genomes of wood-feeding beetles, but nonetheless shows signs of broad metabolic plasticity. Gustatory receptors are few in number compared to other beetles, especially receptors with known sensitivity (in other beetles) to bitter substances. In contrast, several gene families implicated in detoxification of insecticides and adaptation to diverse dietary resources show increased copy numbers. The presence and diversity of homologs involved in detoxification differs substantially from the bee hosts of ATUMI.<br>Conclusions: Metabolic plasticity and a wide array of metabolic enzymes could allow ATUMI to exploit diverse food sources within bee colonies, including plant material, hive products, and bees themselves. A minimal set of gustatory receptors is consistent with the observation that, once a host colony is invaded, food resources are predictable. Our results provide new insights into the genomic basis for local adaption and invasiveness in ATUMI, and a blueprint for control strategies that target this pest without harming their honey bee hosts.
**Corresponding Author:**	Jay D Evans, PhD<br>USDA Agricultural Research Service<br>Beltsville, MD UNITED STATES
**Corresponding Author Secondary Information:**	
**Corresponding Author's Institution:**	USDA Agricultural Research Service
**Corresponding Author's Secondary Institution:**	
**First Author:**	Jay D Evans, PhD
**First Author Secondary Information:**	
**Order of Authors:**	Jay D Evans, PhD
	Duane D McKenna, PhD
	Erin D Scully
	Steven C Cook
	Benjamin Dainat
	Noble Egekwu
	Nathaniel Grubbs
	Dawn Dawn Lopez

Powered by Editorial Manager® and ProduXion Manager® from Aries Systems Corporation

	Marce D Lorenzen
	Steven M Reyna
	Frank D Rinkevich
	Peter Neumann
	Qiang Huang
**Order of Authors Secondary Information:**	
**Additional Information:**	
**Question**	**Response**
Are you submitting this manuscript to a special series or article collection?	No
**Experimental design and statistics**<br><br>Full details of the experimental design and statistical methods used should be given in the Methods section, as detailed in our Minimum Standards Reporting Checklist. Information essential to interpreting the data presented should be made available in the figure legends.<br><br>Have you included all the information requested in your manuscript?	Yes
**Resources**<br><br>A description of all resources used, including antibodies, cell lines, animals and software tools, with enough information to allow them to be uniquely identified, should be included in the Methods section. Authors are strongly encouraged to cite Research Resource Identifiers (RRIDs) for antibodies, model organisms and tools, where possible.<br><br>Have you included the information requested as detailed in our Minimum Standards Reporting Checklist?	Yes
**Availability of data and materials**<br><br>All datasets and code on which the conclusions of the paper rely must be either included in your submission or deposited in publicly available repositories (where available and ethically appropriate), referencing such data using a unique identifier in the references and in the "Availability of Data and Materials" section of your manuscript.	Yes

Have you have met the above requirement as detailed in our Minimum Standards Reporting Checklist?	

Powered by Editorial Manager® and ProduXion Manager® from Aries Systems Corporation

Main manuscript Click here to download Manuscript EvansetaalSHBsubmit.pdf

Genome of the small hive beetle (*Aethina tumida*, Coleoptera: Nitidulidae), a worldwide parasite of social bee colonies, provides insights into detoxification and herbivory

Jay D. Evans[1*], Duane McKenna[2], Erin Scully[3], Steven C. Cook[1], Benjamin Dainat[4], Noble Egekwu[1], Nathaniel Grubbs[5], Dawn Lopez[1], Marcé D. Lorenzen[5], Steven M. Reyna[5], Frank D. Rinkevich[6], Peter Neumann[7], Qiang Huang[7*]

[1]USDA-ARS, Bee Research Laboratory, BARC-East Building 306, Beltsville, Maryland 20705, USA

[2]Department of Biological Sciences, University of Memphis, 3700 Walker Ave., Memphis, TN 38152, USA

[3]USDA-ARS, Center for Grain and Animal Health, Stored Product Insect and Engineering Research Unit, Manhattan, KS 66502, USA

[4]Agroscope, Swiss Bee Research Center, CH-3003 Bern, Switzerland

[5]Department of Entomology and Plant Pathology, North Carolina State University, 1566 Thomas Hall, Raleigh, NC 27695, USA

[6]USDA, Honey Bee Breeding, Genetics and Physiology Laboratory, 1157 Ben Hur Road, Baton Rouge, LA 70820, USA

[7]Institute of Bee Health, Vetsuisse Faculty, University of Bern, Schwarzenburgstrasse 161, CH-3097, Liebefeld, Switzerland

[*] For correspondence

Email: Jay.Evans@ARS.USDA.GOV (JDE); qiang-huang@live.com (QH)

Abstract

Background: The small hive beetle (*Aethina tumida,* ATUMI) is an invasive parasite of bee colonies. ATUMI feeds on both fruits and bee nest products, facilitating its spread and increasing its impact on honey bees and other pollinators. We have sequenced and annotated the ATUMI genome, providing the first genomic resources for this species and for the Nitidulidae, a beetle family that is closely related to the extraordinarily species-rich clade of beetles known as the Phytophaga. ATUMI thus provides a basal view of one of the most successful known animal groups.

Results: The ATUMI genome encodes fewer enzymes for plant digestion than the genomes of wood-feeding beetles, but nonetheless shows signs of broad metabolic plasticity. Gustatory receptors are few in number compared to other beetles, especially receptors with known sensitivity (in other beetles) to bitter substances. In contrast, several gene families implicated in detoxification of insecticides and adaptation to diverse dietary resources show increased copy numbers. The presence and diversity of homologs involved in detoxification differs substantially from the bee hosts of ATUMI.

Conclusions: Metabolic plasticity and a wide array of metabolic enzymes could allow ATUMI to exploit diverse food sources within bee colonies, including plant material, hive products, and bees themselves. A minimal set of gustatory receptors is consistent with the observation that, once a host colony is invaded, food resources are predictable. Our results provide new insights into the genomic basis for local adaption and invasiveness in ATUMI, and a blueprint for control strategies that target this pest without harming their honey bee hosts.

Keywords: Coleoptera, pollination, *Apis mellifera*, invasive pest, phytophagy, invasive species, glycoside hydrolase.

Introduction

The small hive beetle (= ATUMI; *Aethina tumida* Coleoptera: Nitidulidae, Murray, 1867) is a rapidly spreading invasive species originating from sub-Saharan Africa. ATUMI is now found on all continents except Antarctica [1-4]. Outside of its endemic range, it has become an economically important parasite of social bee colonies, including honey bees, bumblebees and stingless bees [2] (Figure 1). ATUMI significantly impacts beekeeping and the regulation of honey bees and hive products worldwide. ATUMI pupate in the soil then emerge as adults to infest social bee nests [2]. Once inside the bee nest, adult ATUMI employ a “sit-and-wait” strategy, relying on the resources of the nest for nutrition and shelter until options for successful reproduction arise [2]. ATUMI eggs are laid within colonies and developing larvae feed until they leave the colony for pupation in nearby soil [2]. ATUMI larvae and adults can feed on a large variety of food sources inside and outside of social bee colonies, including fruits, meat, adult bees, bee brood and bee food stores (pollen and honey) [1, 5, 6]. Beetles and their bee hosts show elaborate behaviours. For example, honey bees attempt to confine adult ATUMI to prisons built from plant resins [6] and beetles can also manipulate guard bees to obtain food by rubbing their antennae against the guarding bees’ mandibles, inducing them to regurgitate food.

ATUMI belongs to the beetle family Nitidulidae (sap beetles; c. 4,500 species), which feed mainly on decaying vegetable matter, over-ripe fruit, or sap. The Nitidulidae belong to the superfamily Cucujoidea (sap, bark and fungus beetles), which is either the sister-group of the Phytophaga (leaf beetles, weevils, longhorned beetles and their relatives; [7], the most species rich radiation of plant-feeding animals on Earth with >125,000 described species), or forms a paraphyletic clade subtending the Phytophaga [8]. In the latter case, the Phytophaga are derived from within Cucujoidea. Interestingly, the trophic habits of Nitidulidae may therefore represent a transitional stage from fungivory, saprophagy, and detritivory (the typical habit(s) of most Cucujoidea and its containing clade, series Cucujiformia) to phytophagy (feeding on plants), the typical trophic habit of Phytophaga. Comparative studies of the ATUMI genome may therefore provide new insights into the evolution and genomic basis of phytophagy in beetles.

To date, just 10 beetle genome assemblies have been released [9], of which only 7 are published, despite there being >400,000 described beetle species. These are: *Tribolium castaneum* (red flour beetle, TCAST; Tenebrionoidea: Tenebrionidae: Tenebrioninae; [10], *Anoplophora glabripennis* (Asian longhorned beetle, AGLAB; Chrysomeloidea: Cerambycidae: Lamiinae; [11]), *Dendroctonus ponderosae* (mountain pine beetle, DPOND; Curculionoidea: Curculionidae: Scolytinae; [12]), *Hypothenemus hampei* (coffee berry borer beetle, HHAMP; Curculionoidea: Curculionidae: Scolytinae; [13]), *Oryctes borbonicus* (Reunion Island scarab beetle, OBORB; Scarabaeoidea: Scarabaeidae: Dynastinae; [14]), *Onthophagus taurus* (bull headed dung beetle, OTAUR; Scarabaeoidea: Scarabaeidae: Scarabaeinae; Unpublished), *Nicrophorus vespilloides* (burying beetle, NVESP; Staphylinoidea: Silphidae: Silphinae; [15]),

Agrilus planipennis (emerald ash borer, APLAN; Buprestoidea: Buprestidae: Agrilinae; Unpublished), *Leptinotarsa decemlineata* (Colorado potato beetle, LDECE; Chrysomelidae: Chrysomelinae: Doryphorini; [16]), and *Pogonus chalceus* (salt marsh beetle, PCHAL; Carabidae: Trechinae: Pogonini; Unpublished). The ATUMI genome described here joins this group as the only representative from the superfamily Cucujoidea.

Along with removing resources from honey bee colonies ATUMI plays an important role in the colony microbiome. It has been reported that ATUMI is a vector of three honey bee pathogens; Deformed wing virus (DWV) [17], sacbrood virus (SBV) [17], and *Paenibacillus larvae*, the causal agent of American foulbrood disease (AFB)[18]. ATUMI appears to acquire viral and bacterial pathogens through feeding. This could be through trophallaxis (oral feeding by regurgitation) with infected bees, feeding on infected workers or bee brood, or by feeding on contaminated hive components (i.e. wax, pollen or honey) [17].

Though the associations of ATUMI and bee pathogens need further study, the association between ATUMI and the yeast *Kodamaea ohmeri* has been well established [19-21]. *K. ohmeri* was first reported by Torto et al. [20] as a fungal associate of ATUMI. This fungus is the causal agent of the fermentation or slime associated with ATUMI [22]. Stored pollen becomes inoculated with the yeast when adult beetles invade a hive, and the resulting fermentation produces volatiles that mimic the honey bee alarm pheromone. These volatiles act as an aggregation kairomone for the beetles, signaling the presence of nutritional resources {Torto, 2007 #396;Torto, 2007 #314; Torto, 2014 #395}. *K. ohmeri* can be found topically on adults and larvae, as well as their frass [22, 23]. *K. ohmeri* has also been found in bumble bee hives that have been infested by ATUMI [24]. Though ATUMI uses *K. ohmeri* as a way to identify

suitable hives, the interaction between the beetle and yeast can be used against the beetle. In recent years, scientists have proposed traps utilizing *K. ohmeri* as an attractant for ATUMI [25, 26].

A robust reference genome assembly comprised of 343.3 million base pairs was used to identify and annotate 14,076 protein coding genes, over 3000 additional transcribed features and a strong complement of repetitive DNA's, tRNA's, and transposable elements. The described protein-coding genes provide strong candidates for core metabolism and development, and suggest that these beetles, like their honey bee hosts, rely on olfactory cues and less so on chemosenses related to taste. An analysis of protein groups involved in insecticide metabolism reveals a large repertoire of detoxification enzymes to mediate xenobiotic interactions. The described resources will be useful for both chemical and non-chemical approaches for controlling this key pest of honey bees.

Data Description

DNA was extracted from both larval and adult ATUMI. In total, 1,173,425,522 Illumina DNA reads (101 base-pairs [bp] per read with a 300 bp insert size, Hi-Seq 2500) were generated from 12 paired-end (PE) libraries generated from three adult male beetles. Additionally 1,235,055 Pacific BioSciences (PacBio) reads (average read length = 6795 bp) were generated from 40 SMRT cells (Chemistry C2, PacBio, Menlo Park, CA), using DNA derived from a pooled sample of larval beetles. A two-step method was used to assemble the genome. First, the Sparse assembler was used to build short but accurate contigs from the Illumina reads [27]. The assembled contigs were used as a backbone for further assembly. Second, the PacBio reads

were error corrected by the proovread package [28] and the error-corrected PacBio reads were used to construct long contigs by filling the gaps of the backbones using Sparc package [29]. The final genome assembly is deposited in Genbank as https://www.ncbi.nlm.nih.gov/assembly/GCF_001937115.1/. Genes were annotated using the NCBI eukaryotic annotation pipeline, resulting in a consensus gene set (https://www.ncbi.nlm.nih.gov/genome/annotation_euk/Aethina_tumida/100/). Illumina mRNA paired-end sequencing reads (101 bp per read, >1000x transcriptome coverage) reflecting an equimolar pool of all ATUMI life stages (described in [30] and downloaded from USDA AgDataCommons; https://tinyurl.com/ybanauxb) were used to assist gene annotation. Those mRNA sequencing reads were also aligned to the constructed ATUMI genome assembly to evaluate the completeness of the gene set, using the TopHat2 package [31]. We further assessed the completeness of the genome assembly using BUSCO (Benchmarking Universal Single Copy Orthologs; [32]).

Results and discussion

Genome traits, genetic diversity and phylogenetic analysis

We generated a genome assembly of 343Mbp (Million base pair) comprised of 3063 contigs (contig N_{50}=298kb; Table 1). The genome sizes of sequenced and assembled beetle species vary greatly from 160Mbp to 1.1Gbp. The size of the ATUMI genome assembly is similar to that of the red flour beetle (165.9 Mbp), but much smaller than the more derived Asian longhorn beetle (707.7 Mbp). A total of 1,293,015 heterozygous single nucleotide polymorphism (SNP) positions were identified, with an average density of one SNP per 181 bp. SNP density was

significantly different across contigs ($P < 0.01$). This pattern was not related to contig size. Overall, 60.2% of SNPs occurred on contigs with annotated genome features and 22.5% were within gene regions.

The NCBI eukaryotic genome annotation pipeline proposed 14,076 protein-coding genes and a total of 17,436 mRNA models. When our previous RNA sequencing reads were aligned to the genome assembly alongside the predicted gene models, 99.63% of the predicted mRNA models and 99.56% of the predicted protein-coding genes were supported. It is possible that the 64 protein-coding genes undetected by RNA-Seq were not expressed, expressed too briefly, or not captured in our pooled RNA samples. Alternatively, these might reflect partial or inaccurate gene models or pseudo-genes that are no longer functional in this beetle.

By aligning the ATUMI official protein set against 2444 core Endopterygota Benchmarking Universal Single-Copy Orthologs (BUSCO), 97.5% of complete BUSCOs were found (Figure 2b). We further aligned the ATUMI genome assembly against Endopterygota set of BUSCOs and 92.8% of complete BUSCOs were found (Supplemental File 1). The results suggest a high level of completeness in the genome assembly, as well as the official set of gene models. By comparing single-copy orthologs among the sequenced beetles (ATUMI, TCAST, DPOND, AGLAB, ATAUR, APLAN, HHAMP, NVESP), honey bees (AMELL) and *Drosophila melanogaster* (DMELA), 181 shared ortholog groups were found. A phylogenetic tree was built by concatenating these shared 181 orthologous groups (Figure 2a). These results suggest that ATUMI is sister to TCAST and the Asian longhorned beetle (AGLAB). OrthoDB [33] orthology delineation revealed that ATUMI has 7066 conserved orthologous groups with beetles and 4554 orthologous groups shared with ten additional insect species.

Loss and duplication of core genes from small hive beetle genome

BUSCO data sets represent a core set of conserved genes. The duplication and absence of such core genes could represent important evolutionary changes in species or in lineages, as their occurrence can be variable across the insects (Figure 3). A complete protein set of 11 insect species was used for BUSCO alignment. We found 337 core Endopterygota BUSCOs that were either fragmented or completely lost from at least two predicted beetle gene sets, suggesting they were lost from the genomes of two or more species or were not identified due to incomplete gene sets. We mapped the common ancestor sequences of these 337 missing orthologs and 2442 Endopterygota BUSCOs to the Pfam database. Among the 'lost' orthologs, 1094 protein domains were found. Among 2442 Endopterygota orthologs, 4632 protein domains were found. By comparing the count distribution of each domain between lost orthologs and overall orthologs, no significant difference was found (χ^2=8.9, df=27, P<0.05). Among the lost orthologs, a methyltransferase (MT), a glycosyltransferase (GT), and two proteins with beta-transducin repeats (WD) and zinc finger (ZF) domains, respectively showed the highest counts and were also absent from at least four beetle species (Figure 2c). When APLAN was treated as the nearest common ancestor, the pattern of gene loss significantly deviated from random (P > 0.05). Specifically, genes missing from basal members of clades tended to be absent thereafter in that clade. For example, *methyltransferase* was lost in ATUMI, DPOND, AGLAB and HHAMP, but is present in TCAST.

Glycoside hydrolases

Glycoside hydrolases (GHs) are important enzymes that aid in the digestion of plant cell walls and carbohydrates in insects [34]; however, GHs can also contribute to additional physiological processes, including remodeling of the peritrophic matrix (PM) [35], lysosomal enzyme activity, glycoprotein oligosaccharide catabolism, immune response, and growth and development [36, 37]. Despite the recalcitrance and variation of the diet of ATUMI, a limited diversity of GH families was identified in the genome compared to other beetle genomes. While phytophagous insects, such as AGLAB [7], DPOND [12] and HHAMP [13] harbored anywhere from 19-24 different GH families represented by 101-199 genes, only 14 GH families represented by 91 genes were identified in the ATUMI genome. Only OBORB, whose diet is unknown [14], had a lower GH family diversity and GH copy number, with 13 different families represented by 47 different genes identified. No GH families unique to ATUMI were identified (Supplemental Files 2 and 3).

The most prominent GH families in the ATUMI genome were GH 18 (20 copies), GH 31 (11 copies), and GH 38 (10 copies; Figure 4). While the majority of the GH 18 and GH 38 genes had highest scoring BLASTP matches to chitinases likely linked to PM remodeling and lysosomal α-mannosidases, respectively, all of the GH 31 genes had matches to uncharacterized α-glucosidases (four of which contained signal peptides for secretion) [38] whose substrate preferences could not be inferred with homology or domain searches. Despite their prominence in the ATUMI genome, copy numbers of GH 18 and 31 genes were similar to those found in the genome of other beetles. In contrast, GH 38 copy numbers were high in ATUMI and were exceeded only by TCAST.

Using orthology searches, five orthogroups containing GHs were more prominent in the ATUMI genome compared to other beetles. These contained genes with highest scoring BLASTP matches to GH 30 glucosylceramidase (eight copies), uncharacterized GH 31 α-glucosidases (five copies; mentioned above), GH 16 β-1,3-glucan binding protein (five copies), GH 38 lysosomal α-mannosidase (five copies; mentioned above), and GH 18 chitinase (three copies; mentioned above). While the chitinases and β-1,3-glucan binding proteins are likely involved in PM or exoskeleton remodeling, the glucosylceramidases are likely involved in breaking down sphingolipids. Additionally, two GH genes encoded by the ATUMI genome, which lacked orthology to other beetle GHs, included a GH 2 family gene coding for β-mannosidase and a GH 35 family gene coding for β-galactosidase. While the β-mannosidase gene is likely associated with lysosomal degradation of N-linked glycoproteins, the GH 35 β-galactosidase contains a signal peptide and may be linked to digestive processes. Other beetles code for GH 2 β-mannosidases and GH 35 β-galactosidases, so it is unclear why these two genes were not assigned to orthogroups. However, these genes are present in multiple copies in almost all beetle genomes. Thus, their evolutionary history is complex and it may be difficult to assign orthologs in some cases.

Overall, ATUMI lacked a diverse and expansive repertoire of GHs relative to phytophagous beetles, which may be directly linked with its diet. Pollen generally contains high concentrations of the monosaccharides, glucose and fructose [39], which can be used directly for ATP production by the glycolysis pathway (glucose) or after phosphorylation by fructokinase (fructose). Therefore, although pollen can also contain starch, sucrose, and small amounts of pectin [39], digestion of more complex carbohydrates may not be necessary, requiring a less

expansive repertoire of GH enzymes relative to phytophagous beetles. Supporting this hypothesis, genes coding for enzymes capable of digesting starch were identified (α-amylase), but genes coding for invertases and polygalacturonases for sucrose and pectin digestion could not be identified. Alternatively, microbial symbionts harbored by ATUMI may facilitate the breakdown of these polysaccharides as has been observed previously in AMELL [40].

Gustatory Receptors

G-protein-coupled receptors (GPCRs) comprise a large family of integral membrane proteins found in cells of all eukaryotes [41]. GPCRs function to detect extracellular stimuli, and activate cellular signal transduction pathways that ultimately lead to physiological and behavioral responses. GPCR structure includes seven trans-membrane helices, two highly conserved cysteine residues that form protein-stabilizing disulfide bonds in the extracellular regions, a G-protein activation site, and a ligand binding site(s) [42]. The GPCR is activated when a stimulus (ligand) binds to a specific site on the protein, thus triggering a conformational change in its 3-dimensional structure. This, in turn, activates an intracellular coupled G-protein, which then interacts with other signaling molecules (e.g., cAMP) or other receptors (e.g., voltage-gated channels of neuronal cells) that affect downstream physiological processes of the cell. Different GPCRs detect a wide range of different stimuli (e.g., photons, neurotransmitters, pheromones/hormones, and tastants), which in turn result in equally broad physiological and behavioral responses (e.g., phototaxis, locomotion, mating, and nutrient acquisition). In many instances, the ability of an organism to respond appropriately to such stimuli is key to their adaptability and survival.

Gustatory receptors (Grs) are important components of an organism's sensory machinery; the ability of animals to distinguish between nutritious, noxious, and possibly toxic compounds is a matter of life or death. Insect Grs belong to novel arthropod GPCR gene superfamilies, which are phylogenetically unrelated to mammalian taste receptor genes, and distinct from related insect odorant/pheromone receptor genes [43]. The Grs are associated with specialized sensory structures (e.g., pits or setae), which are located on diverse regions of the insect body, including tarsi, antennae, mouthparts, and even internally. At these sites, Grs are located on the dendritic ends of gustatory neurons, and have affinities for specific types of ligands. In insect Grs, downstream signaling cascades can proceed in either a G-protein dependent or independent manner [44, 45]. The effectiveness of the resulting signaling cascade is dependent on the specificity of differential binding of ligands to the binding site of the protein's extracellular domain. The system has been honed over evolutionary time, and has given rise to receptors binding either sweet (attractive) or bitter (aversive) tastants, [46, 47]. An amino-acid substitution in a ligand-binding region may affect the range at which different ligand's receptors may bind, particularly for Grs perceiving sugars [48].

Stemming from their importance to insect biology, GPCRs have been characterized from genomic and transcriptomic studies for a number of economically important insects, or those having an ecological and/or epidemiological significance, including TCAST [10], AGLAB [11] and now ATUMI (this study). Understanding the chemosensory abilities of insects, particularly pest insects, is important for designing possible means of control that target the insect's ability to find and/or distinguish among nutrients or to detect poisons, and/or developing baits containing insecticides formulated with highly attractive substances. ATUMI is an economically

important pest of AMELL colonies, and in some regions of the world, poses a significant threat to honey bee colony health and the vitality of beekeeping industries. Even though food sources outside of social bee colonies may play a role [5], field data so far [49]suggest that bee colonies are the key factor for ATUMI adult maintenance and successful reproduction. To gain insights into the molecular sensory repertoire available to ATUMI for distinguishing between different tastants, here we provide a preliminary analysis of the gustatory receptor gene (*Gr*) repertoire of ATUMI.

General features of the cladogram include *Gr* genes grouping into four main clades that correspond with perception of different tastants (sweet or bitter; Figure 5). Designations of the type of substance perceived by the receptors come from literature reports for other taxa (e.g., *Drosophila* sp.), and from databases (above), or from location of uncharacterized proteins within the cladogram. A group of apparently highly conserved genes encoding proteins for perceiving sweet substances (clades *5a* and *64a-f*) is separate from other groups that show higher sequence variability; a pattern seen in other studies (e.g., [50]). Proteins of *Gr5a* and *Gr64a-f* can form heterodimeric complexes at receptor sites, and may or may not be necessary together for perception of different sugars [51, 52]. ATUMI appears to lack a *Gr5a* gene (Table 2; Figure 5), suggesting this gene may not be necessary for perceiving sweet tastants. In this group of ATUMI *Gr* genes, it is interesting to note that one candidate with a very long branch-length (XP_019866072) encodes a 379 amino-acid protein derived from 3 exons, and has a very long intron. It is unclear why this gene is so distinct compared to the relatively highly conserved sequences for other related *Gr* genes.

A major finding is that ATUMI has a substantially depauperate repertoire of *Gr* genes

compared to both AGLAB and TCAST. Based on the cladogram, ATUMI is represented in each of the major gene lineages (Figure 5). Thus, the low number of *Gr* genes in ATUMI is more likely the result of a lack of gene expansion in particular lineages or subfamilies of *Gr* genes rather than gene loss. A similarly small number of *Grs* is evident in the honey bee genome [53]. In that species, the relatively reduced *Gr* gene repertoire may be a consequence of restricted dietary breadth (specialist on pollen and nectar), and also possibly arises from the processing of collected foods by adult workers and microbes, which may reduce the load of plant secondary compounds. AMELL larvae are fed processed foods by attending nurse bees, so they may not need an expansive repertoire of Grs to discriminate among different tastants [50]. Because of the close affinity of ATUMI with honey bees, including sharing a similar diet, the evolutionary pressures limiting expansion of *Gr* genes in ATUMI may be similar. As an example, TCAST, a dietary generalist, shows a significant expansion in the *Gr28a/b* gene complex (Table 2); genes in this complex may be important for perceiving plant secondary compounds [54]. A similar reason may explain expansion of the *Gr2a* gene subfamily observed for both TCAST and AGLAB, although AGLAB has a more specialized diet than TCAST. Interestingly, honey bees have a greatly expanded repertoire of odorant receptor (*Or*) genes [50]; a full examination of the *Or* gene repertoire of ATUMI should be undertaken to determine whether a similar expansion is observed for ATUMI.

Voltage-gated sodium channel

The voltage-gated sodium channel (Na_{v1}) is responsible for generating action potentials in neurons. Functional channels typically consist of an α-subunit that is responsible for the flow of

ions across the membrane and auxiliary subunits (i.e. tipE, TEH1-4, or β-subunits) that modify channel gating and kinetics. The α-subunit is comprised of four repetitive domains (I-IV) that contain six transmembrane segments (S1-6). Of these transmembrane segments, S1-4 act as voltage sensors, especially S4 which contains many positively charged residues. The pore is formed by S5-6 and associated re-entrant loops [55]. Pyrethroids and DDT act on the Na_{v1} channel by maintaining the open state of the channel via interactions with two proposed binding sites [56, 57]. A diverse collection of mutations in Na_{v1} has been identified in many populations of pyrethroid-resistant pests and neurophysiological studies of heterologously-expressed channels have confirmed the role of these mutations in pyrethroid resistance [58].

The annotated ATUMI sodium channel contains an open reading frame of 6147 bases (XM_020010801.1) that encodes a 2048 amino acid protein (XP_019866360.1). Only a single transcript and protein were predicted from the ATUMI assembly, however, Na_{v1} is known to possess optional and alternative exons in some insects [59-61]. Interestingly, the optional exons a, b, and j were identified in the predicted ATUMI protein, while optional exons e, f, h, and i were not. Two pairs of alternative exons c/d and k/l were previously identified. Of these pairs, the predicted protein contained alternative exons c and l. This predicted inclusion of exons c and l is not likely representative of actual exon use, as exons d and k are the most commonly included exons in many species at many life stages [59-63]. Further cloning experiments to determine the actual optional and alternative exon use in ATUMI Na_{v1} should be informative.

A large number of mutations in Na_{v1} have been associated with target site resistance to pyrethroids and DDT [58]. We did not identify such mutations in ATUMI nor is this species known to be resistant to these insecticides. Therefore, this sequence serves as a reference for a

susceptible target site for pyrethroids and DDT. GardStar® (Y-Tex, Cody WY) contains the pyrethroid permethrin and is labeled for use as a soil drench around honey bee colonies to kill wandering larvae and pupae. Although not currently labeled for ATUMI control, tau-fluvalinate (sold as Apistan®) also is effective at killing ATUMI [64, 65]. Should these products be used more frequently to control ATUMI, they will undoubtedly select for resistant mutations in the sodium channel as has occurred for the honey bee parasitic mite, *Varroa destructor* [66].

Acetylcholinesterase

Acetylcholinesterase (Ace) cleaves acetylcholine (ACh) to regulate the effect of the neurotransmitter in the synaptic cleft. In insects, Ace is the target of organophosphate (OP) and carbamate insecticides and mutations in Ace result in target-site insensitivity to these two insecticide classes [67, 68].

ATUMI is predicted to possess active forms of both Ace1 (XP_019871456.1) and Ace2 (XP_019866656.1) and these proteins share high identity to the respective Ace orthologs in other insects (Supplemental File 2). Ace mutations involved in OP resistance [68, 69] are found to be in the susceptible state in the predicted Ace proteins of ATUMI (Table 3). In the cases where an alternative amino acid was found in ATUMI (i.e, ATUMI_Ace2 position 198), that same amino acid was seen in other insects that were presumably sensitive to OPs, so it does not likely confer resistance.

Ace2 performs primary acetylcholine esterase activity in honey bees, while Ace1 is the primary enzyme in beetles and most other insects [70]. Therefore identifying compounds that only inhibit ATUMI_Ace1 may provide a level of ATUMI-specific control. CheckMite+™ (Bayer

Health Care LLC, Shawnee Mission, KS) contains the organophosphate, coumaphos, and is currently labeled for in-hive use to control *V. destructor* and ATUMI [64, 71]. *Varroa destructor* has been shown to be resistant to coumaphos, due to metabolic adaptations to esterases [72, 73].

ATP-Binding Cassette Proteins

ATP-binding cassette (ABC) proteins are a large, diverse family of proteins found in most organisms, from bacteria to plants and vertebrates. The name is derived from the protein domains used to bind and hydrolyze ATP, a function most ABC proteins use to engage in active transport of molecules across cell membranes. As such, most of these proteins are comprised of two types of domains: the nucleotide-binding domain (NBD), and the transmembrane domain (TMD). To function as transporters, two NBDs and two TMDs are required, so several ABC transporters, known as full transporters, possess two of each. However, many ABC transporters only possess a single NBD and a single TMD; known as half transporters, these proteins function in homo- or heterodimers to transport target substances. ABC proteins can be separated into eight subfamilies, designated A-H, based on differences in NBD sequences.

This family of transporters is perhaps most notable for moving toxins into or out of cells, which has resulted in the identification of several of these proteins playing a role in the resistance of cancer cells to multiple drug treatments (Multi-Drug Resistant, MDR). So it is not surprising that some of these proteins have been identified as having roles in insect susceptibility or resistance to certain insecticides (Reviewed by {Dermauw, 2014 #70). In spite of their importance for shaping pest control methods, these genes are under-studied in insects, with few having been fully characterized in any species. The status of ATUMI as a pest of bee hives makes it important to understand what role ABC genes may play in how beekeepers control this species.

The beetle genetic model organism, TCAST, has had its full suite of ABC-family genes identified through a combination of RNA-seq and genomic analysis. In this species, 74 genes

have been identified (Table 4; {Broehan, 2013 #71;Grubbs, 2015 #72}). The translation products of these genes were used to query the ATUMI genome, in which 56 ABC genes were identified (Table 4). In most respects, the makeup of ABC genes in ATUMI resemble those found in TCAST - both species have identical numbers of ABC-B, D, E, F, and H subfamily members. Indeed, the numbers of members in the D-F and H subfamilies are highly conserved, with DMELA having the same number, and clear one-to-one relationships can be seen in these subfamilies among the members from each species (Figure 6). It should be noted that members of subfamilies E and F do not function as transporters, and are highly conserved, including in number, between insects and humans. Moreover, RNAi targeting ABC-E and one of the ABC-F genes in TCAST resulted in complete mortality, suggesting that the cellular roles of these genes may also be conserved. The ABC-B subfamily also appears well conserved, and may be worth further scrutiny in ATUMI, since this subfamily has been associated with resistance to several classes of pesticides in multiple species [74].

ATUMI differed from TCAST in member counts for three ABC subfamilies (Table 4). The first was subfamily A, for which only four members could be identified in ATUMI, relative to the ten found in TCAST and DMELA, or even the nine found in some other insect species . However, it is important to note that ABC-A genes are fairly large full transporters, and as such are often complex and difficult to identify in full. So, it is likely that some of the ABC-A genes are either not present in the current genome assembly, or are too fractured to recognize. It is also interesting to note that the beetle ABC-A genes appear to segregate from those of DMELA (Figure 6), suggesting possible pesticide targets against ATUMI*,* which may not harm other species, including pollinators.

TCAST appears to have one more ABC-G gene than does ATUMI; specifically, ATUMI appears to lack an ortholog of the well-studied DMELA eye-pigment transporter known as Brown (Bw). Eye color in DMELA is determined by two pigment families, ommochromes and pteridines, both of which require the ABC transporter, White, which dimerizes with Scarlet to import ommochromes, or Bw to import pteridines. However, many other insect species only use ommochromes to pigment eyes [75], so when initial work on the ABC genes in TCAST failed to identify an ortholog to Bw, researchers concluded that since the protein was not required for eye pigmentation, it had been lost in TCAST. However, later work was able to identify an ortholog by focusing on only the most conserved portions of a protein that had otherwise substantially diverged [75]. It is possible that similar divergence has also prevented clear identification of a Bw ortholog in ATUMI. Otherwise, most other ABC-G genes have clear one-to-one orthologs in all three species (Figure 6).

The largest subfamily, the ABC-C genes, is known to play roles in multi-drug resistance in human disease, and some have been associated with Bt resistance in lepidopterans [74]. ATUMI has fewer ABC-Cs than TCAST, but more than DMELA. At first, this might suggest a beetle-specific expansion as well as a TCAST-specific expansion. Indeed, there is a suite of expansions that may be beetle-specific (Figure 6), although comparisons to more species would be required to confirm this. However, each species also appears to have its own expansions; TCAST and ATUMI expansions are often tandem, as can be seen by the number of genes found on the same linkage groups/scaffolds (Figure 6). Indeed, there are surprisingly few clear one-to-one orthologous relationships, suggesting rapid evolution of ABC-C genes to fill species-specific

needs. To understand ATUMI responses to pesticides, these ATUMI-specific expansions may be worth further study.

Gluthatione-S-Transferase

Gluthatione-S-Transferases (GSTs) are conjugases that bind glutathione to a wide variety of substrates such as plant allelochemicals, insecticides, reactive oxygen species, and metabolic products that can provide detoxification, antioxidant, excretion, and transport functions [76], [77], [78]. Insect GSTs are widely studied due to their role in insecticide resistance [79]. Genomic analyses show that insects possess between 10 to 41 genes that encode GSTs distributed across 8 classes (i.e. Delta, Epsilon, Omega, Sigma, Theta, Zeta, Microsomal, and Unclassified) [80].

In the ATUMI genome, 49 GSTs were identified, 9 of which displayed isoforms (Figure 7; Table 5). The number of genes in the ATUMI genome is very similar to what has been identified in TCAST, especially in the Delta, Epsilon, Sigma, and Theta classes. Relative to other insects, ATUMI and TCAST have expansions in the Epsilon, Sigma, Zeta, and Microsomal GST classes, which supports the hypothesis that these may be Coleoptera-specific class expansions [80]. The small number of genes in the Delta class for both ATUMI and TCAST suggests a class contraction or lack of expansion within the beetles.

Classically, increases in the expression and activity Delta and Epsilon classes confer resistance to diverse classes of insecticides such organophosphates, organochlorines (DDT), and pyrethroids [77], [79]. These two GST classes tend to be the most numerous and dynamic in terms of expansions and contractions [80]. Therefore it would appear that ATUMI possesses a

wide diversity of GSTs, especially in the Epsilon class, to detoxify insecticides utilized for their control.

Cytochrome P450

The Cytochrome P450 monooxygenases (CYP450s) are classified as phase I metabolic enzymes which are involved in the biosynthesis, bioactivation, and regulation of endogenous compounds such as hormones, fatty acids, and sterols as well as detoxification of xenobiotic compounds such as plant alleleochemicals and insecticides. Overexpression of CYP450s often underlies high levels of detoxification-mediated insecticide resistance in many insects [81] [82] [83]. In the 69 insect genomes that have been published, more than 7,500 P450 genes have been identified in 208 families across 4 clans (CYP2, CYP3, CYP4 and Mitochondrial) [84].

In ATUMI, we found 116 genes across the 4 CYP clans (Figure 8, Table 6). The CYP2 and mitochondrial clans contained 8 and 10 genes, respectively, and orthologs were identified in other species. The conservation in sequence and number is expected as many of the genes in these clans are involved in ecdysteroid biosynthesis [85]. In contrast to the conserved CYP2 and mitochondrial clans, there are clear expansions in CYP3 and CYP4 compared to other species. These expansions are typified by large expansions of a single family that lacks orthologs in other species [86]. Within the CYP3 clan, the 55 genes are clustered in smaller blooms with the largest consisting of 13 genes. The 43 genes belonging to the CYP4 clan of ATUMI is among the largest seen in insects [7] with a noticeably large bloom of 20 genes. The species-specific expansions in the CYP3 and CYP4 clans may reflect differences in the diverse diets and chemical

communication pathways of these insects. Additionally, CYPs in the CYP3 and CYP4 clans have been implicated in insecticide resistance [87] [88] [89]. A lack of diversity in these clans may underlie the high sensitivity to insecticides in the honey bee [90]. In contrast, the rapid onset of insecticide resistance ATUMI may be facilitated by large number of CYPs in the CYP3 and CYP4 clans.

Carboxyl/Choline Esterases

Carboxyl/Choline Esterases (COEs) are capable of metabolizing a wide variety of substrates and their activity is involved in a number of physiological processes such as bioactivation of juvenile hormone and regulating acetylcholine interactions at the synapse [91] [92]. Increases in the amount of esterase expression and mutations in the catalytic site of esterases confer insecticide resistance [93] [94]. Insects possess a wide variety of COEs that are broadly classified as intracellular or dietary (Clades A-C), secreted pheromone/hormone processing (Clades D-G), and neurodevelopmental (Clades H-M) [91].

The ATUMI genome contained 60 genes encoding putative COEs, with only one displaying multiple isoforms (Figure 9). The number of genes in the secreted and neurodevelopmental groups was mostly consistent with other insects (Table 7). The expansion of Clade E (Secreted β-esterase) is consistent with a similar expansion in TCAST. This expansion is not entirely characteristic of Coleoptera as DPOND and AGLAB only have 4 and 1 member of Clade E, respectively [7]. The 10 genes for neuroligins is nearly twice the number seen in other insects [11] [90] [95]. Nevertheless, the general conservation in sequence and number suggests critical roles for these COEs across insects. In contrast to COEs in the secreted and

neurodevelopmental groups, a vast majority of ATUMI COEs in the intracellular or dietary class lacked clear orthologs in TCAST*,* AMELL*,* or DMELA. This expansion of intracellular or dietary esterases is consistent with expansions observed in other insect genomes. These species-specific expansions of intracellular or dietary esterases may be due to dietary differences among these insects. Dietary esterases may also contribute to insecticide resistance [91]. Therefore, this expansive array of dietary esterases may allow ATUMI to detoxify insecticides that may be used for control.

Implications

ATUMI is an expanding invasive pest of honey bees, disrupting managed bee colonies and arguably having a strong impact on feral on naturally occurring colonies. We anticipate the resources described here will lead to novel methods to track and control this pest. The ATUMI genome also reveals numerous evolutionary distinctions relative to other sequenced arthropods. These distinctions help clarify the sensory cues used by ATUMI and the dietary habits of this beetle, and of beetles (order Coleoptera) more broadly.

Methods

DNA extraction

ATUMI adults were collected from a population maintained by the USDA-ARS Honey Bee Breeding, Genetics and Physiology Laboratory (Baton Rouge, LA) in November 2013. ATUMI larvae were collected March 8, 2014, from a continuous culture of small hive beetles

maintained at the USDA-ARS Bee Research Laboratory. For adult beetles, extractions were carried out on individual whole male beetles using the Qiagen DNAEasy kit (n = 4 beetles extracted). Larval DNA was extracted from a total of 150 second-instar larvae in 30 groups of five larvae each. Larvae were crushed using a plastic pestle in 1ml of freshly prepared CTAB buffer consisting of 100 mM TrisHCl (pH 8.0), 20 mM EDTA (pH. 8.0), 1.4 M NaCl, 2% CTAB and 0.2% b-mercaptoethanol. The suspension was incubated at 65°C for 60 minutes, with gentle mixing at 0, 20, and 40 minutes. Samples were centrifuged for 2 min at 14k rpm (2081 g) in an Eppendorf microcentrifuge tube rotor. 500 µl of the supernatant was moved into a new tube containing using a wide-bore pipette into a sterile tube containing 500 µl chloroform:isoamylalcohol (24:1). After gentle mixing by hand, tubes were centrifuged at 14k rpm for 15 min. Approximately 400 µl of the aqueous layer was transferred into new tubes containing 250 µl cold isopropanol, followed by gentle mixing and incubation at 4°C for 30 minutes. Samples were centrifuged at 14k rpm for 30 min a 4°C, and then the supernatant was poured off. Pellets were washed with 1 ml cold 75% EtOH and centrifuged again for 2 min. (14k rpm). After the supernatant was poured off, the resulting pellets were washed in 1 ml cold 100% EtOH, centrifuged for 2 min, after which the EtOH was poured off, the pellets were spun for an additional 30 seconds, and the last of the wash was removed by pipette. Pellets were air-dried for 30 minutes and the resulting DNA pellet was resuspended in 50 µl ddH20. Samples were incubated for 30 min with 2.5 µl of an RNAse cocktail at 37°C, followed by gentle addition of 5 µl of 7M NaOAc and 100 µl EtOH. After 30 minutes of incubation on wet ice, the DNA samples were spun at 12k rpm for 30 min, washed once with 70% EtOH, dried and suspended in

20 µl ddH20. Extracts were pooled and assayed by gel electrophoresis to ensure DNA integrity and by Nanodrop (Thermofisher, Inc.) for quantification (180 ng/µl in 25 µl, 45 µg total DNA).

Phylogenetic and genetic diversity of beetles

The official protein sets of ATUMI, the red flour beetle (*Tribolium castaneum*) [10], mountain pine beetle (*Dendroctonus ponderosae*) [96], Asian longhorned beetle (*Anoplophora glabripennis*) [11], dung beetle (*Onthophagus taurus*; https://www.ncbi.nlm.nih.gov/assembly/GCA_000648695.1/), emerald ash borer (*Agrilus planipennis*; https://www.ncbi.nlm.nih.gov/assembly/GCF_000699045.1/), coffee borer beetle (*Hypothenemus hampei*) [97], burying beetle (*Nicrophorus vespilloides*) [98], scarab beetle (*Oryctes borbonicus*) [14], honey bee (*Apis mellifera*) [99], and fruit fly (*Drosophila melanogaster*) [100] were used to query the BUSCO Endopterygota ortholog set. Single copy orthologs shared by all 11 insect species were further used for phylogenetic analysis. Protein sequences of these orthologous groups (OGs) were aligned using MUSCLE [101]. Alignments were quality trimmed with trimAI [102] and the orthologous groups were concatenated for use in phylogenetic analysis. A maximum likelihood (ML) tree search was implemented using the program RAxML version 8.2.9 [103] with 1000 bootstrap replicates. The final tree was viewed and edited with TreeGraph2 [104]. Microsatellite markers were identified in the ATUMI genome assembly using the Microsatellite Search and Building Database (MSDB) package [105]. The raw Illumina gDNA reads, used to assemble the ATUMI genome, were re-aligned to the assembly using BWA [106]. The aligned reads were used to identify single nucleotide

polymorphism (SNP) positions using GATK (version 3.6; [107]), and the further annotated with SNPEFF [108].

Gustatory Receptors

The repertoire of gustatory receptors has been preliminarily characterized for TCAST [109] (62 Grs) and *A. glabripennis* [11]. Additionally, online databases have listed gustatory receptors for *T. castaneum*, including UniProtKB (www.uniprot.org [110]), and BeetleBase (www.Beetlebase.org [111]). Amino acid sequences for putative and identified *Gr* genes were compiled from these resources and truncated to remove redundancies. The compiled TCAST gene set contained 71 *Gr* genes. To identify and enumerate gustatory receptors for AGLAB and ATUMI, amino acid sequences of TCAST gustatory receptor genes were submitted to the ATUMI RefSeq gene set and genome assembly using BLASTP and TBLASTN, respectively. Putative *Gr* genes for both species were selected from hits based on an E-score ≤ to E^{-100}. Using the data set of *Gr* genes compiled for *T. castaneum*, 38 and 11 putative Gr proteins were identified for AGLAB and ATUMI, respectively. Sequences were aligned using MUSCLE [101]. The PhyML program (v3.1/3.0 aLRT) was used to build a phylogenetic tree using maximum likelihood method [41, 112]. The tree was further edited and visualized with the TreeDyn (v198.3) program [113]. All analyses from the sequence alignment to tree reconstruction were performed on the phylogeny.fr platform [114]. Sequences obtained in Newick format from this platform were used as input in the ITOL program to construct and visualize using an unrooted, circular phylogenetic tree [115].

ABC Transporters

Potential ATUMI ABC genes homologous to TCAST ABCs were identified using protein BLAST to search with each TCAST ABC sequence using WebApollo at https://i5k.nal.usda.gov/aethina-tumida. Protein sequences from ATUMI, TCAST, and DMELA were then compiled, and trimmed to exclude all but 51 residues around the Walker B motif of the nucleotide binding domain. This 51-amino-acid sequence was then used to build the phylogenetic tree (See Table 3 for the sequences used from ATUMI). The maximum likelihood phylogenetic tree was constructed using the program MEGA, version 7 [116], using default parameters in all categories except: LG model of amino-acid substitution with Gamma distributed substitution rates (based on Best Model determination within the MEGA program), and Partial Deletion treatment of gaps/missing data [117].

Insecticide targets and detoxification genes

The predicted proteins from the official gene set of ATUMI (taxid 116153) were queried with TCAST orthologs for gene families and pathway members related to insecticide resistance via BLASTP. Putative orthologs in ATUM were designated by >95% query coverage and E-value $<1E^{-100}$.

Additional files

File S1 MS-Word. Detailed material and methods.

File S2 MS-Excel. Orthology assignments for glycoside hydrolases (GHs) coded by ATUMI.

File S3 MS-Excel. Protein identifiers for orthogroup assignments.

Data and Material

Data supporting the results of this article are deposited at NCBI-Bioproject PRJNA256171.

Abbreviations

ATUMI: small hive beetle; BUSCO: Benchmarking Universal Single Copy Orthologs

Competing Interest

The authors declare no competing interest.

Funding

No external funding

Author contribution

J.D.E. and Q.H. designed the study. E.S. analyzed digestive proteins. D.M., B.D., N. G., S. C., N. E., M. L., S. R. and F. R. analyzed detoxification genes. J.D.E., D.M., B.D., N. G., S. C., N. E., M. L., S. R., P. N., F. R. and Q.H. wrote the manuscript.

Acknowledgements

We appreciate the bioinformatics support from the Competency Centre in Bioinformatics and Computational Biology, Bern, Switzerland.

Tables

Table 1. Assembly statistics of the small hive beetle genome assembly

Illumina (genome coverage)	535
PacBio (genome coverage)	50
Assembly size (Mbp)	234.3
Number of contigs	3063
Largest contig (Kbp)	2683.7
Smallest contig (Kbp)	1.26
N50 (Kbp)	298.8
Number of contig > 10 Kbp	2236
Number of contig > N50	192
Number of protein coding genes	14076
Number of mRNAs	17463
Density of SNPs (bps per SNP position)	177
Density of microsatellites (loci per Kbp)	8.23

Table 2. The number of gustatory receptor (Gr) genes from major groups for three coleopteran species; the small hive beetle, ATUMI, AGLAB, and TCAST and their putative coding for detecting either bitter or sweet tastants.

**Species**	**Gustatory receptor group**						**Tastant type**	
	2a	5a	28a/b	43a	64a-f	Total	Bitter	Sweet
**AGLAB**	11	1	7	1	6	26	19	7
**ATUMI**	3	0	2	2	4	11	5	6
**TCAST**	12	3	30	12	14	71	42	29

Table 3. Evaluation of resistance mutations in acetylcholine esterase and their status in ATUMI. *Torpedo* Ace position number, the resistant mutations are described in [68].

Torpedo Ace Position	ATUMI_Ace1 Position	Resistant Mutations	ATUMI_Ace1 State
**119**	189	G247S, G119D	G
**128**	198	D237E	D
**201**	270	A302S	A
**227**	296	G265A, G262A	G
**290**	358	F290V	F
**331**	399	S431F, F445W, F439C	F

Torpedo Ace Position	**ATUMI_Ace2 Position**	**Resistant Mutations**	**ATUMI_Ace2 State**
**78**	114	F139L, F115S	F
**82**	118	E81K	E
**129**	177	I161V/T	I
**151**	198	V180L	I
**227**	280	G265A, G262A/V	G
**238**	290	S291G	T
**290**	358	F330Y, F237Y	F
**328**	383	G365A, G368A	G
**396**	452	G488S	G

Table 4: Numbers of ABC genes in each species, by subfamily

**Species**	**Subfamily**								**Total**
	**A**	**B**	**C**	**D**	**E**	**F**	**G**	**H**	
ATUMI	4*	6	24	2	1	3	13*	3	56
TCAST	10	6	35	2	1	3	14	3	74
DMELA	10	8	14	2	1	3	15	3	56

Table 5. Comparison of the number of GSTs between ATUMI, AMELL, DMELA, and TCAST [80], [90].

GST Class	ATUMI	AMELL	DMELA	TCAST
**Delta**	3	1	11	3
**Epsilon**	19	0	14	19
**Omega**	1	1	5	3
**Sigma**	7	4	1	7
**Theta**	1	1	4	1
**Zeta**	5	1	2	1
**Microsomal**	6	2	1	5
**Unclassified**	7	0	0	2
**Total**	49	10	38	41

Table 6. Comparison of CYP450 genes in ATUMI, AMELL, DMELA, and TCAST.

**P450 Clan**	**ATUMI**	**AMELL**	**DMELA**	**TCAST**
**CYP2**	8	8	6	8
**CYP3**	55	28	36	82
**CYP4**	43	4	32	49
**Mitochondrial**	10	6	11	10
**Total**	116	46	85	149

665

666 Table 7. Comparison of COE from *Aethina tumida* to *Drosophila melanogaster*, *Tribolium* 667 *castaneum*, and *Apis mellifera*. Nomenclature and gene counts follow McKenna 2016 [11]and 668 Claudianos 2006. [90].

COE Subfamily	*ATUMI*	*AMELL*	*DMELA*	*TCAST*
**Clades A-C (Dietary)**	27	8	13	55
**Clade D (Integument Esterases)**	2	1	3	5
**Clade E (Secreted β-esterase)**	8	3	3	10
**Clade F (JH Esterases)**	3	1	2	1
**Clade H (Glutactins)**	2	0	4	2
**Clade I (Unknown Function)**	1	2	2	2
**Clade J (Acetylcholinesterases)**	2	2	1	2
**Clade K (Gliotactin)**	1	1	1	2
**Clade L (Neuroligins)**	10	5	4	5
**Clade M (Neurotactins)**	4	1	2	1
**Total**	60	24	35	85

669

670

Figure Legends

Figure 1. *Aethina tumida* (A) adult and (B) larva. Photos courtesy of Alex Wild Photography, used with permission.

Figure 2. Phylogenetic tree and estimated completeness of the genomes of 11 insect species. A) The phylogenetic tree was constructed on protein sequences of 181 single copy orthologs shared among all 11 insect species. All nodes have 100% bootstrap support. AMELL and DMELA were used as outgroups. Branch lengths are shown for each node. B) Completeness of official protein sets of each insect species were assessed by aligning to the Endopterygota sets of benchmarking universal single-copy orthologs (BUSCOs). For ATUMI, 97.5% of complete BUSCOs were found. C) The pervasiveness of gene loss during endopterygote evolution. From the domain counts of lost BUSCOs, methyltransferase (MT), glycosyltransferase (GT) and leucine rich repeats (LRR) are among the top 5% of total domains and are commonly lost from multiple species. WD and ZF red boxes indicate that the gene is lost, while white boxes indicate that the gene is maintained in each species.

Figure 3. Gene duplication events plotted against the average gene duplication event per gene. The protein sets of the small hive beetle, red flour beetle, mountain pine beetle, Asian longhorned beetle, dung beetle, emerald ash borer, coffee borer beetle, burying beetle, scarab beetle, honey bee and fruit fly were searched against the Endopterygota BUSCO set using BLAST. Redundant proteins (including recent paralogs and those with known alternative

splicing) were used to quantify the average number of duplication events per gene in each species.

Figure 4. Glycoside hydrolase (GH) family copy numbers identified from beetle genomes. Genes coding for glycoside hydrolases were identified using Pfam domain assignments [118] and genome assemblies and coding gene predictions were obtained from NCBI (GenBank Accession Numbers: GCA_000390285.1 AGLAB, GCA_000355655.1 DPOND, GCA_001412225.1 *N. vespilloides*, GCA_001443705.1 *O. borbonicus*, GCA_000002335.3 TCAST) with the exception of *H.s hampei*, which was downloaded from https://genome.med.nyu.edu/coffee-beetle/cbb.html. Families are color coded from green to read based on their relative abundance (total count/total number of GH genes) with red representing GH families that are highly abundant (≥25% of the total GH genes) and green representing GH families of lesser abundance (≤0.01%). Notably, the GH profiles of ATUMI and TCAST (neither of which feed on living plant material) differ strongly from the GH profiles of the phytophagous beetles, even though they all belong to the same infraorder, suggesting that diet, in part, might be driving the differences in GH family members and copy numbers. AGLAB=Asian longhorned beetle (*A. glabripennis*); HHAMP=Coffee berry borer (*H. hampei*); DPOND=Mountain pine beetle (*D. ponderosae*); NVESP=burying beetle (*N. vespilloides*); OBORB=scarab beetle (*O. borbonicus*); ATUMI=small hive beetle, and TCAST=red flour beetle (TCAST).

Figure 5. Maximum likelihood cladogram for gustatory receptor genes from three coleopteran species. The small hive beetle, *Aethina tumida* (*ATUMI*; green labels/lines), the Asian long horned beetle, *Anoplophora glabripennis* (*Agla*; red labels/lines), and the red flour beetle,

Tribolium castaneum (*TCAST*; blue labels/lines). Individual genes are labeled with species identifier and GenBank accession number. Scale bar for branch lengths represents 0.1 amino acid substitutions per site. Ring around cladogram indicates gene families coded for perceiving bitter (yellow) and sweet (pink) tastants.

Figure 6. Maximum likelihood phylogenetic tree of ABC proteins from ATUMI **(At), TCAST (Tc), and DMELA (Dm).** ATUMI genes are marked in blue, TCAST in green, and DMELA in purple. ABC subfamilies are indicated with colored lines to the right of the tree. Names for DMELA proteins were taken from Flybase (http://flybase.org/reports/FBgg0000552), and include the Flybase number for reference. TCAST names are taken from the two papers in which the genes were identified [75, 119], with the NCBI Refseq accession number provided for reference. ATUMI names were generated for this paper by combining the subfamily of the identified sequence with the scaffold on which the encoding gene may be found; if multiple ABC genes of a particular subfamily were found on the same scaffold, the sequences were given an additional letter designation based on their relative location, reading left to right on the scaffold as shown in WebApollo. For reference, the scaffold number and base coordinates for the gene have also been included.

Figure 7. Maximum Likelihood phylogenetic tree of glutathione-S-transferase (GST) proteins. The bootstrap consensus tree inferred from 1000 replicates is taken to represent the evolutionary history of the taxa *A. tumida* (ATUMI) in green; *A. mellifera* (AMELL) in black; *D. melanogaster* (DMELA) in blue; and *T. castaneum* (Tribol) in red identified manually using the

Uniprot and Pfam databases. Branches corresponding to partitions reproduced in less than 50% bootstrap replicates are collapsed. Initial tree(s) for the heuristic search were obtained automatically by applying Neighbor-Join and BioNJ algorithms to a matrix of pairwise distances estimated using a JTT model, and then selecting the topology with superior log likelihood value. All positions with less than 95% site coverage were eliminated. The tree was annotated and visualized with the iToL web tool (itol.embl.de/).

Figure 8. Maximum Likelihood phylogenetic tree of the cytochrome P450 detoxification system. The bootstrap consensus tree inferred from 1000 replicates is taken to represent the evolutionary history of the taxa *A. tumida* (ATUMI) in green; *A. mellifera* (AMELL) in black; *D. melanogaster* (DMELA) in blue; and *T. castaneum* (Tribol) in red identified manually using the Uniprot and Pfam databases. Branches corresponding to partitions reproduced in less than 50% bootstrap replicates are collapsed. Initial tree(s) for the heuristic search were obtained automatically by applying Neighbor-Join and BioNJ algorithms to a matrix of pairwise distances estimated using a JTT model, and then selecting the topology with superior log likelihood value. All positions with less than 95% site coverage were eliminated. P450s are clustered to CYP2, CYP3, CYP4 and mitochondrial clans. The tree was annotated and visualized with the iToL web tool (itol.embl.de/) [120].

Figure 9. Maximum likelihood phylogenetic tree of carboxylesterase (COE) genes. The maximum likelihood bootstrap consensus tree (1000 replicates) showing the relationships among COE genes from the genomes of *A. tumida* (ATUMI) in green; *A. mellifera* (AMELL) in

black; *D. melanogaster* (DMELA) in blue; and *T. castaneum* (Tribol) in red, identified manually using the Uniprot and Pfam databases. Branches corresponding to partitions recovered in less than 50% of bootstrap replicates are collapsed. Starting tree(s) for the heuristic search were obtained automatically using neighbor-joining and BioNJ algorithms applied to a matrix of pairwise distances estimated using a JTT model, and then selecting the topology with the superior log likelihood value. All positions with less than 95% site coverage were eliminated. The phylogenetically distinct clusters were named according to established nomenclature for COE genes [11]. The tree was annotated and visualized with the iToL web tool [120].

References

1. Lundie AE. The small hive beetle *Aethina tumida*, Science Bulletin 220. In: Forestry DoAa, (ed.). Pretoria, South Africa1940.
2. Neumann P, Pettis JS and Schäfer MO. Quo vadis Aethina tumida? Biology and control of small hive beetles. Apidologie. 2016;47 3:427-66. doi:10.1007/s13592-016-0426-x.
3. Al-Toufailia H, Alves DA, Bená DDC, Bento JMS, Iwanicki NSA, Cline AR, et al. First record of small hive beetle, Aethina tumida Murray, in South America. Journal of Apicultural Research. 2017;56 1:76-80. doi:10.1080/00218839.2017.1284476.
4. Lee S, Hong KJ, Cho YS, Choi YS, Yoo MS and Lee S. Review of the subgenus Aethina Erichson s. str. (Coleoptera: Nitidulidae: Nitidulinae) in Korea, reporting recent invasion

of small hive beetle, Aethina tumida. Journal of Asia-Pacific Entomology. 2017;20 2:553-8. doi:10.1016/j.aspen.2017.03.006.

5. Buchholz S, Schäfer MO, Spiewok S, Pettis JS, Duncan M, Ritter W, et al. Alternative food sources of Aethina tumida (Coleoptera: Nitidulidae). Journal of Apicultural Research. 2008;47 3:202-9. doi:10.3827/IBRA.1.47.3.08.

6. Neumann P, Pirk CWW, Hepburn HR, Solbrig AJ, Ratnieks FLW, Elzen PJ, et al. Social encapsulation of beetle parasites by Cape honeybee colonies (Apis mellifera capensis Esch.). Naturwissenschaften. 2001;88 5:214-6. doi:10.1007/s001140100224.

7. Haddad S and Mckenna DD. Phylogeny and evolution of the superfamily Chrysomeloidea (Coleoptera: Cucujiformia). Systematic Entomology. 2016;41 4:697-716.

8. McKenna DD, Wild AL, Kanda K, Bellamy CL, Beutel RG, Caterino MS, et al. The beetle Tree of Life reveals Coleoptera survived end Permian mass extinction to diversify during the Cretaceous terrestrial revolution. Systematic Entomology. 2015:1-46. doi:10.1111/syen.12132.

9. McKenna DD. Beetle genomes in the 21st century: prospects, progress and priorities. . Current Opinion in Insect Science 2018;25 76-82.

10. Richards S, Gibbs RA, Weinstock GM, Brown SJ, Denell R, Beeman RW, et al. The genome of the model beetle and pest Tribolium castaneum. Nature. 2008;452 7190:949-55. doi:10.1038/nature06784.

11. McKenna DD, Scully ED, Pauchet Y, Hoover K, Kirsch R, Geib SM, et al. Genome of the Asian longhorned beetle (Anoplophora glabripennis), a globally significant invasive species, reveals key functional and evolutionary innovations at the beetle-plant interface. Genome Biology. 2016;17 227 doi:10.1186/s13059-016-1088-8.

12. Keeling IC, Yuen MSM, Liao YN, Docking TR, Chan KSSK, Taylor AG, et al. Draft genome of the mountain pine beetle, Dendroctonus ponderosae Hopkins, a major forest pest. Genome Biology. 2013;14 3 doi:10.1186/gb-2013-14-3-r27.

13. Vega FE, Brown SM, Chen H, Shen E, Nair MB, Ceja-Navarro JA, et al. Draft genome of the most devastating insect pest of coffee worldwide: The coffee berry borer, Hypothenemus hampei. Scientific Reports. 2015;5 doi:10.1038/srep12525.

14. Meyer JM, Markov GV, Baskaran P, Herrmann M, Sommer RJ and Rödelsperger C. Draft Genome of the Scarab Beetle Oryctes borbonicus on La Réunion Island. Genome Biology and Evolution. 2016;8 7:2093-15.

15. Cunningham CB, Ji L, Wiberg RAW, Shelton J, McKinney EC, Parker DJ, et al. The genome and methylome of a beetle with complex social behavior, *Nicrophorus vespilloides* (Coleoptera: Silphidae). Genome Biology and Evolution. 2015;7 12:3383-96. doi:10.1093/gbe/evv194.

16. Schoville SD, Chen YH, Andersson MN, Benoit JB, Bhandari A, Bowsher JH, et al. A model species for agricultural pest genomics: the genome of the Colorado potato beetle, Leptinotarsa decemlineata (Coleoptera: Chrysomelidae). bioRxiv. 2017; doi:http://dx.doi.org/10.1101/192641.

17. Eyer M, Chen YP, Schäfer MO, Pettis J and Neumann P. Small hive beetle, Aethina tumida, as a potential biological vector of honeybee viruses. Apidologie. 2009;40 4:419-28. doi:10.1051/apido:2008051.

18. Schäfer MO, Ritter W, Pettis J and Neumann P. Small hive beetles, Aethina tumida, are vectors of Paenibacillus larvae. Apidologie. 2010;41 1:14-20. doi:10.1051/apido/2009037.

19. Torto B, Arbogast RT, Van Engelsdorp D, Willms S, Purcell D, Boucias D, et al. Trapping of Aethina tumida murray (Coleoptera: Nitidulidae) from Apis mellifera L. (Hymenoptera:

Apidae) colonies with an in-hive baited trap. Environmental Entomology. 2007;36 5:1018-24. doi:10.1603/0046-225X(2007)36[1018:TOATMC]2.0.CO;2.

20. Torto B, Boucias DG, Arbogast RT, Tumlinson JH and Teal PEA. Multitrophic interaction facilitates parasite-host relationship between an invasive beetle and the honey bee. Proceedings of the National Academy of Sciences of the United States of America. 2007;104 20:8374-8.

21. Torto B, Arbogast RT, Van Engelsdorp D, Willms S, Purcell D, Boucias D, et al. Trapping of *Aethina tumida* Murray (Coleoptera: Nitidulidae) from *Apis mellifera* L.(Hymenoptera: Apidae) colonies with an in-hive baited trap. Environmental entomology. 2014;36 5:1018-24.

22. Benda ND, Boucias D, Torto B and Teal P. Detection and characterization of Kodamaea ohmeri associated with small hive beetle Aethina tumida infesting honey bee hives. Journal of Apicultural Research. 2008;47 3:194-201.

23. Conklin TM. Investigations of small hive beetle-yeast associations. 2012.

24. Graham JR. *The attraction of bumble bee (Hymenoptera: Apidae, Bombus impatiens Cresson) colonies to small hive beetles (Coleoptera: Nitidulidae, Aethina tumida Murray)*. University of Florida, 2009.

25. Arbogast RT, Torto B, Van Engelsdorp D and Teal PEA. An effective trap and bait combination for monitoring the small hive beetle, Aethina tumida (Coleoptera: Nitidulidae). Florida Entomologist. 2007;90 2:404-6. doi:10.1653/0015-4040(2007)90[404:AETABC]2.0.CO;2.

26. Arbogast RT, Torto B and Teal PEA. Monitoring the small hive beetle aethina tumida (coleoptera: Nitidulidae) with baited flight traps: Effect of distance from bee hives and shade on the numbers of beetles captured. Florida Entomologist. 2009;92 1:165-6. doi:10.1653/024.092.0129.

27. Ye C, Ma ZS, Cannon CH, Pop M and Yu DW. Exploiting sparseness in de novo genome assembly. BMC Bioinformatic. 2012; doi:10.1186/1471-2105-13-S6-S1.

28. Hackl TH, R.; Schultz, J.; Foerster, F. . proovread: large-scale high accuracy PacBio correction through iterative short read consensus. . 2014.

29. Ye C MZ. Sparc: a sparsity-based consensus algorithm for long erroneous sequencing reads. 2016; doi:10.7717.

30. Tarver MR, Huang Q, de Guzman L, Rinderer T, Holloway B, Reese J, et al. Transcriptomic and functional resources for the small hive beetle Aethina tumida, a worldwide parasite of honey bees. Genomics Data. 2016;9:97-9. doi:10.1016/j.gdata.2016.06.003.

31. Kim. TopHat2: accurate alignment of transcriptomes in the presence of insertions, deletions and gene fusions. Genome biology. 2013.

32. Simão FA, Waterhouse RM, Ioannidis P, Kriventseva EV and Zdobnov EM. BUSCO: assessing genome assembly and annotation completeness with single-copy orthologs. . Bioinformatics 2015;31 19:3210-2. doi:10.1093/bioinformatics/btv351.

33. Emms DM and Kelly S. OrthoFinder: solving fundamental biases in whole genome comparisons dramatically improves orthogroup inference accuracy. Genome Biology. 2015;16:157.

34. Pauchet Y, Wilkinson P and Chauhan R. Diversity of the beetle genes encoding novel plant cell wall degrading enzymes. PloS One. 2010;5:e15635.

35. Merzendorfer H and Zimoch L. Chitin metabolism in insects: structure, function and regulation of chitin synthases and chitinases. Journal of Experimental Biology. 2003;206:4393-412.

36. Kramer KJ and Muthukrishnan S. Insect chitinases: molecular biology and potential use as biopesticides. Insect Biochemistry and Molecular Biology. 1997;27:887-900.

37. Fujita Kea. A chitinase structurally related to the glycoside hydrolase family 48 is indispensable for hormonally induced diapause termination in a beetle. Biochemical and Biophysical Research Communications. 2006;345:502-7.

38. Peterson TNea. SignalP 4.0: discriminating signal peptides from transmembrane regions. Nature Methods. 2011;8:785-6.

39. Pacini E. Types and meaning of pollen carbohydrate reserves. Sexual Plant Reproduction. 1996;362.

40. Engel P, Martinson VG and Moran NA. Functional diversity within the simple gut microbiota of the honey bee. Proceedings of the National Academy of Sciences of the United States of America. 2012;109 27:11002-7.

41. Guindon S and Gascuel O. A simple, fast, and accurate algorithm to estimate large phylogenies by maximum likelihood. Syst Biol. 2003;52 5:696-704.

42. Trzaskowski B, Latek D, Yuan S, Ghoshdastider U, Debinski A and Filipek S. Action of Molecular Switches in GPCRs - Theoretical and Experimental Studies. Current Medicinal Chemistry. 2012;19 8:1090-109. doi:10.2174/092986712799320556.

43. Clyne PJ, Warr CG and Carlson JR. Candidate Taste Receptors in Drosophila. Science. 2000;287 5459:1830.

44. Ishimoto H, Takahashi K, Ueda R and Tanimura T. G-protein gamma subunit 1 is required for sugar reception in Drosophila. The EMBO Journal. 2005;24 19:3516.

45. Murakami M and Kijima H. Transduction Ion Channels Directly Gated by Sugars on the Insect Taste Cell. The Journal of General Physiology. 2000;115 4:455.

46. Thorne N, Chromey C Fau - Bray S, Bray S Fau - Amrein H and Amrein H. Taste perception and coding in Drosophila. Current Biology. 2004;14 12:1065-79.

47. Wang Z, Singhvi A Fau - Kong P, Kong P Fau - Scott K and Scott K. Taste representations in the Drosophila brain. Cell. 2004;117 7:981-91.

48. Isono K and Morita H. Molecular and cellular designs of insect taste receptor system. Frontiers in Cellular Neuroscience. 2010;4:20.

49. Willcox BK, Howlett BG and Neumann P. Absence of small hive beetles from flowering plants. Journal of Apicultural Research. 2017;56 5:643-5,. doi:0.1080/00218839.2017.1355347.

50. Robertson HM and Wanner KW. The chemoreceptor superfamily in the honey bee, Apis mellifera: Expansion of the odorant, but not gustatory, receptor family. Genome Research. 2006;16 11:1395-403. doi:10.1101/gr.5057506.

51. Amrein H. An expression system for Gustatory receptors—and why it failed. Fly. 2014;8 4:232-3. doi:10.1080/19336934.2015.1039756.

52. Freeman EG, Wisotsky Z and Dahanukar A. Detection of sweet tastants by a conserved group of insect gustatory receptors. Proceedings of the National Academy of Sciences. 2014;111 4:1598-603. doi:10.1073/pnas.1311724111.

53. Consortium THGS. Insights into social insects from the genome of the honeybee Apis mellifera. Nature 2006;443:931-49. doi:10.1038/nature05260.

54. Wanner KW and Robertson HM. The gustatory receptor family in the silkworm moth *Bombyx mori* is characterized by a large expansion of a single lineage of putative bitter receptors. Insect Molecular Biology. 2008;17 6:621-9. doi:10.1111/j.1365-2583.2008.00836.x.

55. Catterall WA. From ionic currents to molecular mechanisms: the structure and function of voltage-gated sodium channels. Neuron. 2000;26:13-25.

56. Du Y, Nomura Y, Satar G, Hu Z, Nauen R, He SY, et al. Molecular evidence for dual pyrethroid-receptor sites on a mosquito sodium channel. PNAS. 2013;110 29:11785-90. doi:10.1073/pnas.1305118110.

57. O'Reilly AO, Khambay BPS, Williamson MS, Field LA, Wallace BA and Davies TGE. Modelling insecticide-binding sites in the voltage-gated sodium channel. Biochem J. 2006;396:255-63.

58. Rinkevich FD, Du Y and Dong K. Diversity and convergence of sodium channel mutations involved in resistance to pyrethroids Pestic Biochem Physiol. 2013;106:93-100.

59. Lee SH, Ingles PJ, Knipple DC and Soderlund DM. Developmental regulation of alternative exon usage in the house fly *Vssc1* sodium channel gene. Invert Neurosci. 2002;4:125-33.

60. Shao YM, Dong K, Tang ZH and Zhang CX. Molecuar characterization of a sodium channel gene from the silkworm *Bombyx mori*. Insect Biochem Molec Biol. 2009;39:145-51.

61. Davies T, Field L, Usherwood P and Williamson M. A comparative study of voltage-gated sodium channels in the Insecta: implications of pyrethroid resistance in Anopheline and other Neopteran species. Insect Molecular Biology. 2007;16 3:361-75.

62. Ingles PJ, Adams PM, Knipple DC and Soderlund DM. Characterization of voltage-sensitive sodium channel gene coding sequences from insecticide-susceptible and knockdown-resistant house fly strains. Insect Biochem Molec Biol. 1996;26 4:319-26.

63. Thackeray JR and Ganetzky B. Developmentally regulated alternative splicing generates a complex array of *Drosophila para* sodium channel isoforms. J Neurosci. 1994;14 5:2569-78.

64. Ellis JD and Delaplane KS. The effects of three acaricides on the developmental biology of small hive beetles (Aethina tumida). Journal of Apicultural Research. 2007;46 4:256-9.

65. Kanga LH and Somorin AB. Susceptibility of the small hive beetle, Aethina tumida (Coleoptera: Nitidulidae), to insecticides and insect growth regulators. Apidologie. 2012;43 1:95-102.

956 66. Baxter J, Eischen F, Pettis J, Wilson W and Shimanuki H. Detection of fluvalinate-resistant *Varroa* mites in US honey bees. Am Bee J. 1998;138 4:291.

958 67. Fournier D and Mutero A. Modification of acetylcholinesterase as a mechanism of resistance to insecticides. Comparative Biochemistry and Physiology \108C. 1994:19-31.

960 68. Fournier D. Mutations of acetylcholinesterase which confer insecticide resistance in insect populations. Chemico-Biol Inteact. 2005;157:257-61.

962 69. Baek JH, Kim JI, Lee D-W, Chung BK, Miyata T and Lee SH. Identification and characterization of ace1-type acetylcholinesterase likely associated with organophosphate resistance in Plutella xylostella. Pesticide Biochemistry and Physiology. 2005;81 3:164-75.

966 70. Kim YH and Lee SH. Which acetylcholinesterase functions as the main catalytic enzyme in the Class Insecta? Insect Biochemistry and Molecular Biology. 2013;43:47-53.

968 71. Neumann P and Hoffmann D. Small hive beetle diagnosis and control in naturally infested honeybee colonies using bottom board traps and CheckMite + strips. Journal of Pest Science. 2008;81 1:43-8.

971 72. Sammataro D, Untalan P, Guerrero F and Finley J. The resistance of varroa mites (Acari: Varroidae) to acaricides and the presence of esterase. International Journal of Acarology. 2005;31 1:67-74.

974 73. Dmitryjuk M, Zółtowska K, Fraczek R and Lipiński Z. Esterases of Varroa destructor (Acari: Varroidae), parasitic mite of the honeybee. Experimental and Applied Acarology. 2014;62 4:499-510.

977 74. Dermauw W and Van Leeuwen T. The ABC gene family in arthropods: comparative genomics and role in insecticide transport and resistance. Insect Biochemistry and Molecular Biology. 2014;45:89-110.

75. Grubbs N, Haas S, Beeman RW and Lorenzen MD. The ABCs of eye color in Tribolium castaneum: orthologs of the Drosophila white, scarlet, and brown Genes. Genetics. 2015;199 3:749-59.

76. Simon JY. Insect glutathione S-transferases. Zoological Studies. 1996;35 1:9-19.

77. Che-Mendoza A, Penilla RP and Rodríguez DA. Insecticide resistance and glutathione S-transferases in mosquitoes: a review. African Journal of Biotechnology,. 2009;8 8.

78. Corona M and Robinson GE. Genes of the antioxidant system of the honey bee: annotation and phylogeny. nsect molecular biology. 2006;15 5:687-701.

79. Enayati AA, Ranson H and Hemingway J. Insect glutathione transferases and insecticide resistance. Insect molecular biology. 14 1:3-8.

80. Shi H, Pei L, Gu S, Zhu S, Wang Y and al. e. Glutathione S-transferase (GST) genes in the red flour beetle, Tribolium castaneum, and comparative analysis with five additional insects. Genomics. 2012;100 5:327-35.

81. Daborn PJ, Yen JL, Bogwitz MR, Le Goff G, Feil E and al. e. A single P450 Allele associated with insecticide resistance in *Drosophila*. Science. 2002;297 5590:2253-6.

82. Liu N and Scott JG. Increased transcription of CYP6D1 causes cytochrome P450-mediated insecticide resistance in house fly. Insect Biochem Molec Biol. 1998;28:531-5.

83. Hardstone MC, Komagata O, Kasai S, Tomita T and Scott JG. Use of isogenic strains indicates CYP9M10 is linked to permethrin resistance in Culex pipiens quinquefasciatus. Insect Molec Biol. 2010;19:717-26.

84. Nelson DR, 2018. Cytochrome P450 diversity in the tree of life. Biochimica et Biophysica Acta (BBA)-Proteins and Proteomics, 1866(1), pp.141-154. Cytochrome P450 diversity in the tree of life. iochimica et Biophysica Acta (BBA)-Proteins and Proteomics. 2018;1866 1:141-54.

85. Gilbert LI. Halloween genes encode P450 enzymes that

mediate steroid hormone biosynthesis in Drosophila melanogaster. Mol Cell Endocrinol. 2004;215:1-10.

86. Feyereisen R. **Arthropod CYPomes illustrate the tempo and mode in P450 evolution**. Biochimica et Biophysica Acta (BBA)-Proteins and Proteomics. 2011;1841 1:19-28.

87. Daborn P, Boundy S, Yen J and Pittendrigh B. DDT resistance in Drosophila correlates with Cyp6g1 over-expression and confers cross-resistance to the neonicotinoid imidacloprid. Molecular Genetics and Genomics. 2001;266 4:556-63.

88. Scott JG and Wen Z. Cytochromes P450 of insects: the tip of the iceberg. Pest management science. 2001;57 10:958-67.

89. Pridgeon JW, Zhang L and Liu N. Overexpression of CYP4G19 associated with a pyrethroid-resistant strain of the German cockroach, Blattella germanica (L.). Genetics. 2003;314:157-63.

90. Claudianos C, Ranson H, Johnson RM, Biswas MA and Schuler ea. A deficit of detoxification enzymes: pesticide sensitivity and environmental response in the honeybee. Insect Molecular Biology. 2006;15 5:615-36.

91. Oakeshott JG, Claudianos C, Campbell PM, Newcomb RD and Russell RJ. **Biochemical genetics and genomics of insect esterases**. In: Gilbert LI, Iatrou K and Gill SS, editors. Comprehensive Molecular Insect Science. Boston: Elsevier; 2005. p. 309-81.

92. Montella IR, Schama R and Valle D. The classification of esterases: an important gene family involved in insecticide resistance-A review. Memorias do Instituto Oswaldo Cruz 2012:437-49.

93. Bass C and Field LM. Gene amplification and insecticide resistance. Pest Management Science. 2011;67:886-90.

94. Newcomb RD, Campbell PM, Ollis DL, Cheah E, Russell RJ and Oakeshott JG. A single amino acid substitution converts a carboxylesterase to an organophosphorus hydrolase and confers insecticide resistance on a blowfly. Proceedings of the National Academy of Sciences 1997;94:7464-8.

95. Yu QY, Lu C, Li WL, Xiang ZH and Zhang Z. nnotation and expression of carboxylesterases in the silkworm, Bombyx mori. BMC genomics. 2009;10 1.

96. Keeling CIea. Draft genome of the mountain pine beetle, Dendroctonus ponderosae Hopkins, a major forest pest. 2013 14:R27.

97. Vega. Draft genome of the most devastating insect pest of coffee worldwide: the coffee berry borer, Hypothenemus hampei. Scientific Reports. 2015;5:12525.

98. Cunningham CB, Ji L, Axel R, Wiberg W, Shelton J, McKinney EC, et al. The Genome and Methylome of a Beetle with Complex Social Behavior, Nicrophorus vespilloides (Coleoptera: Silphidae) Genome Biol Evol 2015;7 12:3383-96. doi:10.1093/gbe/evv194.

99. Elsik CG. Finding the missing honey bee genes: lessons learned from a genome upgrade. BMC Genomics. 2014;15 86 doi:doi.org/10.1186/1471-2164-15-86.

100. Adams. The genome sequence of Drosophila melanogaster. . Science. 2000.

101. Edgar RC. MUSCLE: multiple sequence alignment with high accuracy and high throughput. Nucleic Acids Res. 2004;32 5:1792-7. doi:10.1093/nar/gkh340.

102. Capella-Gutierrez S, Silla-Martinez JM and Gabaldon T. trimAl: a tool for automated alignment trimming in large-scale phylogenetic analyses. Bioinformatics. 2009;25:1972-3.

103. Stamatakis A. RAxML Version 8: A tool for Phylogenetic Analysis and Post-Analysis of Large Phylogenies. Bioinformatics. 2014.

104. Stöver BC and Müller KF. TreeGraph 2: Combining and visualizing evidence from different phylogenetic analyses. BMC bioinformatics. 2010;11 1.

105. Du L, Li D, Zhang X and Yue B. MSDB: a user-friendly program for reporting distribution and building databases of microsatellites from genome sequences. . Journal of Heredity. 2013;104 1:154-7.

106. Li H and Durbin R. Fast and accurate short read alignment with Burrows-Wheeler Transform. Bioinformatics. 2009;25:1754-60.

107. McKenna A HM, Banks E, Sivachenko A, Cibulskis K, Kernytsky A, Garimella K, Altshuler D, Gabriel S, Daly M, DePristo MA. The Genome Analysis Toolkit: a MapReduce framework for analyzing next-generation DNA sequencing data Genome Research. 2010;20:1297-303.

108. Cingolani P PA, Wang le L, Coon M, Nguyen T, Wang L, Land SJ, Lu X, Ruden DM. A program for annotating and predicting the effects of single nucleotide polymorphisms, SnpEff: SNPs in the genome of Drosophila melanogaster strain w1118; iso-2; iso-3. 2012;6 2:80-92.

109. Abdel-latief M. A Family of Chemoreceptors in Tribolium castaneum (Tenebrionidae: Coleoptera). PLOS ONE. 2007;2 12:e1319. doi:10.1371/journal.pone.0001319.

110. Consortium TU. UniProt: the universal protein knowledgebase. Nucleic Acids Res. 2017;45 D1:D158-D69. doi:10.1093/nar/gkw1099.

111. Kim HS, Murphy T, Xia J, Caragea D, Park Y, Beeman RW, et al. BeetleBase in 2010: revisions to provide comprehensive genomic information for Tribolium castaneum. Nucleic Acids Res. 2010;38:D437-D42. doi:10.1093/nar/gkp807.

112. Anisimova M and Gascuel O. Approximate likelihood-ratio test for branches: A fast, accurate, and powerful alternative. Syst Biol. 2006;55 4:539-52. doi:10.1080/10635150600755453.

113. Chevenet F, Brun C, Banuls AL, Jacq B and Christen R. TreeDyn: towards dynamic graphics and annotations for analyses of trees. BMC Bioinformatics. 2006;7:439. doi:10.1186/1471-2105-7-439.

114. Dereeper A, Guignon V, Blanc G, Audic S, Buffet S, Chevenet F, et al. Phylogeny.fr: robust phylogenetic analysis for the non-specialist. Nucleic Acids Res. 2008;36 Web Server issue:W465-9. doi:10.1093/nar/gkn180.

115. Letunic I and Bork P. Interactive Tree Of Life (iTOL): an online tool for phylogenetic tree display and annotation. Bioinformatics. 2007;23 1:127-8. doi:10.1093/bioinformatics/btl529.

116. Kumar S, Stecher G and Tamura K. MEGA7: Molecular Evolutionary Genetics Analysis version 7.0 for bigger datasets. Molecular biology and evolution. 2016:msw054.

117. Hall BG. Building Phylogenetic Trees from Molecular Data with MEGA. Molecular Biology and Evolution. 2013;30 5:1229-35.

118. Bateman A. The Pfam protein families database. Nucleic Acids Res. 2002;30:276-80.

119. Broehan G, Kroeger T, Lorenzen M and Merzendorfer H. Functional analysis of the ATP-binding cassette (ABC) transporter gene family of Tribolium castaneum. BMC Genomics. 2013;14 6.

120. Letunic I and Bork P. Interactive tree of life (iTOL) v3: an online tool for the display and annotation of phylogenetic and other trees. Nucleic Acids Res. 2016;44 W1:W242-W5.

Figures1-9

Click here to download Figure EvansetalFigures.pdf

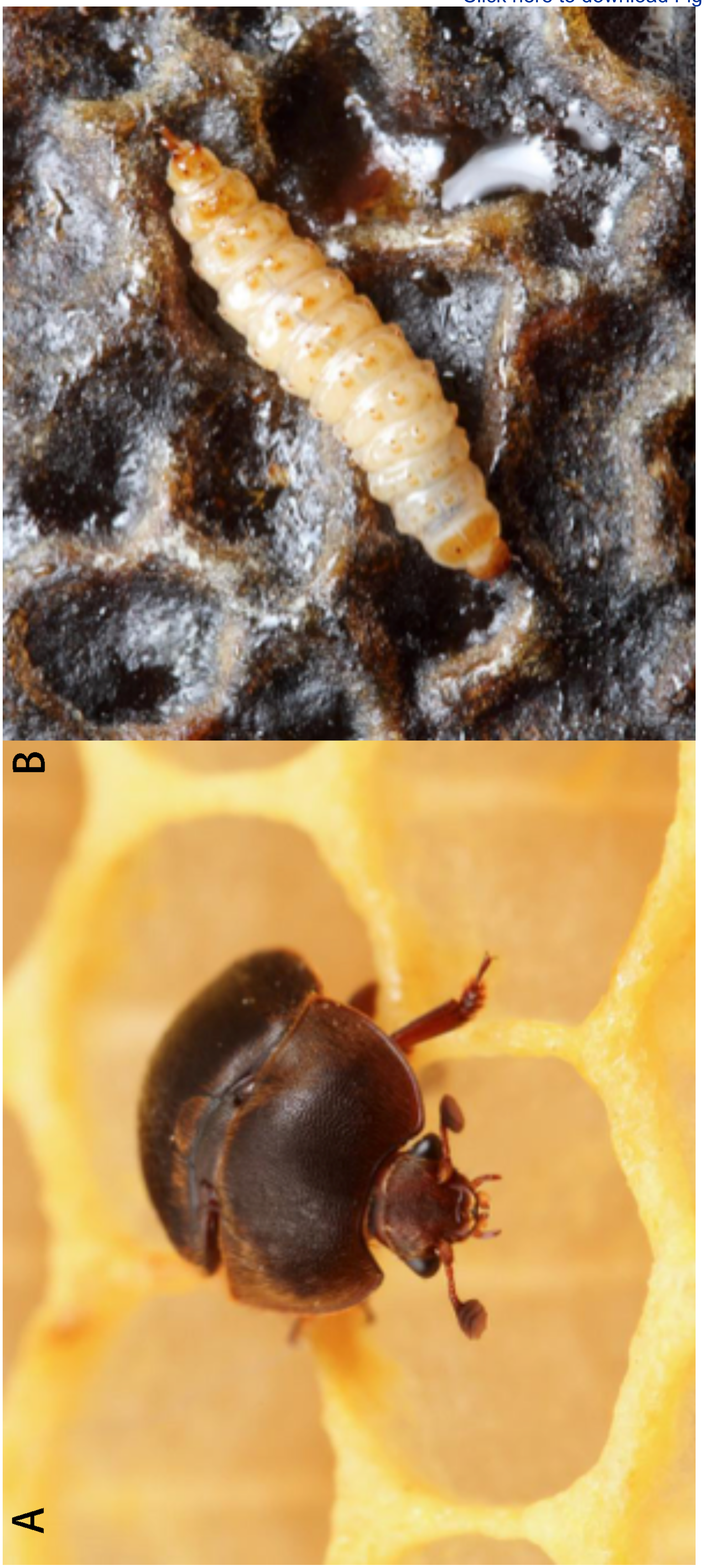

Figure 1

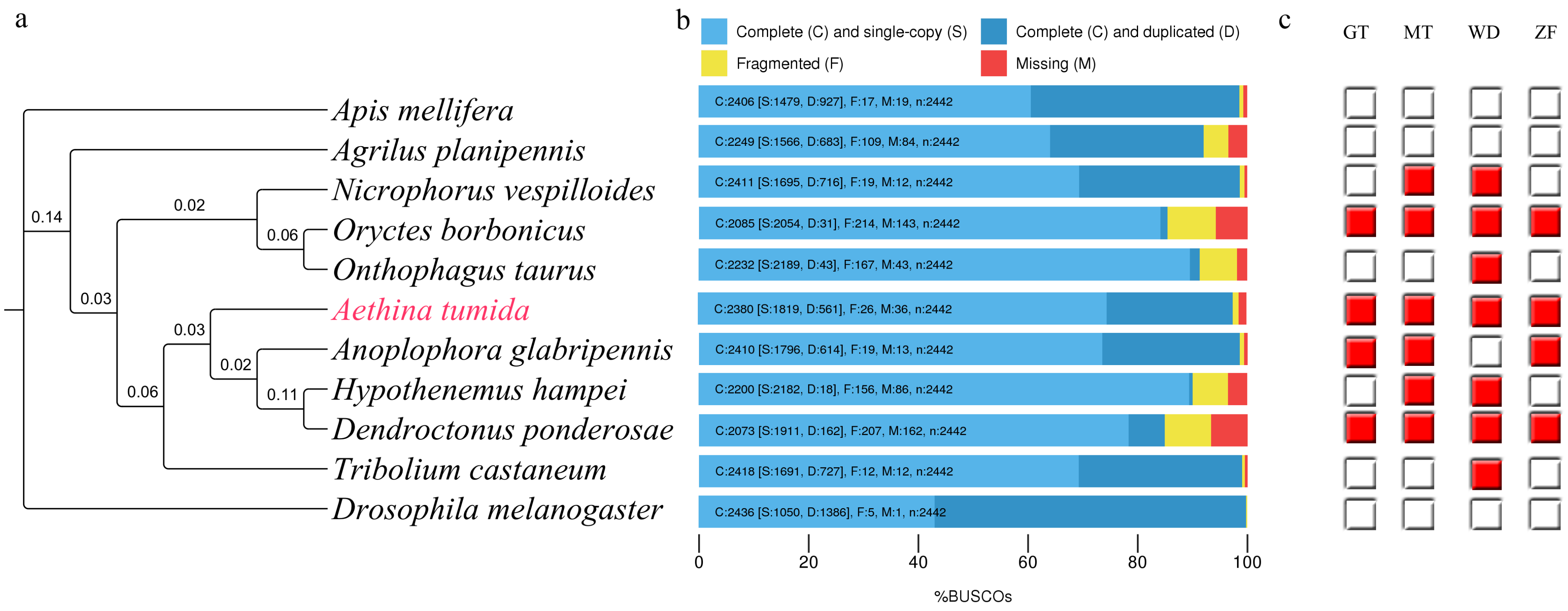

a
b
c
Complete (C) and single-copy (S)
Complete (C) and duplicated (D)
Fragmented (F)
Missing (M)
GT
MT
WD
ZF
0.14
0.03
0.02
0.06
0.03
0.06
0.02
0.11
Apis mellifera
Agrilus planipennis
Nicrophorus vespilloides
Oryctes borbonicus
Onthophagus taurus
Aethina tumida
Anoplophora glabripennis
Hypothenemus hampei
Dendroctonus ponderosae
Tribolium castaneum
Drosophila melanogaster
C:2406 [S:1479, D:927], F:17, M:19, n:2442
C:2249 [S:1566, D:683], F:109, M:84, n:2442
C:2411 [S:1695, D:716], F:19, M:12, n:2442
C:2085 [S:2054, D:31], F:214, M:143, n:2442
C:2232 [S:2189, D:43], F:167, M:43, n:2442
C:2380 [S:1819, D:561], F:26, M:36, n:2442
C:2410 [S:1796, D:614], F:19, M:13, n:2442
C:2200 [S:2182, D:18], F:156, M:86, n:2442
C:2073 [S:1911, D:162], F:207, M:162, n:2442
C:2418 [S:1691, D:727], F:12, M:12, n:2442
C:2436 [S:1050, D:1386], F:5, M:1, n:2442
0
20
40
60
80
100
%BUSCOs

Figure 2

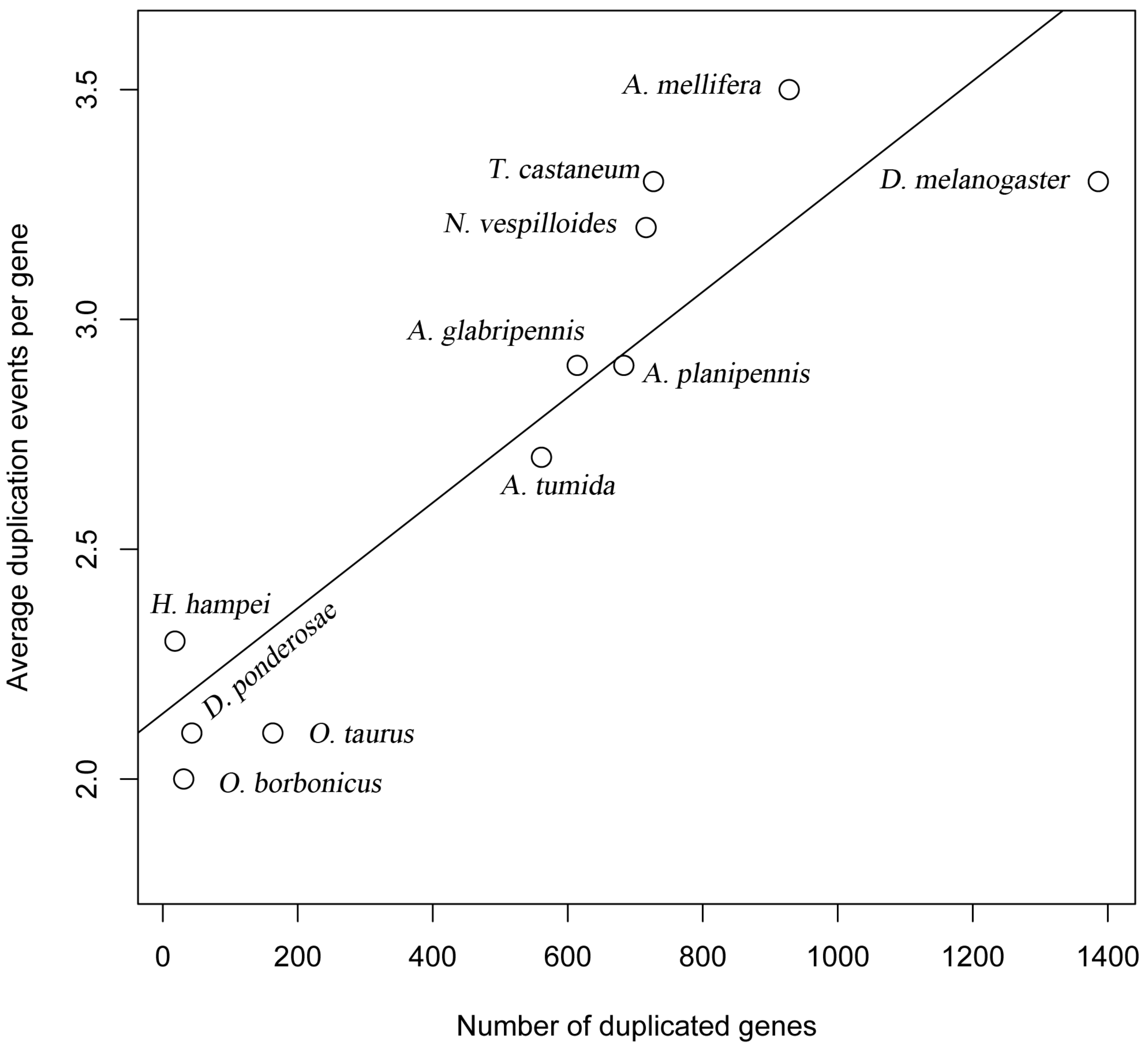
A. mellifera
T. castaneum
D. melanogaster
N. vespilloides
A. glabripennis
A. planipennis
A. tumida
H. hampei
D. ponderosae
O. taurus
O. borbonicus
3.5
3.0
2.5
2.0
Average duplication events per gene
0
200
400
600
800
1000
1200
1400
Number of duplicated genes

Figure 3

Family	ALB	CBB	DPOND	NIC	ORY	SHB	TCAS
GH1	59	21	25	8	2	8	13
GH2	5	2	3	8	1	5	6
GH5	6	2	2	0	0	0	0
GH9	1	0	0	1	1	0	1
GH10	0	2	0	0	0	0	0
GH13	1	0	0	0	0	0	0
GH15	2	2	2	4	1	3	2
GH16	4	5	11	1	3	7	3
GH18	25	19	11	18	13	18	22
GH20	8	8	10	14	8	8	10
GH22	7	0	0	0	0	0	0
GH28	17	7	23	0	0	0	0
GH30	7	1	2	3	1	8	5
GH31	17	6	10	6	7	11	8
GH32	2	0	2	0	0	0	0
GH35	10	5	10	3	0	4	4
GH37	7	0	0	0	0	0	0
GH38	10	7	7	7	5	10	11
GH39	1	0	0	1	0	1	1
GH45	2	3	9	0	0	0	0
GH47	4	4	4	5	3	6	4
GH48	1	4	8	0	0	0	0
GH63	1	1	1	1	1	0	1
GH67	0	0	0	0	0	0	1
GH79	1	1	1	2	1	1	1
GH85	1	1	1	1	0	1	1
GH99	0	0	0	1	0	0	1
**Total**	**199**	**101**	**142**	**84**	**47**	**91**	**95**
	Phytophagous			Other			

Figure 4

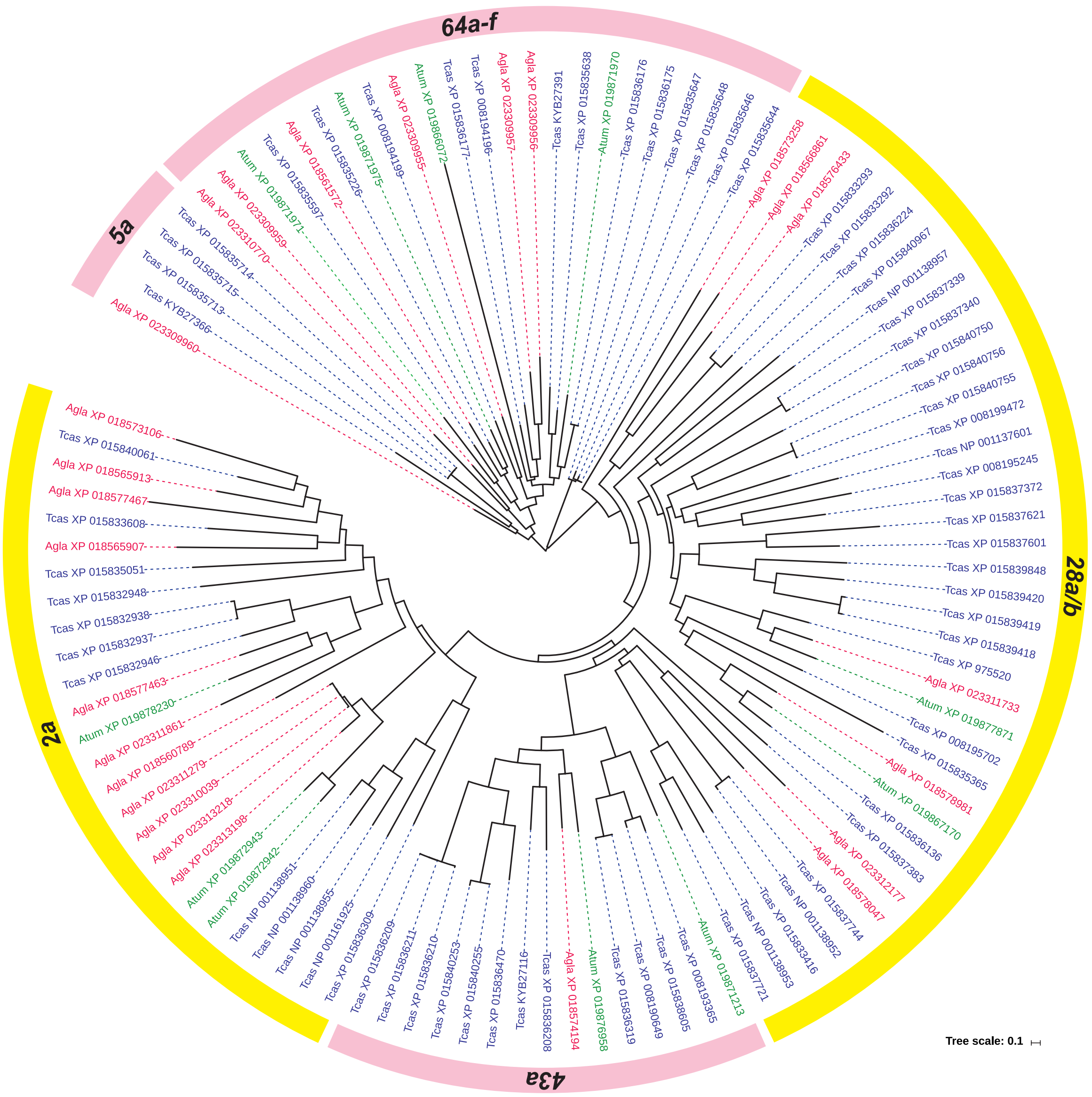
64a-f
5a
28a/b
43a
2a
Agla XP 023309960
Tcas KYB27366
Tcas XP 015835713
Tcas XP 015835715
Tcas XP 015835714
Agla XP 023310770
Agla XP 023309959
Atum XP 019871971
Tcas XP 015835597
Agla XP 018561572
Tcas XP 015835226
Atum XP 019871975
Tcas XP 008194199
Agla XP 023309955
Atum XP 019866072
Tcas XP 015836177
Tcas XP 008194196
Agla XP 023309957
Agla XP 023309956
Tcas KYB27391
Tcas XP 015835638
Atum XP 019871970
Tcas XP 015836176
Tcas XP 015836175
Tcas XP 015835647
Tcas XP 015835648
Tcas XP 015835646
Tcas XP 015835644
Agla XP 018573258
Agla XP 018566861
Agla XP 018576433
Tcas XP 015833293
Tcas XP 015833292
Tcas XP 015836224
Tcas XP 015840967
Tcas NP 001138957
Tcas XP 015837339
Tcas XP 015837340
Tcas XP 015840750
Tcas XP 015840756
Tcas XP 015840755
Tcas XP 008199472
Tcas NP 001137601
Tcas XP 008195245
Tcas XP 015837372
Tcas XP 015837621
Tcas XP 015837601
Tcas XP 015839848
Tcas XP 015839420
Tcas XP 015839419
Tcas XP 015839418
Tcas XP 975520
Agla XP 023311733
Atum XP 019877871
Tcas XP 008195702
Tcas XP 015835365
Agla XP 018578981
Atum XP 019867170
Tcas XP 015836136
Tcas XP 015837383
Agla XP 023312177
Agla XP 018578047
Tcas XP 015837744
Tcas NP 001138952
Tcas XP 015833416
Tcas NP 001138953
Tcas XP 015837721
Atum XP 019871213
Tcas XP 008193365
Tcas XP 015838605
Tcas XP 008190649
Tcas XP 015836319
Atum XP 019876958
Agla XP 018574194
Tcas XP 015836208
Tcas KYB27116
Tcas XP 015836470
Tcas XP 015840255
Tcas XP 015840253
Tcas XP 015836210
Tcas XP 015836211
Tcas XP 015836209
Tcas XP 015836309
Tcas NP 001161925
Tcas NP 001138955
Tcas NP 001138960
Tcas NP 001138951
Atum XP 019872942
Atum XP 019872943
Agla XP 023313198
Agla XP 023313218
Agla XP 023310039
Agla XP 023311279
Agla XP 018560789
Agla XP 023311861
Atum XP 019878230
Agla XP 018577463
Tcas XP 015832946
Tcas XP 015832937
Tcas XP 015832938
Tcas XP 015832948
Tcas XP 015835051
Agla XP 018565907
Tcas XP 015833608
Agla XP 018577467
Agla XP 018565913
Tcas XP 015840061
Agla XP 018573106
Tree scale: 0.1

Figure 5

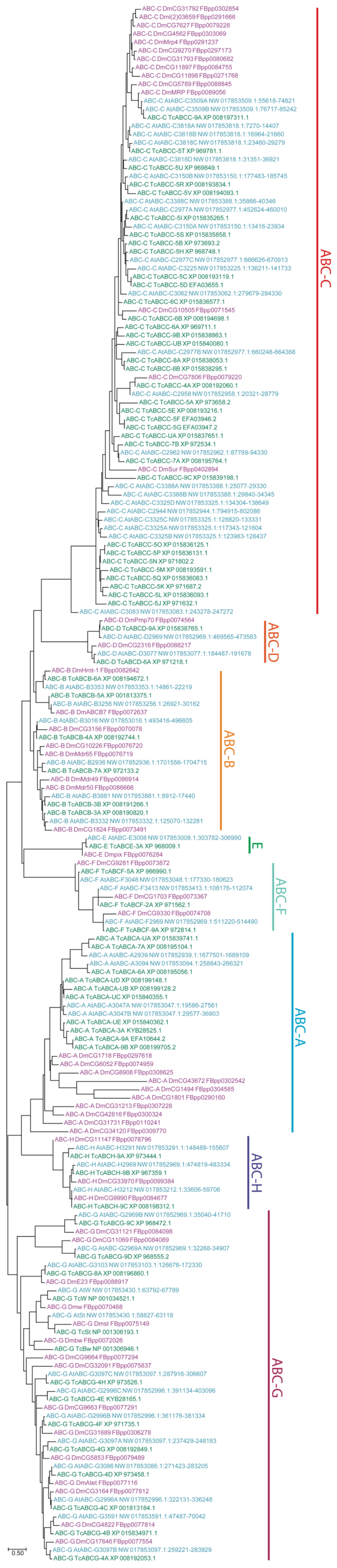

ABC-C DmCG31792 FBpp0302854
ABC-C Dml(2)03659 FBpp0291666
ABC-C DmCG7627 FBpp0079228
ABC-C DmCG4562 FBpp0303069
ABC-C DmMrp4 FBpp0291237
ABC-C DmCG9270 FBpp0297173
ABC-C DmCG31793 FBpp0080682
ABC-C DmCG11897 FBpp0084755
ABC-C DmCG11898 FBpp0271768
ABC-C DmCG5789 FBpp0088845
ABC-C DmMRP FBpp0089056
ABC-C AtABC-C3509A NW 017853509.1:55618-74821
ABC-C AtABC-C3509B NW 017853509.1:76717-85242
ABC-C TcABCC-9A XP 008197311.1
ABC-C AtABC-C3818A NW 017853818.1:7270-14407
ABC-C AtABC-C3818B NW 017853818.1:16964-21660
ABC-C AtABC-C3818C NW 017853818.1:23460-29279
ABC-C TcABCC-5T XP 969781.1
ABC-C AtABC-C3818D NW 017853818.1:31351-36921
ABC-C TcABCC-5U XP 969849.1
ABC-C AtABC-C3150B NW 017853150.1:177483-185745
ABC-C TcABCC-5R XP 008193834.1
ABC-C TcABCC-5V XP 008194093.1
ABC-C AtABC-C3388C NW 017853388.1:35866-40346
ABC-C AtABC-C2977A NW 017852977.1:452624-460010
ABC-C TcABCC-5I XP 015835265.1
ABC-C AtABC-C3150A NW 017853150.1:13416-23934
ABC-C TcABCC-5S XP 015835858.1
ABC-C TcABCC-5B XP 973693.2
ABC-C TcABCC-5H XP 968748.1
ABC-C AtABC-C2977C NW 017852977.1:666626-670913
ABC-C AtABC-C3225 NW 017853225.1:136211-141733
ABC-C TcABCC-5C XP 008193119.1
ABC-C TcABCC-5D EFA03655.1
ABC-C AtABC-C3062 NW 017853062.1:279679-284330
ABC-C TcABCC-6C XP 015836577.1
ABC-C DmCG10505 FBpp0071545
ABC-C TcABCC-6B XP 008194698.1
ABC-C TcABCC-6A XP 969711.1
ABC-C TcABCC-9B XP 015838863.1
ABC-C TcABCC-UB XP 015840080.1
ABC-C AtABC-C2977B NW 017852977.1:660248-664368
ABC-C TcABCC-8A XP 015838053.1
ABC-C TcABCC-8B XP 015838295.1
ABC-C DmCG7806 FBpp0079220
ABC-C TcABCC-4A XP 008192060.1
ABC-C AtABC-C2958 NW 017852958.1:20321-28779
ABC-C TcABCC-5A XP 973658.2
ABC-C TcABCC-5E XP 008193216.1
ABC-C TcABCC-5F EFA03946.2
ABC-C TcABCC-5G EFA03947.2
ABC-C TcABCC-UA XP 015837651.1
ABC-C TcABCC-7B XP 972534.1
ABC-C AtABC-C2962 NW 017852962.1:87789-94330
ABC-C TcABCC-7A XP 008195764.1
ABC-C DmSur FBpp0402894
ABC-C TcABCC-9C XP 015839198.1
ABC-C AtABC-C3388A NW 017853388.1:25077-29330
ABC-C AtABC-C3388B NW 017853388.1:29840-34345
ABC-C AtABC-C3325D NW 017853325.1:134304-138649
ABC-C AtABC-C2944 NW 017852944.1:794915-802086
ABC-C AtABC-C3325C NW 017853325.1:128820-133331
ABC-C AtABC-C3325A NW 017853325.1:117343-121604
ABC-C AtABC-C3325B NW 017853325.1:123983-128437
ABC-C TcABCC-5O XP 015836125.1
ABC-C TcABCC-5P XP 015836131.1
ABC-C TcABCC-5N XP 971802.2
ABC-C TcABCC-5M XP 008193591.1
ABC-C TcABCC-5Q XP 015836083.1
ABC-C TcABCC-5K XP 971687.2
ABC-C TcABCC-5L XP 015836093.1
ABC-C TcABCC-5J XP 971632.1
ABC-C AtABC-C3083 NW 017853083.1:243278-247272
ABC-D DmPmp70 FBpp0074564
ABC-D TcABCD-9A XP 015838765.1
ABC-D AtABC-D2969 NW 017852969.1:469565-473583
ABC-D DmCG2316 FBpp0088217
ABC-D AtABC-D3077 NW 017853077.1:184487-191678
ABC-D TcABCD-6A XP 971218.1
ABC-B DmHmt-1 FBpp0082642
ABC-B TcABCB-6A XP 008194672.1
ABC-B AtABC-B3353 NW 017853353.1:14861-22219
ABC-B TcABCB-5A XP 001813375.1
ABC-B AtABC-B3256 NW 017853256.1:26921-30162
ABC-B DmABCB7 FBpp0072637
ABC-B AtABC-B3016 NW 017853016.1:493416-496605
ABC-B DmCG3156 FBpp0070078
ABC-B TcABCB-4A XP 008192744.1
ABC-B DmCG10226 FBpp0076720
ABC-B DmMdr65 FBpp0076719
ABC-B AtABC-B2936 NW 017852936.1:1701556-1704715
ABC-B TcABCB-7A XP 972133.2
ABC-B DmMdr49 FBpp0086914
ABC-B DmMdr50 FBpp0086666
ABC-B AtABC-B3881 NW 017853881.1:8912-17440
ABC-B TcABCB-3B XP 008191266.1
ABC-B TcABCB-3A XP 008190820.1
ABC-B AtABC-B3332 NW 017853332.1:125070-132281
ABC-B DmCG1824 FBpp0073491
ABC-E AtABC-E3008 NW 017853008.1:303782-306990
ABC-E TcABCE-3A XP 968009.1
ABC-E Dmpix FBpp0076284
ABC-F DmCG9281 FBpp0073872
ABC-F TcABCF-5A XP 966990.1
ABC-F AtABC-F3048 NW 017853048.1:177330-180623
ABC-F AtABC-F3413 NW 017853413.1:108176-112074
ABC-F DmCG1703 FBpp0073367
ABC-F TcABCF-2A XP 971562.1
ABC-F DmCG9330 FBpp0074708
ABC-F AtABC-F2969 NW 017852969.1:511220-514490
ABC-F TcABCF-9A XP 972814.1
ABC-A TcABCA-UA XP 015839741.1
ABC-A TcABCA-7A XP 008195104.1
ABC-A AtABC-A2939 NW 017852939.1:1677501-1689109
ABC-A AtABC-A3094 NW 017853094.1:258843-266321
ABC-A TcABCA-6A XP 008195056.1
ABC-A TcABCA-UD XP 008199148.1
ABC-A TcABCA-UB XP 008199128.2
ABC-A TcABCA-UC XP 015840355.1
ABC-A AtABC-A3047A NW 017853047.1:19586-27561
ABC-A AtABC-A3047B NW 017853047.1:29577-36903
ABC-A TcABCA-UE XP 015840362.1
ABC-A TcABCA-3A KYB28525.1
ABC-A TcABCA-9A EFA10644.2
ABC-A TcABCA-9B XP 008199705.2
ABC-A DmCG1718 FBpp0297618
ABC-A DmCG6052 FBpp0074959
ABC-A DmCG8908 FBpp0308625
ABC-A DmCG43672 FBpp0302542
ABC-A DmCG1494 FBpp0304585
ABC-A DmCG1801 FBpp0290160
ABC-A DmCG31213 FBpp0307228
ABC-A DmCG42816 FBpp0300324
ABC-A DmCG31731 FBpp0110241
ABC-A DmCG34120 FBpp0309770
ABC-H DmCG11147 FBpp0078796
ABC-H AtABC-H3291 NW 017853291.1:148489-155607
ABC-H TcABCH-9A XP 973444.1
ABC-H AtABC-H2969 NW 017852969.1:474819-483334
ABC-H TcABCH-9B XP 967359.1
ABC-H DmCG33970 FBpp0099384
ABC-H AtABC-H3212 NW 017853212.1:33606-59706
ABC-H DmCG9990 FBpp0084677
ABC-H TcABCH-9C XP 008198312.1
ABC-G AtABC-G2969B NW 017852969.1:35040-41710
ABC-G TcABCG-9C XP 968472.1
ABC-G DmCG31121 FBpp0084098
ABC-G DmCG11069 FBpp0084089
ABC-G AtABC-G2969A NW 017852969.1:32268-34907
ABC-G TcABCG-9D XP 968555.2
ABC-G AtABC-G3103 NW 017853103.1:126678-172330
ABC-G TcABCG-8A XP 008196860.1
ABC-G DmE23 FBpp0088917
ABC-G AtW NW 017853430.1:63792-67789
ABC-G TcW NP 001034521.1
ABC-G Dmw FBpp0070468
ABC-G AtSt NW 017853430.1:58827-63118
ABC-G Dmst FBpp0075149
ABC-G TcSt NP 001306193.1
ABC-G Dmbw FBpp0072026
ABC-G TcBw NP 001306946.1
ABC-G DmCG9664 FBpp0077294
ABC-G DmCG32091 FBpp0075837
ABC-G AtABC-G3097C NW 017853097.1:287916-306607
ABC-G TcABCG-4H XP 973526.1
ABC-G AtABC-G2996C NW 017852996.1:391134-403096
ABC-G TcABCG-4E KYB28165.1
ABC-G DmCG9663 FBpp0077291
ABC-G AtABC-G2996B NW 017852996.1:361176-381334
ABC-G TcABCG-4F XP 971735.1
ABC-G DmCG31689 FBpp0306278
ABC-G AtABC-G3097A NW 017853097.1:237429-248183
ABC-G TcABCG-4G XP 008192849.1
ABC-G DmCG5853 FBpp0079489
ABC-G AtABC-G3086 NW 017853086.1:271423-283205
ABC-G TcABCG-4D XP 973458.1
ABC-G DmAtet FBpp0077116
ABC-G DmCG3164 FBpp0077812
ABC-G AtABC-G2996A NW 017852996.1:322131-336248
ABC-G TcABCG-4C XP 001813184.1
ABC-G AtABC-G3591 NW 017853591.1:47487-70042
ABC-G DmCG4822 FBpp0077814
ABC-G TcABCG-4B XP 015834971.1
ABC-G DmCG17646 FBpp0077554
ABC-G AtABC-G3097B NW 017853097.1:259221-283929
ABC-G TcABCG-4A XP 008192053.1
0.50
ABC-C
ABC-D
ABC-B
E
ABC-F
ABC-A
ABC-H
ABC-G

Figure 6

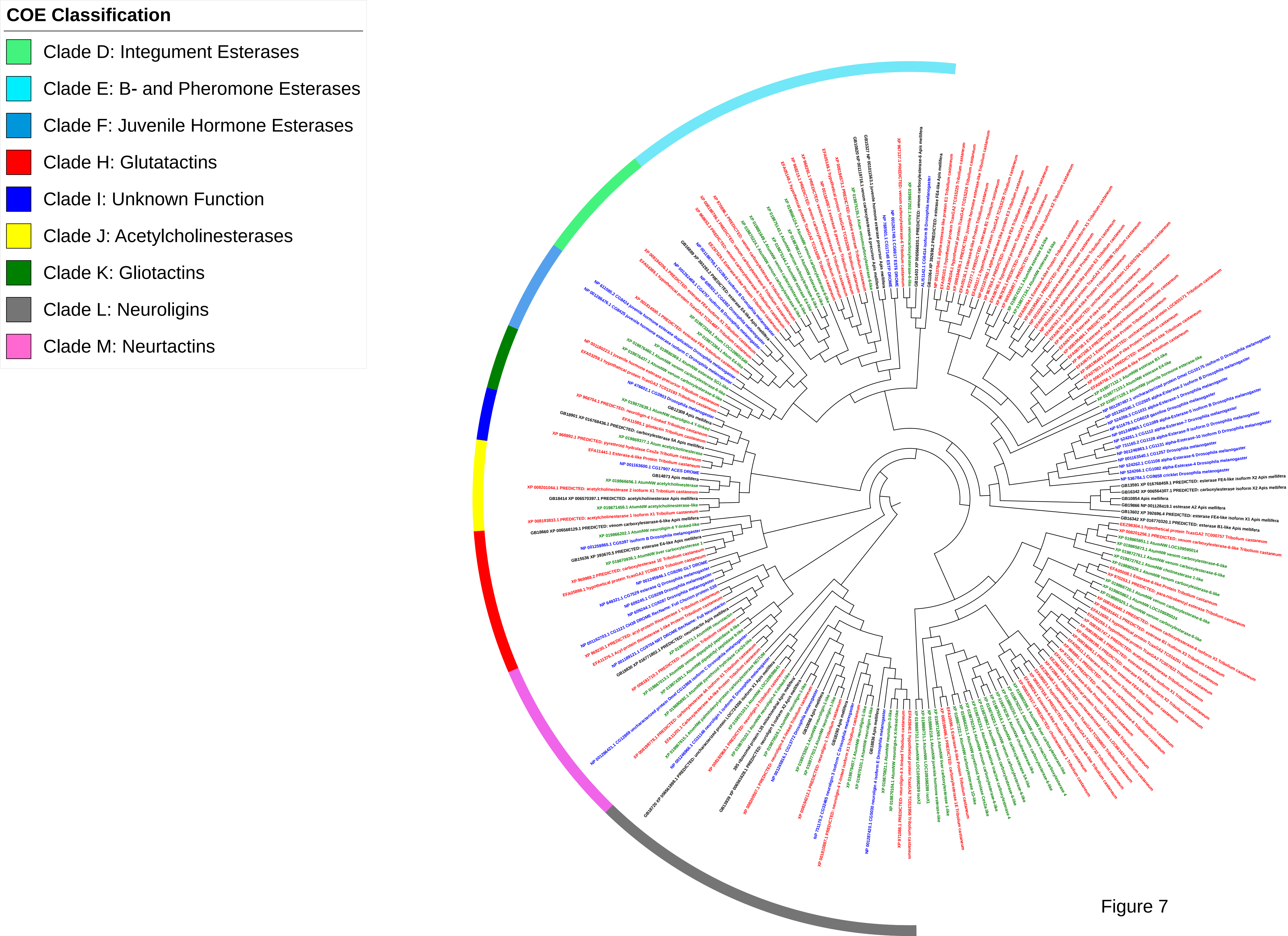
COE Classification
Clade D: Integument Esterases
Clade E: B- and Pheromone Esterases
Clade F: Juvenile Hormone Esterases
Clade H: Glutatactins
Clade I: Unknown Function
Clade J: Acetylcholinesterases
Clade K: Gliotactins
Clade L: Neuroligins
Clade M: Neurtactins

Figure 7

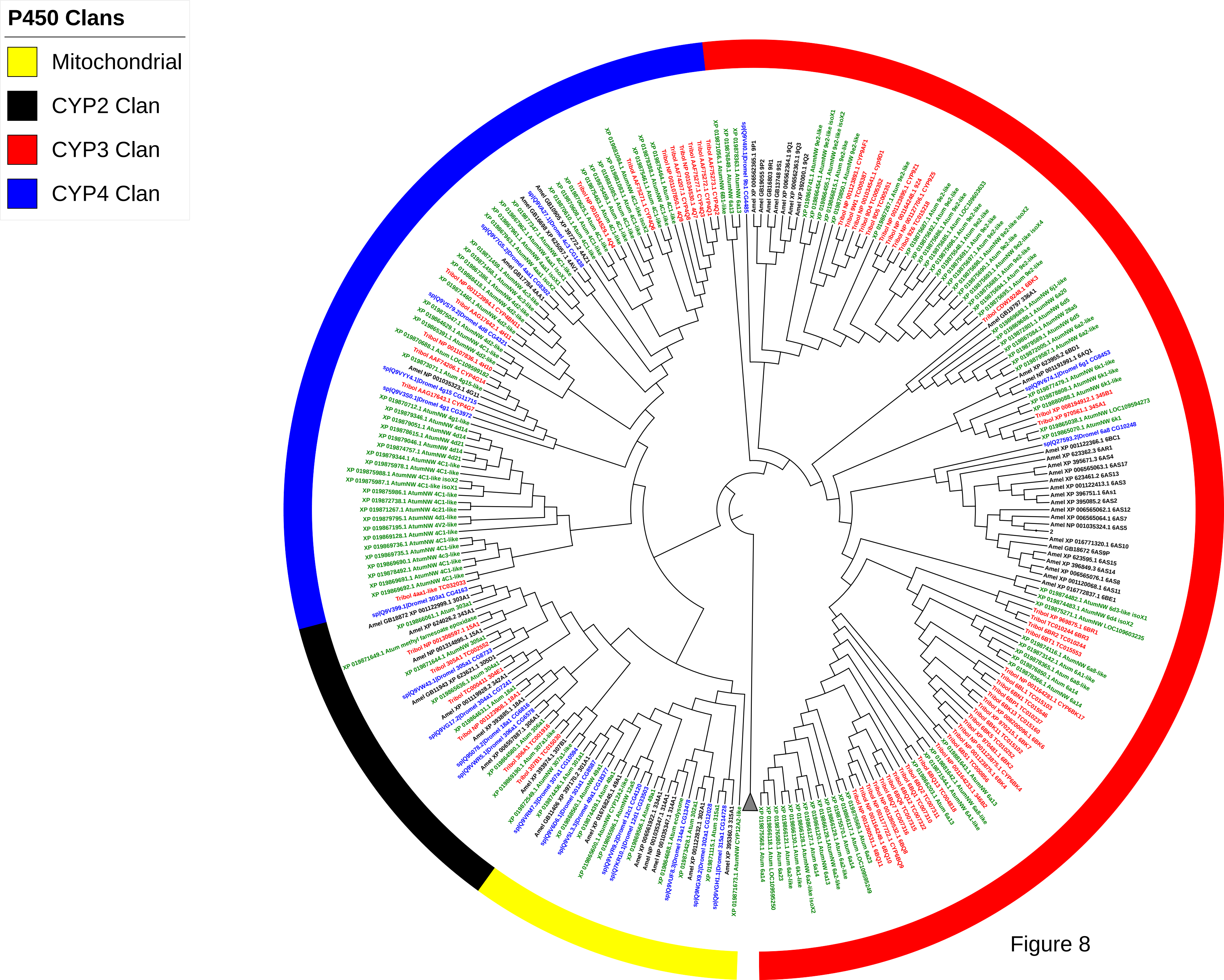

Figure 8

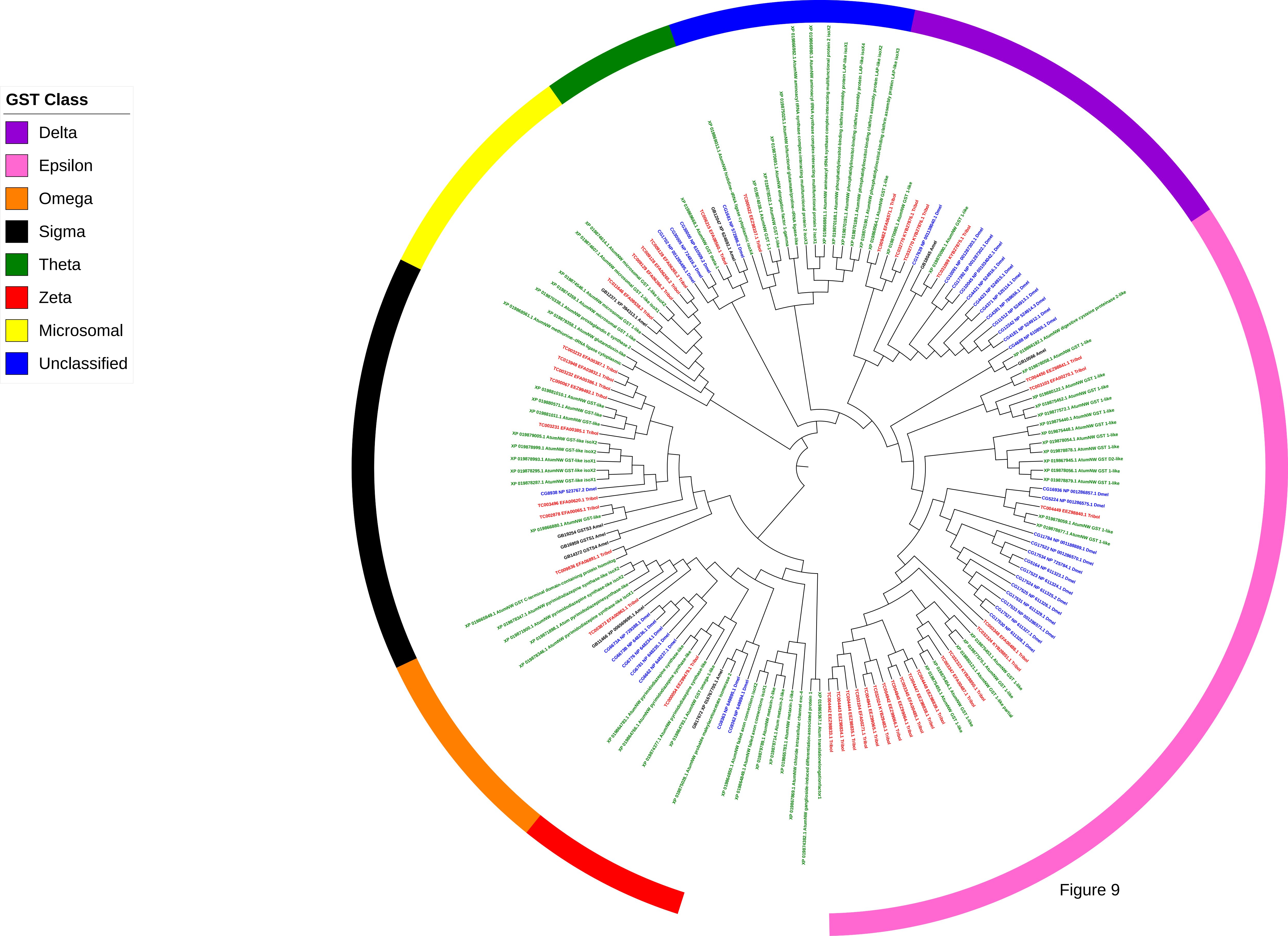
GST Class
Delta
Epsilon
Omega
Sigma
Theta
Zeta
Microsomal
Unclassified

Figure 9

Supplementary Material 1

Click here to access/download
Supplementary Material
SupplementalFile1.docx

Supplementary Material 2

Click here to access/download
Supplementary Material
SupplementalFile2IDs.xlsx

Supplementary Material 3

Click here to access/download
Supplementary Material
SupplementalFile3domains.xlsx
