# Supplementary material for: Genome of the small hive beetle (Aethina tumida, Coleoptera: Nitidulidae), a worldwide parasite of social bee colonies, provides insights into detoxification and herbivory
Source: Gigascience. 2018 Dec 7;7(12):giy138. doi: 10.1093/gigascience/giy138 (PMC6302959; doi:10.1093/gigascience/giy138)
Supplement: Supplemental Files [file giy138_supplemental_files.zip › SupplementalFile1.docx]

Supplementary material

**1.Material and Methods:**

1.1. sampling

SHB larvae and adults were collected March 8, 2014, from a continuous culture of small hive beetles maintained at the USDA-ARS Bee Research Laboratory. The culture was established from 40-50 wild-caught adults, which were housed in a sealed nucleus honey bee hive having several frames containing pollen and nectar as food and oviposition sites, stored in a dark incubator with temperature and humidity at 28 **°**C and 75%, respectively.

1.2. *Gene family size comparisons*

Analyses of gene family representation were performed using NCSS software. Putative orthologs from all 11 insect species were compared with the core BUSCO set of 2242 proteins to determine gains and losses. Contingency analyses were used to determine significant deviations in gene counts. The pattern of expected and observed genes loss was compared with logistic regression. The expected gene loss was inferred from the nearest common ancestor. In this analysis, Emerald ash borer (*A. planipennis*) was used as the first common ancestor it maintained all 4 genes (GT, MT, WD, ZF). Genes lost were represented by 1 while genes maintained were represented by 0 (binary data set). The observed value for small hive beetle is 1 (gene loss for GT), 1 (gene loss for MT), 1 (gene loss for WD), 1 (gene loss for ZF). As the small hive beetle is the nearest common ancestor of Asia longhorned beetle, we would not expect to see the genes lost in small hive beetle in the Asia longhorned beetle. Thus, for the Asian longhorned beetle, the observed value is 1 (gene loss for GT), 1 (gene loss for MT), 0 (gene maintain for WD), 1 (gene loss for ZF) but the expected value is 1 (gene loss for GT), 1 (gene loss for MT), 1 (gene loss for WD), 1 (gene loss for ZF). The expected value for gene loss was calculated with the same method for each of the eight beetle species.

**2. Result and discussion**

2.1 genome completeness


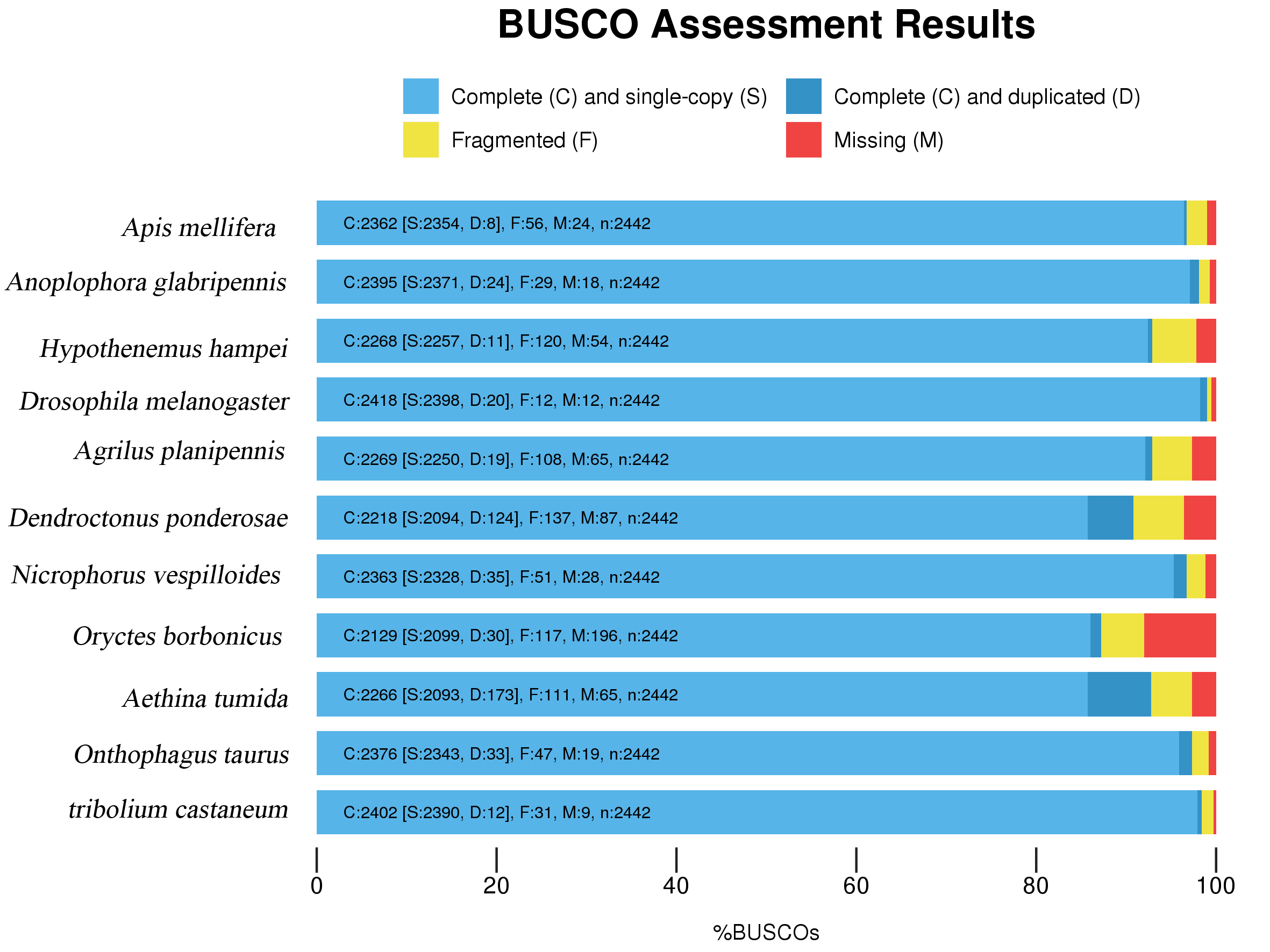


Figure S1. Estimated completeness of small hive beetle and other ten insect species. The completeness of genome assembly of each insect species were assed by aligning to endopterygota sets of benchmarking universal single-copy orthologs (BUSCOs). For the small hive beetle protein, 92.8% of complete BUSCOs were found.

2.2 insecticide candidate gene analysis

*Drosophila* Sodium Channel 1 (DSC1 or NaCP60E) was thought to be a canonical voltage-gated sodium channel (Salkoff et al., 1987). DSC1 is a voltage-gated cation-selective channel (Zhang et al., 2011; Zhou et al., 2004) that is involved with odor detection (Kulkarni et al., 2002)nervous system stability under stress (Zhang et al., 2013), and insecticide sensitivity (Rinkevich et al., 2015; Zhang et al., 2013) among other processes (Dong et al., 2015). Orthologs of this channel exist in invertebrates (Cui et al., 2012). Recent data on the neurophysiological properties of the honey bee DSC1 ortholog showed it was more closely related to calcium channels, prompting it to be classified as a Ca_v4_ channel (Gosselin-Badaroudine et al., 2016).

The predicted protein for AtumSC1 (XP_019868698.1) possesses the characteristic DEEA selectivity filter and MFL fast inactivation motif as seen in other SC1 orthologs (Zhou et al., 2004). There were no optional exons (Zhang et al., 2013) in the predicted protein. Reduced sensitivity to DDT is conferred by an aspartic acid to asparagine mutation at position 1924 in DSC1 (D1924N, (Amichot et al., 1992)). The aspartic acid residue in DSC1 is a threonine in AtumSC1 as it is in DSC1 orthologs in *Tribolium* (XP_015837606.1), honey bee (XP_006572013.1), bumble bee (XP_012173372.1) and carpenter ant (XP_011265121.1).

DSC1 and its orthologs are highly expressed in the antennae and brain (Anholt et al., 1996; Gosselin-Badaroudine et al., 2016), and it has been shown to be critical for odor detection (Kulkarni et al., 2002; Zhang et al., 2013). Small hive beetle adults are attracted to alcohols that are emitted as larval feeding ferments honey, presumably to mate and lay eggs (Hayes et al., 2015). Therefore, a molecule that can specifically inhibit AtumSC1 may interfere with the attraction to fermenting honey and reduce infestation rate.

**Ryanodine Receptor**

The ryanodine receptor mediates the release of intracellular calcium from the endoplasmic reticulum of muscles and neurons resulting in Ca^2+^-dependent intracellular signaling cascades (Sattelle et al., 2008). Chlorantraniliprole (i.e. Rynaxapyr^TM^) is a member of the anthranilic diamide class of insecticides that specifically activate the ryanodine receptor (Lahm et al., 2007).

The ryanodine receptor of small hive beetle is predicted to be a 5112 amino acid protein (XP_019871887.1). The predicted protein has very high identity to other insect ryanodine receptors (a high of 91.2% to *Anoplophora glabripennis* (XP_018566920.1) and a low of 79.8% to *Drosophila melanogaster* (AAM71083.1)). A glycine to glutamic acid mutation (i.e. G4946E) in the extracellular loop between transmembrane domains 2 and 3 is responsible for high levels of resistance to anthranilic diamide insecticides (Troczka et al., 2012). The homologous residue is a glycine in the predicted SHB ryanodine receptor. Considering the high amount of identity with the ryanodine receptors between SHB and honey bee (XP_006569107.1, 84.3%), it is unlikely that anthranilic diamides would be selective for SHB against honey bee.

**GABA-Gated Chloride Channels**

GABA-gated chloride channels are responsible for inhibitory currents in the insect CNS (Bloomquist, 2003). These receptors are the target sites of cyclodiene organochlorine (e.g. chlordane) and phenylpyrazole (e.g. fipronil) classes of insecticides that block receptor function (Casida, 1993). Mutations in the receptors are responsible for resistance to these compounds (ffrench-Constant et al., 2000). Insects possess three GABA-gated chloride channel receptor subunits (Jones & Sattelle, 2006; Jones & Sattelle, 2007).

The predicted Atum_RDL protein (XP_019870942.1) shared 92.8% identity to Tcas_RDL and possesses alternative exons 3a and 6b although alternative exons 3b, 3c and 6a are identified in the genomic sequence (Jones & Sattelle, 2007). Another predicted Atum_RDL protein (XP_019879456.1) only shared 81.0% identity. The latter protein lacked the transmembrane regions so it is likely non-functional. However, the alternative exon use of this gene may have caused a computational error that yielded this presumably non-functional protein as it is bound at the 5’ and 3’ ends by alternative exons 3a and 6a, respectively. The PAR motif immediately preceding the TM2 domain that forms the pore of the channel acts as a selectivity filter for anion-selective receptors is observed in Atum_RDL (Jensen et al., 2005). The RDL subunit also undergoes extensive A-to-I RNA editing that can alter the potency of GABA at the receptor (Jones et al., 2009). Comparison of the predicted mRNA to the BLAST-matched transcriptome sequence (Tarver et al., 2016) showed no evidence of A-to-I RNA editing.

Atum_GRD and Atum_LCCH3 shared 80% identity and 87.9% to the *Tribolium* orthologs, respectively. The predicted protein for Atum_GRD contains the variant 1 splice type (Jones & Sattelle, 2007). Most of the differences in the sequences of these orthologs were in the intracellular linker between TM3 and TM4. Unlike the PAR selectivity motif of RDL, the Atum_GRD and Atim_LCCH3 subunits possess ADR and SAR motifs, respectively. This is consistent with the *Tribolium* orthologs of these proteins (Jones & Sattelle, 2007).

**Glutamate-Gated Chloride Channels**

Five splice variants of the Atum glutamate-gated chloride channels (Atum_GluCl) were predicted. The Atum_GluCl x1, x3, and x5 variants yielded highly similar proteins. Of this group, x3 protein was missing K371, which is in the intracellular linker between TM3 and TM4. While the x1 and x5 proteins were 100% identical, the 5’UTR was different between these transcripts. The x1, x3, and x5 transcripts contained alternative exon 3b, while x2 and x4 possessed alternative exons 3a and 3c respectively. Atum_GluCl exon 3c appears to be a beetle-specific transcript as it has not been reported for other insects besides *Tribolium* (Jones et al., 2010; Jones & Sattelle, 2006; Jones & Sattelle, 2007; Semenov & Pak, 1999).

**pH-sensitive Cl Channels**

Despite possessing all the hallmarks of cys-loop ligand-gated ion channels, a systematic analysis showed that classic neurotransmitters were unable to elicit a response in heterologously-expressed receptors assembled from these genes (Gisselmann et al., 2002). Further investigation showed chloride currents in these channels are inhibited by low extracellular pH and induced by increased temperature and avermectin application. These pH-sensitive chloride channels (pHCl) appears to be relegated to arthropods (Schnizler et al., 2005).

The annotation predicted seven distinct Atum_pHCl proteins that varied due to putative alternative splicing in regions of the intracellular linker between TM3 and TM4. Exon 9a and 9b of the open reading frame exhibits cassette exon where either, both, or neither are present. These transcript types have been previously reported as splice variant 3 or 3a (Jones et al., 2010; Jones & Sattelle, 2006; Jones & Sattelle, 2007; Schnizler et al., 2005), however, the presence of both exons 9a and 9b together in the same transcript as in variants x1-3 is a novelty. These alternative exons add protein kinase C phosphorylation sites. There is an N-myristoylation site on exon 9a. Exon 9b adds a casein kinase II phosphorylation site. The case of the inclusion of both exons 9a and b gives rise to an addition casein kinase II phosphorylation site that spans the conjoined exons. A further source of variation was observed with alternative splicing of the donor and acceptor sites of intron 10. A 12 bp extension of the donor site in transcript x2 adds an additional 4 amino acids (i.e. VNIN) and remains in frame. The acceptor site may be spliced 15 bp upstream to add 5 amino acids (i.e. SCLLQ). These intron splice variants do not add sites for post-translational modifications. Splice variant 4 that modifies resides in the extracellular ligand-binding loop C was not predicted in any of these proteins (Jones et al., 2010; Jones & Sattelle, 2006; Jones & Sattelle, 2007). Up to 16 transcripts are possible using combinations of alternative splicing of exon 9 and intron 10.

Figure S2. Transcript variants of Atum_pHCl. A) Amino acid sequence of splice variants due alternative splicing of exon 9 in Atum_pHCl compared to Tcas_pHCl (Jones & Sattelle, 2007). Sequence motifs in bold are protein kinase-C phosphorylation sites, underlined motifs are casein kinase II phosphorylation sites, and shaded motifs are N-myristoylation sites. B) Amino acid sequence of splice variants due alternative splicing of intron 10 in Atum_pHCl. The corresponding sequence in Tcas_pHCl is identical to the regular splice variant.

A)

Atum_pHCl Exon 9a GENPV**TQR**LPAVLSRIGIILASPL--------------------------------GDKK

Atum_pHCl Exon 9b GENPVTQ---------------------------ETAEQEYYSTFCE**SPR**YT-GDKK

Atum_pHCl Exon 9a+b GENPV**TQR**LPAVLSRIGIILASPLETAEQEYYSTFCE**SPR**YT-GDKK

Atum_pHCl Exon 9x GENPVTQ-----------------------------------------------------------GDKK

Tcas_pHCl Variant 3 GENPVIQRLPAVLSRIGIILASPL---------------------------------GDKK

Tcas_pHCl Variant 3a GENPVIQ----------------------------ETAEQEYYSTFCESPQYRGDKK

B)

Atum_pHCl CAAE------------------VRKK

Atum_pHCl intron 10 donor CAAEVNIN-----------VRKK

Atum_pHCl intron 10 acceptor CAAE---------SCLLQVRKK

Table X. Alternative splicing characteristics of Atum_pHCl transcripts.

| **Atum_pHCl**  **Transcript** | **Exon 9a** | **Exon 9b** | **Intron 10**  **Donor** | **Intron 10**  **Acceptor** |
| --- | --- | --- | --- | --- |
| x1 | X | X | - | X |
| x2 | X | X | X | - |
| x3 | X | X | - | - |
| x4 | - | X | - | X |
| x5 | X | - | - | X |
| x6 | - | - | - | X |
| x7 | - | - | - | - |

**Histamine-gated Chloride Channels**

Histamine is an important neurotransmitter that is involved in photoreception in insects (Sarthy, 1991). Insects possess two genes that encode histamine-gated chloride receptors (HisCl1 and HisCl2 (Gisselmann et al., 2002; Jones et al., 2010; Jones & Sattelle, 2006; Jones & Sattelle, 2007)). Transcripts of these genes are highly expressed in the eye (Zheng et al., 2002) and form pharmacologically and physiologically distinct homomeric receptors (Gisselmann et al., 2002). The Atum_HisCl1 and Atum_HisCl2 proteins possess the PAR motif at the extracellular pore of the channel that regulates anion selectivity (Jensen et al., 2005). The Atum_HisCl1 and Atum_HisCl2 proteins share 86.6% and 84.1% identity with their respective orthologs in Tribolium (Tcas_HisCl1 ABU63602.1; Tcas_HisCl2 ABU63603.1).

Table S1. List of Genbank accession numbers for genes of *A. tumida*.

| **Ortholog** | **Genomic** | **mRNA** | **Protein** |
| --- | --- | --- | --- |
| Atum_Na_v1_ | NW_017853006.1 | XM_020010801.1 | XP_019866360.1 |
| Atum_SC1 | NW_017853065.1 | XM_020013139.1 | XP_019868698.1 |
| Atum_Ryanodine Receptor | NW_017853164.1 | XM_020016328.1 | XP_019871887.1 |
| Atum_Ace1 | NW_017853150.1 | XM_020015897.1 | XP_019871456.1 |
| Atum_Ace2 | NW_017853011.1 | XM_020011097.1 | XP_019866656.1 |
| Atum_nAChR α1 | NW_017853052.1 | XM_020012730.1 | XP_019868289.1 |
| Atum_nAChR α2 | NW_017853541.1 | XM_020021041.1 | XP_019876600.1 |
| Atum_nAChR α3 | NW_017853156.1 | XM_020016025.1 | XP_019871584.1 |
| Atum_nAChR α3 | NW_017853156.1 | XM_020016026.1 | XP_019871585.1 |
| Atum_nAChR α4 | NW_017852971.1 | XM_020008975.1 | XP_019864534.1 |
| Atum_nAChR α5 | NW_017853036.1 | XM_020012025.1 | XP_019867584.1 |
| Atum_nAChR α6 | NW_017853031.1 | XM_020011867.1 | XP_019867426.1 |
| Atum_nAChR α7 | NW_017852949.1 | XM_020024723.1 | XP_019880282.1 |
| Atum_nAChR α8 | NW_017853249.1 | XM_020017664.1 | XP_019873223.1 |
| Atum_nAChR α8 | NW_017853413.1 | XM_020019847.1 | XP_019875406.1 |
| Atum_nAChR α9 | NW_017852989.1 | XM_020010058.1 | XP_019865617.1 |
| Atum_nAChR α10 | NW_017853123.1 | XM_020015177.1 | XP_019870736.1 |
| Atum_nAChR α10 | NW_017855928.1 | XM_020024668.1 | XP_019880227.1 |
| Atum_nAChR α12 | NW_017853036.1 | XM_020012004.1 | XP_019867563.1 |
| Atum_nAChR β1 | NW_017852949.1 | XM_020024745.1 | XP_019880304.1 |
| Atum_RDL | NW_017853131.1 | XM_020015383.1 | XP_019870942.1 |
| Atum_RDL | NW_017854760.1 | XM_020023897.1 | XP_019879456.1 |
| Atum_GRD | NW_017853001.1 | XM_020010564.1 | XP_019866123.1 |
| Atum_LCCH3 | NW_017853073.1 | XM_020013425.1 | XP_019868984.1 |
| Atum_GluCl x1 | NW_017852991.1 | XM_020010165.1 | XP_019865724.1 |
| Atum_GluCl x2 | NW_017852991.1 | XM_020010166.1 | XP_019865725.1 |
| Atum_GluCl x3 | NW_017852991.1 | XM_020010167.1 | XP_019865726.1 |
| Atum_GluCl x4 | NW_017852991.1 | XM_020010168.1 | XP_019865727.1 |
| Atum_GluCl x5 | NW_017852991.1 | XM_020010164.1 | XP_019865723.1 |
| Atum_pHCl x1 | NW_017853592.1 | XM_020021362.1 | XP_019876921.1 |
| Atum_pHCl x2 | NW_017853592.1 | XM_020021363.1 | XP_019876922.1 |
| pHCl x3 | NW_017853592.1 | XM_020021364.1 | XP_019876923.1 |
| Atum_pHCl x4 | NW_017853592.1 | XM_020021365.1 | XP_019876924.1 |
| Atum_pHCl x5 | NW_017853592.1 | XM_020021366.1 | XP_019876925.1 |
| Atum_pHCl x6 | NW_017853592.1 | XM_020021367.1 | XP_019876926.1 |
| Atum_pHCl x7 | NW_017853592.1 | XM_020021368.1 | XP_019876927.1 |
| Atum_HisCl1 | NW_017853431.1 | XM_020020052.1 | XP_019875611.1 |
| Atum_HisCl2 | NW_017853191.1 | XM_020016807.1 | XP_019872366.1 |

Table S2. Percent identity and divergence of the predicted protein sequence of nAChRs in *Aethina tumida* and *Tribolium castaneum*. Putative orthologs are shown in bold.

|  | **Atumα1** | **Atumα2** | **Atumα3** | **Atumα4** | **Atumα5** | **Atumα6** | **Atumα7** | **Atumα8** | **Atumα9** | **Atumα10** | **Atumα12** | **Atumβ1** | **Atumβ divergent** |
| --- | --- | --- | --- | --- | --- | --- | --- | --- | --- | --- | --- | --- | --- |
| **Tcasα1** | **93.0/7.4** | 54.4/68.8 | 58.8/59.0 | 57.0/63.0 | 33.0/142.7 | 38.6/117.0 | 34.4/135.5 | 57.4/61.2 | 24.6/199.0 | 25.5/193.5 | 35.5/130.3 | 42.3/103.2 | 23.6/207.0 |
| **Tcasα2** | 56.3/64.5 | **92.9/7.4** | 54.7/68.2 | 50.3/79.1 | 31.2/152.9 | 37.3/122.2 | 31.9/148.7 | 54.1/59.7 | 23.7/206.0 | 27.3/178.8 | 33.6/139.7 | 41.6/105.7 | 22.9/214.0 |
| **Tcasα3** | 60.9/54.7 | 53.9/70.1 | **93.1/7.2** | 71.1/35.5 | 33.8/138.7 | 38.4/117.7 | 33.5/139.9 | 60.8/54.9 | 24.7/198.0 | 26.2/187.3 | 36.1/127.5 | 45.5/92.7 | 22.4/218.0 |
| **Tcasα4** | 55.7/65.9 | 48.6/83.7 | 70.0/38.2 | **96.0/4.1** | 31.9/148.4 | 39.2/114.6 | 32.7/144.4 | 58.7/59.4 | 24.3/201.0 | 26.5/185.0 | 34.2/136.4 | 44.5/95.8 | 22.6/217.0 |
| **Tcasα5** | 33.5/139.8 | 30.3/158.2 | 32.4/145.7 | 31.3/151.8 | **90.3/10.4** | 36.3/126.7 | 30.7/155.5 | 33.3/140.9 | 21.4/230.0 | 24.8/197.0 | **82.0/20.6** | 33.9/138.1 | 22.3/220.0 |
| **Tcasα6** | 39.2/114.4 | 38.2/118.5 | 38.4/117.7 | 39.5/113.4 | 38.0/119.5 | **94.4/5.9** | 66.4/44.5 | 38.4/117.5 | 25.2/196.5 | 27.8/175.1 | 37.6/120.8 | 38.5/117.3 | 21.4/229.0 |
| **Tcasα7** | 38.6/117.1 | 39.2/114.5 | 39.0/115.3 | 37.5/121.2 | 35.3/131.0 | 68.7/40.5 | **83.5/18.7** | 38.6/116.8 | 23.2/211.0 | 24.9/196.0 | 34.3/136.0 | 35.6/129.8 | 16.8/292.0 |
| **Tcasα8** | 59.2/58.1 | 55.1/67.2 | 62.0/52.6 | 59.3/58.0 | 33.0/142.7 | 38.2/118.6 | 31.7/149.6 | **90.0/10.8** | 21.6/228.0 | 26.1/188.9 | 34.8/133.7 | 43.6/98.6 | 21.0/234.0 |
| **Tcasα9** | 22.4/218.0 | 20.7/237.0 | 22.7/215.0 | 22.1/221.0 | 21.0/234.0 | 21.4/230.0 | 20.3/241.0 | 20.9/236.0 | **49.1/82.4** | 26.9/181.7 | 20.9/234.0 | 20.2/243.0 | 19.0/258.0 |
| **Tcasα10** | 27.0/180.9 | 27.3/179.2 | 25.5/193.5 | 25.4/195.1 | 24.4/200.0 | 27.5/177.4 | 26.4/186.2 | 25.5/194.1 | 27.0/181.5 | **67.8/41.9** | 25.5/193.7 | 26.3/186.8 | 27.0/180.9 |
| **Tcasα11** | 58.8/59.0 | 55.1/66.0 | 63.2/50.3 | 60.4/55.7 | 35.1/132.1 | 39.8/112.1 | 33.4/140.3 | 88.5/12.6 | 21.9/223.0 | 26.7/183.8 | 36.5/125.9 | 45.6/92.3 | 21.6/228.0 |
| **Tcasβ1** | 42.9/101.1 | 42.1/104.0 | 45.4/93.0 | 44.0/97.5 | 34.5/135.0 | 37.3/122.1 | 33.5/139.8 | 45.1/64.1 | 21.5/228.0 | 29.2/164.9 | 34.9/133.0 | **98.2/1.8** | 22.8/214.0 |
| **TcasDivergent** | 22.8/214.0 | 20.5/240.0 | 23.6/207.0 | 22.1/220.0 | 20.9/236.0 | 19.5/252.0 | 19.6/250.0 | 23.0/212.0 | 19.8/248.0 | 26.2/187.4 | 19.4/178.6 | 19.2/255.0 | **53.8/70.3** |

2.3 detoxification gene analysis

Table S3: COE in different species by class. Data are from this study and (Claudianos et al., 2006; Dong et al., 2017).

| **COE subfamily** | ***A. tumida*** | ***D. melanogaster*** | ***T. castaneum*** | ***A. mellifera*** |
| --- | --- | --- | --- | --- |
| **Dietary class** |  |  |  |  |
| Clade A |  | 0 | 0 | 8 |
| Clade B | 8 | 2 | 0 | 0 |
| Clade C |  | 11 | 16 | 0 |
| **Hormone processing class** |  |  |  |  |
| Clade D (Integument esterase) | 2 | 3 | 4 | 1 |
| Clade E (Secreted β-esterase) | 7 | 3 | 0 | 3 |
| Clade F (Dipteran JhE) | 1 | 2 | 5 | 0 |
| Clade G (Lepidopteran JhE) |  | 0 | 5 | 1 |
| **Neuro/development class** |  |  |  |  |
| Clade H (Glutactin) |  | 4 | 1 | 1 |
| Clade I (Uncharacterized clade) | 1 | 2 | 9 | 0 |
| Clade J (Acetylcholinesterases) |  | 1 | 2 | 2 |
| Clade K (Gliotactin) |  | 1 | 1 | 2 |
| Clade L (Neuroligins) |  | 4 | 1 | 1 |
| Clade M (Neurotactins) |  | 2 | 5 | 5 |
| Clade N (Neurotactin) |  | 2 | 2 | 1 |
| **Other** |  | 0 | 0 | 0 |
| **Total** | 19 | 35 | 51 | 24 |

Table S4: Number of GST subfamily members in different species. n.d. : not determined in this study. Metaxin-like and Microsomal subfamily not shown. Number are from this study and {Shi, 2012 #60548}(Claudianos et al., 2006; Shi et al., 2012).

|  | Delta | Epsilon | Omega | Sigma | Theta | Zeta | Total |
| --- | --- | --- | --- | --- | --- | --- | --- |
| *A. tumida* | 3 | n.d. | 6 | 6 | 20 | 1 | 36 |
| *D. melanogaster* | 11 | 14 | 5 | 1 | 4 | 2 | 37 |
| *T. castaneum* | 3 | 19 | 3 | 7 | 1 | 1 | 34 |
| *A. mellifera* | 1 | 0 | 1 | 4 | 1 | 1 | 8 |

Table S5: P450 in different species by clan. Data are from this study and (Claudianos et al., 2006; Tribolium Genome Sequencing, 2008)

|  | CYP2 Clan | CYP3 Clan | CYP4 Clan | Mito Clan | Total |
| --- | --- | --- | --- | --- | --- |
| *A. tumida* | 8 | 54 | 39 | 10 | 111 |
| *D. melanogaster* | 6 | 36 | 32 | 11 | 85 |
| *T. castaneum* | 8 | 72 | 45 | 9 | 134 |
| *A. mellifera* | 8 | 28 | 4 | 6 | 46 |
