# Supplementary material for: Genome of the small hive beetle (Aethina tumida, Coleoptera: Nitidulidae), a worldwide parasite of social bee colonies, provides insights into detoxification and herbivory
Source: Gigascience. 2018 Dec 7;7(12):giy138. doi: 10.1093/gigascience/giy138 (PMC6302959; doi:10.1093/gigascience/giy138)
Supplement: Response_to_Reviewer_Comments_Original_Submission.pdf [file giy138_response_to_reviewer_comments_original_submission.pdf]

Author's Response To Reviewer Comments

Close

Reviewer #1: Positive Comments

This looks like a professionally assembled and annotated genome. It fills in an important blank space within the genomic resources of beetles.

Thank you!

Major Comments
1. At is not "basal" to anything. It can help you infer what the LCA might have looked like, but it has had an equal amount of evolution compared with Phytophaga.

Response: We clarify in this abstract passage that Aethina and the nitidulids diverged basally to what are now Phytophaga by saying that this resource provides the closest available outgroup to the highly successful Phytophaga. Specifically “ATUMI thus provides a contrasting view as a neighbor for one of the most successful known animal groups”

2. No evidence is given for metabolic plasticity, just increased copy number of some metabolic genes. Not the same thing.

While we are convinced that gene-family diversity generally leads to flexibility and novel functions in these groups it is true we have not proven this in ATUMI, and we have dropped that speculation from the abstract. We have left discussion of predicted function for specific orthologs in some gene families since these are secure across the insects for which functional analyses are possible. ATUMI is not as conducive to RNAi interference functional knockdowns as other beetles (i.e., such knockdowns are not generally systemic) but we hope these targets will lead to direct tests of function via lack-of-function RNAi assays

3. Why so few shared ortho-groups? Even restricted to SC BUSCO genes, I would still expect several hundred. Dm has the lowest # of SC at ~1050, but only a 1/5 are SC across all species. Seems odd given BUSCO is SC and complete in 90% of species with the clade of interest, by definition. Just struck me as especially low.

Response: As each species was added to the BUSCO, the shared single-copy genes were decreased. This reflects singular gene loss in some lineages and also weaknesses/omissions in the public gene sets of other species. Our dataset includes 11 species ranging form Hymenoptera (ants, wasps, and bees), to Coleoptera (beetles) and Diptera (flies), it is then not surprising to find a low number of shared SC BUSCO genes. The phylogenic tree we generated based on shared SC BUSCO alignments is consistent with previous published trees, which suggests the accurate assignment of SC BUSCO genes.

4. Loss and duplication of core genes from small hive beetle genome section. This seems like a lot to read into the lack of something. Only 11 species were analyzed. Either I missed what is being said or this is a sweeping analysis of very few "samples."

Response: this is a follow up analysis to the duplicated and missing BUSCO genes. First, we

found 337 BUSCO genes were lost from at least two beetle species. Secondly, we mapped these 337 BUSCO genes to the Pfam database. We also mapped the total 2442 BUSCO genes to Pfam database. By analyzing the distribution of function domains between the 337 genes and 2442 genes, no significantly difference was found. But methyltransferase (MT), glycosyltransferase (GT), beta-transducin repeats (WD) and zinc finger (ZF) showed high counts from the 337 BUSCO genes. Thirdly, we calculated the average duplication evens per BUSCO genes using the equation (total number of duplication events) / (duplicated BUSCO genes). We found a significantly positive correlation between the average duplication events per gene and the number of duplicated BUSCO genes. This result suggests that BUSCO is accurately identifying gene families which shos especially labile gene/protein counts, and hence are candidates for lineage-specific novel functions.

5. GH's, Gr's, Nav's, Ace's, GST's, CP450's, etc sections. They are full of a huge amount of information that is not useful to the central message of the sections. Lots of speculation without that leading to specific hypotheses or broader meaning. It was not clear to me why every observed pattern was explained in such detail. Three examples: Line 265-274 can be deleted without any meaning being lost. Much of the information in the opening paragraphs of each sections are not revisited or used in further paragraphs. Line 346, why would that be informative? Not saying it would not be, but I see no particular reason that it would be. Line 453 paragraph, so much to read into so little evidence, only two of the analyzed species were non-beetles.

Thank you for this suggestion, which is valid, we were perhaps overly excited about some of these shifts, and time plus functional evidence will tell whether the predicted changes in function are real. We have reduced all of the gene family vignettes to what we hope are arguments most relevant to this species and its possible control with novel insecticides. Each section was reduced, on average, by one thiord, and we have in the end trimmed 25 references that were less essential for the arguments that remain. The strong focus on detoxification enzymes came in part from the reality that any steps chosen to control these beetles will be hampered by off-target affects on their sensitive hosts (honey bees), so we are posing possible weak points in the beetle. This information, we expect, will help chemists who are currently designing new controls for ATUMI.

Minor Comments

Abstract. The results have nothing to do with what is discussed with the background section.

We have changed this a bit by omitting comments that are not a focus of the current analysis

Abstract. The reader should be given some indication of gene compliment completeness before the manuscript speaks about gene copy numbers.

Thank you, we have added our BUSCO/completeness parameters as part of the abstract, we feel the captured genes in this analysis are complete and allow for the arguments for gene loss, duplication, etc.

Line 59. Awkward sentence about behaviour.

This was changed.

Line 78. Unclear why this paragraph is sandwiched in between two At life history paragraphs.
We have shifted this big-picture paragraph to follow the (much-shortened) review of ATUMI biology.

Line 107. Endnote field code errors. And a few other places throughout.
fixed

Line 107. Genera should be completely spelled out if it is the first word of a sentence.
in the end we have deleted the discussion of Kodamaea fungi.

Line 113. 343.3 million base pairs is not the number given in Table 1.
this has been corrected, the Table is correct

Line 124 How was DNA extracted? Just realized this is not the methods section. Possibly tell reader details can be found below?

This has been in the supplements and is now given in the methods section lines 410+

Line 142. Why was such as old tool, TopHat2, used? There is a whole generation of better tools; HISAT2, STAR, GSNAP (the updated version). TopHat2 consistently underperforms other tools, especially with default parameters (Baruzzo et al., 2017, Nature Methods).
Resonse: Thank you for encouraging this. we re-analyzed the expressed protein coding genes using Hisat2. The mapped mRNA increased from 99.63% to 99.73%. the mapped protein coding genes increased from 99.56% to 99.65%.

Line 150. "The size of the ATUMI genome assembly is similar to that of the red flour beetle (165.9 Mbp)." Your assemble is over double that, reported as 343 Mb.
Response: The actual assembly was only 234 MBp, and this section has been corrected. The sentence has been revised as “The size of the ATUMI genome assembly is larger than the red flour beetle (165.9 Mbp), but much smaller than the more derived Asian longhorn beetle (707.7 Mbp)”.

Line 164. 2444 needs a comma to be consistent with other number in manuscript.
fixed

Line 202. Recalcitrance. Great word.
Thank you

Line 564. I assume default parameters were used with all programs when not stated. Nice thing to say to remove doubt for all software used.
Response: the parameters have now been added.

Figure 6 legend. In the title, ATUMI is not bolded.
fixed

Reviewer #2: Evans et al., sequenced and assembled a draft genome of small hive beetle and

analyzed some gene families based on this genome assembly. This is a very primary work in the filed of genome analysis. I suggested more comparative genomics analysis should be carried out. This manuscript, at its present status, is below the merit of other papers in Gigascience.

Major concerns,

1. The detail procedures of genome sequencing (illumina and PacBio) and genome assembly should be given in detail. How many individuals used for illumina sequencing and how many for PacBio sequencing.
Response: This was an inadvertent omission from the main text, more complete details of both the collections and the DNA extraction methods are now provided, and are also available along with GFF files and fasta files for the assembly and features in the background information for reviewers, thank you.

2. The methods (software and their parameters) of genome assembly should be given in detail

Response: the parameters have all been included now. Additionally, the detailed codes for the assembly have been uploaded as supplemental data for the reviewers.

3. The authors just mentioned that the genome annotation is carried out using NCBI eukaryotic annotation pipeline but without any detail information. This makes the work is hard to be followed.

A more complete citation to the pipeline as well as specific databases and annotations used the infer this gene set are now cited. The NCBI gene set reflects a balancing of assembly and transcript-based evidence with the resources available at NCBI for comparative genomics. This pipeline has proved more effective than in-house pipelines for generating insect gene sets and features.

4. P2 Line 38-40 Conclusion in the abstract. No evidence to support these conclusions. I do not think the author can get any in-depth conclusion based on present analysis.
This statement has been removed

5. Without Treefam or CAFE analysis, please do not make any conclusions just based on the changes of gene numbers.
We have not confirmed the birth and death of paralogs using CAFÉ, in part because this is the first species in it's clade just outside the phytophaga. As more genomes become available for the beetles it will be possible to confirm birth and loss of paralogs.

Minor Concerns,

1. The abbreviations in this manuscript are not standard. It is hard to follow the used abbreviations, such as ATUMI, TTCAST. Please use either English name or Latin name instead.

This naming scheme has been adopted for gene sets in the insects and likely other eukaryotes in the OrthoDb comparative genomics tables and as a precursor to official gene sets. It seemed awkward to some of us as well, but in the end it is the most even and

consistent way to separate the proteins of different species, since a fixed 4-character name is easier to parse out than a variety of common names or species-level names.

2. GH for Glycoside hydrolyses whereas Grs for Gustarory receptors. Please use uniformed abbreviations

We have capitalized both

3. For each gene families, especially for Gustatory Receptors, the authors used too many sentences (for GPCR,s they used one and a half page) to introduce the gene families. However, only several sentences were given to the data in this beetle. This should be revised before it is submitted again.

These sections have been trimmed substantially.

4. The structure of this manuscript is strange. it has sections of "data description" and "Implicatoins". What is the difference between data description and "material and methods". And what is the difference between "implication" and Discussion.

We have reformatted the sections

5. Most words in the section of methods (especially for gene families analysis) are repeats of results. It is unnecessary to repeat each gene family again in the methods. Please summarize the methods.

Thank you, we have tried to be more concise in the methods, this was meant to make it clear where decisions were made on family boundaries but we have done our best to reduce redundant descriptions.

Close
