# Supplementary material for: Genome of the small hive beetle (Aethina tumida, Coleoptera: Nitidulidae), a worldwide parasite of social bee colonies, provides insights into detoxification and herbivory
Source: Gigascience. 2018 Dec 7;7(12):giy138. doi: 10.1093/gigascience/giy138 (PMC6302959; doi:10.1093/gigascience/giy138)
Supplement: Response_to_Reviewer_Comments_Revision_1.pdf [file giy138_response_to_reviewer_comments_revision_1.pdf]

Author's Response To Reviewer Comments

Close

Reviewer reports:
Reviewer #1: I think they authors did an acceptable job addressing my comments. I only have further minor editorial comments.

Comments:

I do not see where overall gene compliment completeness is mentioned in the abstract.

Minor Comments:

Abstract - Introduction of abbreviation before Latin name sees odd.

We corrected this in the first line of the introduction so that now both mentions of ATUMI follow the latin name that generated this acronym

"… size of the ATUMI genome assembly [is] larger …"

fixed

Orthologous group numbers need commas to be consistent with rest of paper.
fixed

Reference 17 - The potato beetle genome is now published.
fixed

Reviewer #2: The authors have addressed most comments raised by the reviewers. The manuscript has been significantly improved. However, the authors did not revise the manuscript as what they said in the replies to the comments. Here are two examples. 1) the authors mentioned "we have added our BUSCO/completeness parameters as part of the abstract, we feel the captured genes in this analysis are complete and allow for the arguments for gene loss, duplication, etc.". However, I did not find it in the revised abstract. 2) In reply to "Line 164. 2444 needs a comma to be consistent with other number in manuscript." the authors said "fixed". However, no comma found in 2444 and other figures in the revised manuscript (Page 8 line 4 -line 10). The authors should carefully revise the manuscript.
Hi we are sorry, we had corrected most of the commas in numbers but missed some, and should be complete now, as is the addition of BUSCO estimates to the abstract. We have also checked to make sure additional comments were addressed as promised.

Close
