# Supplementary material for: Genome of the small hive beetle (Aethina tumida, Coleoptera: Nitidulidae), a worldwide parasite of social bee colonies, provides insights into detoxification and herbivory
Source: Gigascience. 2018 Dec 7;7(12):giy138. doi: 10.1093/gigascience/giy138 (PMC6302959; doi:10.1093/gigascience/giy138)
Supplement: Reviewer_1_Report_(Original_Submission) -- Christopher Cunningham — 5/30/2018 Reviewed [file giy138_reviewer_1_report_(original_submission).pdf]

Reviewer Report

Title: Genome of the small hive beetle (Aethina tumida, Coleoptera: Nitidulidae), a worldwide parasite of social bee colonies, provides insights into detoxification and herbivory

Version: Original Submission **Date:** 5/30/2018

Reviewer name: Christopher Cunningham

Reviewer Comments to Author:

Positive Comments

This looks like a professionally assembled and annotated genome. It fills in an important blank space within the genomic resources of beetles.

Major Comments

1. At is not "basal" to anything. It can help you infer what the LCA might have looked like, but it has had an equal amount of evolution compared with Phytophaga.

2. No evidence is given for metabolic plasticity, just increased copy number of some metabolic genes. Not the same thing.

3. Why so few shared ortho-groups? Even restricted to SC BUSCO genes, I would still expect several hundred. Dm has the lowest # of SC at ~1050, but only a 1/5 are SC across all species. Seems odd given BUSCO is SC and complete in 90% of species with the clade of interest, by definition. Just struck me as especially low.

4. Loss and duplication of core genes from small hive beetle genome section. This seems like a lot to read into the lack of something. Only 11 species were analyzed. Either I missed what is being said or this is a sweeping analysis of very few "samples."

5. GH's, Gr's, Nav's, Ace's, GST's, CP450's, etc sections. They are full of a huge amount of information that is not useful to the central message of the sections. Lots of speculation without that leading to specific hypotheses or broader meaning. It was not clear to me why every observed pattern was explained in such detail. Three examples: Line 265-274 can be deleted without any meaning being lost. Much of the information in the opening paragraphs of each sections are not revisited or used in further paragraphs. Line 346, why would that be informative? Not saying it would not be, but I see no particular reason that it would be. Line 453 paragraph, so much to read into so little evidence, only two of the analyzed species were non-beetles.

Minor Comments

Abstract. The results have nothing to do with what is discussed with the background section.

Abstract. The reader should be given some indication of gene compliment completeness before the manuscript speaks about gene copy numbers.

Line 59. Awkward sentence about behaviour.

Line 78. Unclear why this paragraph is sandwiched in between two At life history paragraphs.

Line 107. Endnote field code errors. And a few other places throughout.

Line 107. Genera should be completely spelled out if it is the first word of a sentence.

Line 113. 343.3 million base pairs is not the number given in Table 1.

Line 124 How was DNA extracted? Just realized this is not the methods section. Possibly tell reader details can be found below?

Line 142. Why was such as old tool, TopHat2, used? There is a whole generation of better tools; HISAT2, STAR, GSNAP (the updated version). TopHat2 consistently underperforms other tools, especially with default parameters (Baruzzo et al., 2017, Nature Methods).

Line 150. "The size of the ATUMI genome assembly is similar to that of the red flour beetle (165.9 Mbp)." Your assemble is over double that, reported as 343 Mb.

Line 164. 2444 needs a comma to be consistent with other number in manuscript.

Line 202. Recalcitrance. Great word.

Line 564. I assume default parameters were used with all programs when not stated. Nice thing to say to remove doubt for all software used.

Figure 6 legend. In the title, ATUMI is not bolded.

Methods

Are the methods appropriate to the aims of the study, are they well described, and are necessary controls included? Choose an item.

Conclusions

Are the conclusions adequately supported by the data shown? Choose an item.

Reporting Standards

Does the manuscript adhere to the journal's guidelines on minimum standards of reporting? Choose an item.

Choose an item.

Statistics

Are you able to assess all statistics in the manuscript, including the appropriateness of statistical tests used? Choose an item.

Quality of Written English

Please indicate the quality of language in the manuscript: Choose an item.

Declaration of Competing Interests

Please complete a declaration of competing interests, considering the following questions:

- Have you in the past five years received reimbursements, fees, funding, or salary from an organisation that may in any way gain or lose financially from the publication of this manuscript, either now or in the future?
- Do you hold any stocks or shares in an organisation that may in any way gain or lose financially from the publication of this manuscript, either now or in the future?
- Do you hold or are you currently applying for any patents relating to the content of the manuscript?
- Have you received reimbursements, fees, funding, or salary from an organization that holds or has applied for patents relating to the content of the manuscript?
- Do you have any other financial competing interests?
- Do you have any non-financial competing interests in relation to this paper?

If you can answer no to all of the above, write 'I declare that I have no competing interests' below. If your reply is yes to any, please give details below.

No

I agree to the open peer review policy of the journal. I understand that my name will be included on my report to the authors and, if the manuscript is accepted for publication, my named report including any attachments I upload will be posted on the website along with the authors' responses. I agree for my report to be made available under an Open Access Creative Commons CC-BY license (http://creativecommons.org/licenses/by/4.0/). I understand that any comments which I do not wish to be included in my named report can be included as confidential comments to the editors, which will not be published.

I agree to the open peer review policy of the journal

To further support our reviewers, we have joined with Publons, where you can gain additional credit to further highlight your hard work (see: https://publons.com/journal/530/gigascience). On publication of this paper, your review will be automatically added to Publons, you can then choose whether or not to claim your Publons credit. I understand this statement.

Yes Choose an item.
