# Supplementary material for: Genome of the small hive beetle (Aethina tumida, Coleoptera: Nitidulidae), a worldwide parasite of social bee colonies, provides insights into detoxification and herbivory
Source: Gigascience. 2018 Dec 7;7(12):giy138. doi: 10.1093/gigascience/giy138 (PMC6302959; doi:10.1093/gigascience/giy138)
Supplement: Reviewer_2_Report_(Original_Submission) -- Fei Li — 6/5/2018 Reviewed [file giy138_reviewer_2_report_(original_submission).pdf]

Reviewer Report

Title: Genome of the small hive beetle (Aethina tumida, Coleoptera: Nitidulidae), a worldwide parasite of social bee colonies, provides insights into detoxification and herbivory

Version: Original Submission **Date:** 6/5/2018

Reviewer name: Fei Li

Reviewer Comments to Author:

Evans et al., sequenced and assembled a draft genome of small hive beetle and analyzed some gene families based on this genome assembly. This is a very primary work in the filed of genome analysis. I suggested more comparative genomics analysis should be carried out. This manuscript, at its present status, is below the merit of other papers in Gigascience.

Major concerns,

1. The detail procedures of genome sequencing (illumina and PacBio) and genome assembly should be given in detail. How many individuals used for illumina sequencing and how many for PacBio sequencing.

2. The methods (software and their parameters) of genome assembly should be given in detail

3. The authors just mentioned that the genome annotation is carried out using NCBI eukaryotic annotation pipeline but without any detail information. This makes the work is hard to be followed.

4. P2 Line 38-40 Conclusion in the abstract. No evidence to support these conclusions. I do not think the author can get any in-depth conclusion based on present analysis.

5. Without Treefam or CAFE analysis, please do not make any conclusions just based on the changes of gene numbers.

Minor Concerns,

1. The abbreviations in this manuscript are not standard. It is hard to follow the used abbreviations, such as ATUMI, TTCAST. Please use either English name or Latin name instead.

2. GH for Glycoside hydrolyses whereas Grs for Gustarory receptors. Please use uniformed abbreviations

3. For each gene families, especially for Gustatory Receptors, the authors used too many sentences (for GPCR,s they used one and a half page) to introduce the gene families. However, only several sentences were given to the data in this beetle. This should be revised before it is submitted again.

4. The structure of this manuscript is strange. it has sections of "data description" and "Implicatoins". What is the difference between data description and "material and methods". And what is the difference between "implication" and Discussion.

5. Most words in the section of methods (especially for gene families analysis) are repeats of results. It is unnecessary to repeat each gene family again in the methods. Please summarize the methods.

Methods

Are the methods appropriate to the aims of the study, are they well described, and are necessary controls included? Choose an item.

Conclusions

Are the conclusions adequately supported by the data shown? Choose an item.

Reporting Standards

Does the manuscript adhere to the journal's guidelines on minimum standards of reporting? Choose an item.

Choose an item.

Statistics

Are you able to assess all statistics in the manuscript, including the appropriateness of statistical tests used? Choose an item.

Quality of Written English

Please indicate the quality of language in the manuscript: Choose an item.

Declaration of Competing Interests

Please complete a declaration of competing interests, considering the following questions:

- Have you in the past five years received reimbursements, fees, funding, or salary from an organisation that may in any way gain or lose financially from the publication of this manuscript, either now or in the future?
- Do you hold any stocks or shares in an organisation that may in any way gain or lose financially from the publication of this manuscript, either now or in the future?
- Do you hold or are you currently applying for any patents relating to the content of the manuscript?
- Have you received reimbursements, fees, funding, or salary from an organization that holds or has applied for patents relating to the content of the manuscript?
- Do you have any other financial competing interests?
- Do you have any non-financial competing interests in relation to this paper?

If you can answer no to all of the above, write 'I declare that I have no competing interests' below. If your reply is yes to any, please give details below.

I declare that I have no competing interests

I agree to the open peer review policy of the journal. I understand that my name will be included on my report to the authors and, if the manuscript is accepted for publication, my named report including any attachments I upload will be posted on the website along with the authors' responses. I agree for my report to be made available under an Open Access Creative Commons CC-BY license (http://creativecommons.org/licenses/by/4.0/). I understand that any comments which I do not wish to be included in my named report can be included as confidential comments to the editors, which will not be published.

I agree to the open peer review policy of the journal

To further support our reviewers, we have joined with Publons, where you can gain additional credit to further highlight your hard work (see: https://publons.com/journal/530/gigascience). On publication of this paper, your review will be automatically added to Publons, you can then choose whether or not to claim your Publons credit. I understand this statement.

Yes Choose an item.
